# Metabolic precision labeling enables selective probing of O-linked *N*-acetylgalactosamine glycosylation

**DOI:** 10.1073/pnas.2007297117

**Published:** 2020-09-28

**Authors:** Marjoke F. Debets, Omur Y. Tastan, Simon P. Wisnovsky, Stacy A. Malaker, Nikolaos Angelis, Leonhard K. R. Moeckl, Junwon Choi, Helen Flynn, Lauren J. S. Wagner, Ganka Bineva-Todd, Aristotelis Antonopoulos, Anna Cioce, William M. Browne, Zhen Li, David C. Briggs, Holly L. Douglas, Gaelen T. Hess, Anthony J. Agbay, Chloe Roustan, Svend Kjaer, Stuart M. Haslam, Ambrosius P. Snijders, Michael C. Bassik, W. E. Moerner, Vivian S. W. Li, Carolyn R. Bertozzi, Benjamin Schumann

**Affiliations:** ^a^Department of Chemistry, Stanford University, Stanford, CA 94305;; ^b^The Chemical Glycobiology Laboratory, The Francis Crick Institute, NW1 1AT London, United Kingdom;; ^c^Stem Cell and Cancer Biology Laboratory, The Francis Crick Institute, NW1 1AT London, United Kingdom;; ^d^Proteomics Science Technology Platform, The Francis Crick Institute, NW1 1AT London, United Kingdom;; ^e^Department of Chemistry, University of California, Berkeley, CA 94720;; ^f^Peptide Chemistry Science Technology Platform, The Francis Crick Institute, NW1 1AT London, United Kingdom;; ^g^Department of Life Sciences, Imperial College London, SW7 2AZ London, United Kingdom;; ^h^Department of Chemistry, Imperial College London, W12 0BZ London, United Kingdom;; ^i^Signalling and Structural Biology Laboratory, The Francis Crick Institute, NW1 1AT London, United Kingdom;; ^j^Mycobacterial Metabolism and Antibiotic Research Laboratory, The Francis Crick Institute, NW1 1AT London, United Kingdom;; ^k^Department of Genetics, Stanford University, Stanford, CA 94305;; ^l^Program in Cancer Biology, Stanford University, Stanford, CA 94305;; ^m^Structural Biology Science Technology Platform, The Francis Crick Institute, NW1 1AT London, United Kingdom;; ^n^Howard Hughes Medical Institute, Stanford, CA 94305

**Keywords:** glycosylation, bioorthogonal, glycosyltransferase, mucin

## Abstract

Protein glycosylation events that happen early in the secretory pathway are often dysregulated during tumorigenesis. These events can be probed, in principle, by monosaccharides with bioorthogonal tags that would ideally be specific for distinct glycan subtypes. However, metabolic interconversion into other monosaccharides drastically reduces such specificity in the living cell. Here, we use a structure-based design process to develop the monosaccharide probe *N*-(*S*)-azidopropionylgalactosamine (GalNAzMe) that is specific for cancer-relevant Ser/Thr(O)–linked *N*-acetylgalactosamine (GalNAc) glycosylation. By virtue of a branched *N*-acylamide side chain, GalNAzMe is not interconverted by epimerization to the corresponding *N*-acetylglucosamine analog by the epimerase *N*-acetylgalactosamine–4-epimerase (GALE) like conventional GalNAc–based probes. GalNAzMe enters O-GalNAc glycosylation but does not enter other major cell surface glycan types including Asn(N)-linked glycans. We transfect cells with the engineered pyrophosphorylase mut-AGX1 to biosynthesize the nucleotide-sugar donor uridine diphosphate (UDP)-GalNAzMe from a sugar-1-phosphate precursor. Tagged with a bioorthogonal azide group, GalNAzMe serves as an O-glycan–specific reporter in superresolution microscopy, chemical glycoproteomics, a genome-wide CRISPR-knockout (CRISPR-KO) screen, and imaging of intestinal organoids. Additional ectopic expression of an engineered glycosyltransferase, “bump-and-hole” (BH)–GalNAc-T2, boosts labeling in a programmable fashion by increasing incorporation of GalNAzMe into the cell surface glycoproteome. Alleviating the need for GALE-KO cells in metabolic labeling experiments, GalNAzMe is a precision tool that allows a detailed view into the biology of a major type of cancer-relevant protein glycosylation.

## Significance

Most human secreted and cell surface proteins are modified by Ser/Thr(O)-linked glycosylation with *N*-acetylgalactosamine (O-GalNAc). While of fundamental importance in health and disease, O-GalNAcglycosylation is technically challenging to study because of a lack of specific tools for biological assays. Here, we design an O-GalNAc–specific reporter molecule termed uridine diphosphate (UDP)–*N*-(*S*)-azidopropionylgalactosamine (GalNAzMe) to selectively label O-GalNAc glycoproteins in living human cells. UDP-GalNAzMe can be biosynthesized in cells by transfection with an engineered metabolic enzyme and is compatible with a range of experiments in quantitative biology to broaden our understanding of glycosylation. We demonstrate that labeling is genetically programmable by ectopic expression of a mutant glycosyltransferase, “bump-and-hole”–GalNAc-T2, allowing application to experiments with low inherent sensitivity.

**Fig. 1.**
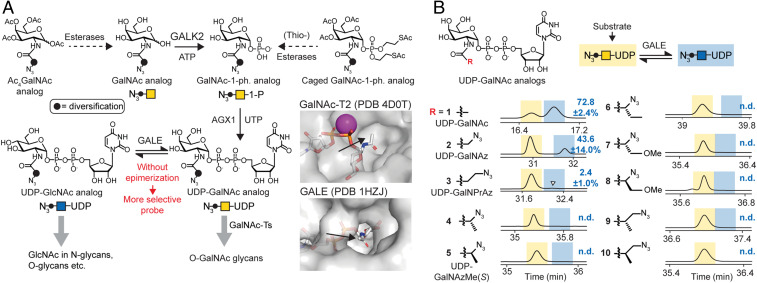
Design of an O-GalNAc–specific metabolic labeling reagent. (*A*) Rationale of probe design. UDP-GalNAc analogs that are not epimerized to the corresponding UDP-GlcNAc derivatives are O-GalNAc specific by design. Derivatives are delivered to the living cell by virtue of per-acetylated or phosphotriester-caged precursors. Compounds with a sterically congested diversification may be resistant to GALE-mediated epimerization but are accepted by GalNAc-Ts. *Inset* shows UDP-GlcNAc and UDP-GalNAc binding by GalNAc-T2 and GALE, respectively. (*B*) In vitro epimerization as assessed by ion-pair HPLC. Retention times of UDP-GalNAc analogs (yellow) and UDP-GlcNAc analogs (blue) are highlighted based on retention times of standards or epimerization reactions with 50-fold higher GALE concentration (*SI Appendix*, Fig. S1*B*). Arrowhead depicts epimerization of compound **3**. Numbers are percentage epimerization as assessed by peak integration as means ± SD of three independent replicates or not detected (n.d.). Traces depict relative intensity of absorbance at 260 nm. Data are from one representative of three independent experiments and were reproduced using lysates of WT cells as a source of GALE or GALE-KO cells as a negative control in two independent replicates (*SI Appendix*, Fig. S1*B*). ATP, adenosine triphosphate; PDB, protein database identifier.

The many facets of cellular glycosylation in health and disease demand methods of visualizing and characterizing glycoconjugates. These methods are essential for the development of next-generation therapeutics and diagnostics that depend on understanding glycosylation of target biomolecules (1, 2). A number of modern experimental techniques have shaped our understanding of biology, such as advanced microscopy, mass spectrometry (MS) (glyco-)proteomics, and genome-wide CRISPR-knockout (CRISPR-KO) screens. Application of these techniques to glycobiology relies on the suitability of detection reagents. Antibodies, lectins, and engineered hydrolases have been instrumental but are somewhat restricted to sterically accessible epitopes (3–7). Monosaccharides with bioorthogonal, chemically editable functionalities have allowed a complementary view into glycobiology by entering early glycosylation events (8–12). For instance, the first azide-containing *N*-acetylgalactosamine (GalNAc) analog, GalNAz, and subsequent renditions made it possible to probe core glycosylation that is difficult to reach with protein-based reagents (9). GalNAc analogs are fed to cells as esterase-sensitive precursors and converted into the corresponding uridine diphosphate (UDP) sugar donors by the kinase GALK2 and the pyrophosphorylase AGX1 (Fig. 1*A*). The cellular glycosylation machinery then incorporates tagged monosaccharides into glycoconjugates where they are reacted with reporter moieties such as fluorophores or biotin by either copper(I)-catalyzed azide-alkyne cycloaddition (CuAAC) or strain-promoted azide-alkyne cycloaddition (SPAAC) (13, 14).

A particular drawback of most current chemically modified monosaccharides is their low specificity: UDP-GalNAz enters mucin-type (Ser/Thr–*N*-acetylgalactosamine [O-GalNAc]) glycans but is also converted to the corresponding GlcNAc derivative UDP-GlcNAz by the cellular UDP-GlcNAc/*N*-acetylgalactosamine–4-epimerase (GALE) (Fig. 1*A*) (15). UDP-GlcNAz then enters N-linked glycans as well as other GlcNAc-containing glycans (15, 16). Other GalNAc analogs are presumably interconverted into GlcNAc analogs in a similar fashion, but their metabolic fate can be variable (17).

Forays have been made into developing reagents that are specific for the structurally simple nucleocytoplasmic O-GlcNAc modification (18–20). However, no such reagents are available to specifically probe the complex cell surface O-GalNAc glycosylation that has fundamental relevance in many aspects of cancer (21, 22).

Studying O-GalNAc glycoproteins by MS-based glycoproteomics is complicated by glycan heterogeneity, the lack of O-glycosylation consensus sequences, and selective enrichment tools. Further complexity is added by the interplay of 20 GalNAc transferase (GalNAc-T1…-T20) isoenzymes that mediate the first O-GalNAc biosynthesis step (23, 24). In a “bump-and-hole” (BH) approach, we have recently engineered GalNAc-Ts to carry a double mutation to preferentially accept UDP-GalNAc analogs with bulky chemical, editable tags (25, 26). Although this technique produced bioorthogonal reporters with great specificity for particular GalNAc-T isoenzymes, epimerization of GalNAc analogs by GALE was still a challenge and resulted in background N-glycan labeling (26).

A strategy to visualize O-GalNAc glycans based on metabolic labeling is the use of GalNAz in GALE-deficient cells that cannot epimerize UDP-GalNAz (15, 27). However, this strategy is of limited use as GALE deficiency heavily interferes with glycan metabolism and might therefore not be easily adaptable to multicellular model systems such as organoids (28, 29).

Here, we report the GalNAc-specific bioorthogonal metabolic labeling reagent *N*-(*S*)-azidopropionylgalactosamine (GalNAzMe). Using a collection of synthetic azide-containing UDP-GalNAc analogs and structure-informed probe design, we find that branched acylamide side chains confer resistance to GALE-mediated epimerization and therefore, eliminate the need of using *N*-acetylgalactosamine–4-epimerase–knockout (GALE-KO) cells for metabolic labeling experiments. We use a caged precursor of the nucleotide-sugar UDP-GalNAzMe to probe O-GalNAc glycosylation in a range of experimental conditions, including superresolution microscopy, chemical MS glycoproteomics, a genome-wide CRISPR-KO screen, and intestinal organoid imaging. GalNAzMe labeling can be enhanced in the presence of a BH-GalNAc-T2 double mutant, further expanding the use of this monosaccharide in glycobiology experiments. Precision tools such as GalNAzMe are essential to uncover the fine details of cellular glycosylation.

## Results

### Probe Design.

We envisioned that a chemically modified UDP-GalNAc analog would be O-GalNAc specific if it was 1) not epimerized to the UDP-GlcNAc analog by GALE and 2) used by either wild-type (WT) or BH-engineered GalNAc-Ts to be incorporated into cell surface O-GalNAc glycans (Fig. 1*A*). Investigation of the cocrystal structure of human GalNAc-T2 and UDP-GalNAc suggested that the GalNAc acetamide group is embedded in a pocket that allows for some three-dimensional freedom (Fig. 1*A*). A similar binding site architecture was observed in the crystal structures of GalNAc-T10 and GalNAc-T7 with GalNAc or UDP-GalNAc in their active sites, respectively (*SI Appendix*, Fig. S1*A*) (30, 31). In contrast to GalNAc-Ts, human GALE accommodates the acetamide in a long, narrow cavity, as evidenced in a cocrystal structure of GALE with UDP-GlcNAc (Fig. 1*A*). This difference in substrate recognition prompted us to explore the chemical determinants of GALE-mediated epimerization by in vitro assays. We expressed human GALE in insect cells and used a collection of UDP-GalNAc **1** as well as analogs **2** to **10** with azide-containing acylamide groups as substrates for epimerization (25, 32). Ion-pair high-performance liquid chromatography (HPLC) was used to separate the UDP-GalNAc analogs from their UDP-GlcNAc epimers (33). UDP-GalNAc **1**, UDP-GalNAz **2**, and uridine diphosphate-*N*-3-azidopropionate **3**, which we term UDP-GalNPrAz, were epimerized to the corresponding UDP-GlcNAc derivatives (Fig. 1*B*). In contrast, all compounds containing a branched acylamide moiety (**4** to **10**) were resistant toward epimerization under these conditions, evident by the absence of a peak with a later retention time in HPLC chromatograms. To rule out coelution of both epimers, we used commercial and newly synthesized UDP-GlcNAc–derived epimers of **1** (UDP-GlcNAc), **2** (UDP-GlcNAz), **3** (UDP-GlcNPrAz), and **5** (UDP-GlcNAzMe) as standards and confirmed a marked difference in retention time (*SI Appendix*, Fig. S1*B*). GALE-mediated epimerization of linear but not branched UDP-GalNAc analogs was corroborated by performing the reactions in the presence of cytosolic extracts of K-562 cells with or without functional GALE (control single-guide RNA [sgRNA] and GALE-KO, respectively) (26). An extract containing GALE epimerized compounds **1** to **3,** but not **4** to **10**, whereas an extract from GALE-KO cells was devoid of epimerization in all cases (*SI Appendix*, Fig. S1*C*). When assessing the scope of GALE reactivity, we succeeded in forcing branched analogs **4** to **9** to epimerize by increasing the concentration of purified GALE 50-fold in vitro (*SI Appendix*, Fig. S1*C*). These data indicate that branched acylamide side chains confer resistance to epimerization unless the concentration of GALE is increased to unphysiologically high levels.

We then chose one of the structurally simplest branched UDP-GalNAc analogs in our collection to assess turnover by GalNAc-Ts. We had previously found UDP-GalNAzMe **5** to be a substrate of WT-GalNAc-T2 (25) and confirmed acceptance by WT-GalNAc-T1, -T7, and -T10 in in vitro glycosylation experiments of peptide substrates (*SI Appendix*, Fig. S2*A*) (25). UDP-GalNAzMe **5** displayed a very similar activity profile to the well-known substrates UDP-GalNAc **1** and UDP-GalNAz **2**, albeit at lower incorporation levels. The azide-containing molecules **2** and **5** were used by WT-T1 and -T2 to glycosylate proteins in a membrane protein preparation, as visualized by CuAAC with a biotin-alkyne and fluorescently labeled streptavidin by western blot (*SI Appendix*, Fig. S2*C*). These data indicate that UDP-GalNAzMe is a viable substrate for GalNAc-Ts to generate azide-tagged O-GalNAc glycans.

### Labeling the Cellular O-GalNAc Glycome.

**Fig. 2.**
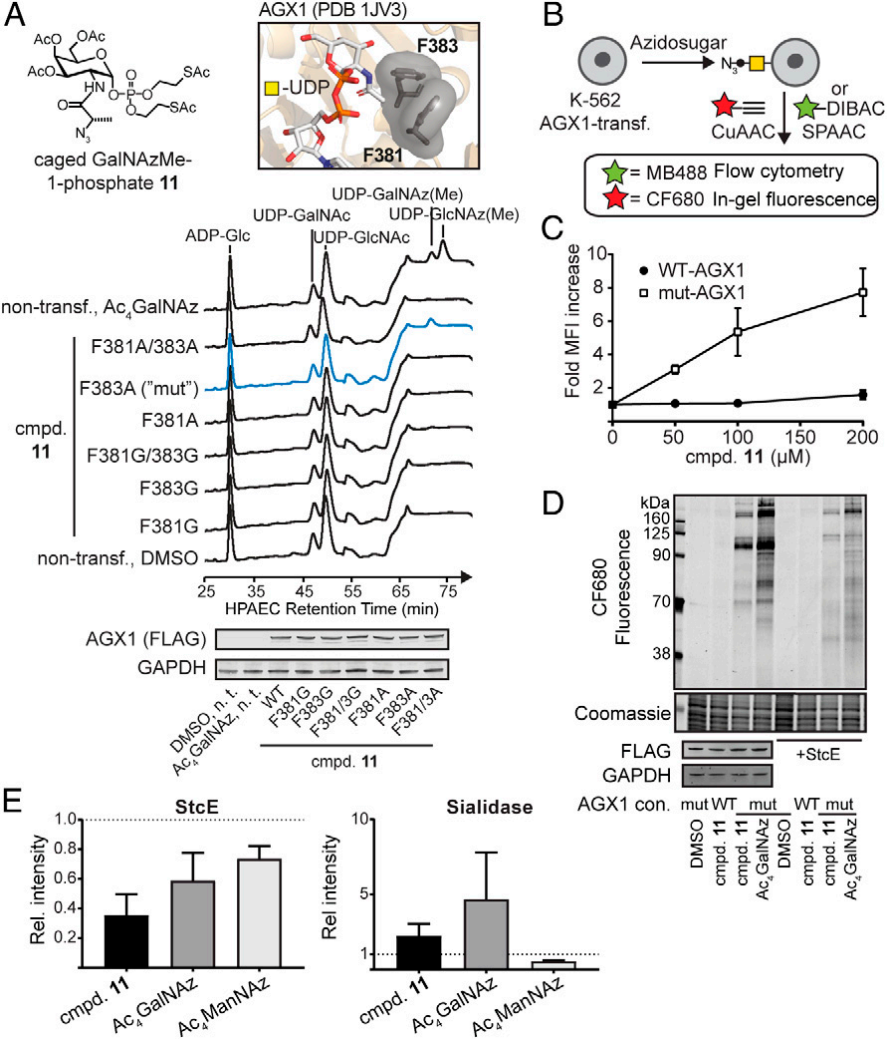
GalNAzMe can be used to label the cell surface glycoproteome. (*A*) Biosynthesis of UDP-GalNAzMe by mut-AGX1. HEK293T cells were transiently transfected with plasmids encoding for different AGX1 constructs or left nontransfected. Cells were fed with 200 μM compound **11** or Ac_4_GalNAz, and cell lysates were analyzed by HPAEC-PAD. *Inset*, active site of WT-AGX1. (*B*) Cell surface labeling workflow using either CuAAC or SPAAC. (*C*) Dose dependence of GalNAzMe labeling by K-562 cells stably expressing WT-AGX1 or mut-AGX1, as assessed by flow cytometry. Data are mean ± SD from three independent replicates. (*D*) Cell surface mucin labeling by GalNAzMe and GalNAz. K-562 cells stably expressing WT-AGX1 or mut-AGX1 were fed with DMSO, 3 μM Ac_4_GalNAz, or 100 μM compound **11** and treated with CF680-alkyne as outlined in *B*. Cells were optionally treated with 50 nM StcE before the click reaction. Data are from one representative of two independent experiments. (*E*) Cells were treated with either StcE or *Vibrio cholerae* sialidase and then treated with MB^TM ^488-DIBAC as outlined in *B*, and glycosylation was assessed by flow cytometry. Data are mean + SD of three independent experiments. GAPDH, glyceraldehyde-3-phosphate dehydrogenase; FLAG, DYKDDDDK epitope tag; n.t., non-transfected; PDB, protein database identifier.

We then opted to enable biosynthesis of UDP-GalNAzMe **5** in the living cell. Our initial attempts of using a per-acetylated precursor failed, as we did not observe **5** in cell lysates by high-performance anion exchange chromatography with pulsed amperometric detection (HPAEC-PAD). This was in line with previous findings on the low promiscuity of both endogenous biosynthetic enzymes GALK2 and AGX1 toward chemically modified substrate analogs (17, 26, 34). Mutants of AGX1 with enlarged active sites have been used by us and Yu et al. (35) to successfully transform analogs of GlcNAc-1-phosphate or GalNAc-1-phosphate into the corresponding UDP sugars and bypass the GALK2 phosphorylation step (Fig. 1*A*) (26). We thus synthesized a caged, membrane-permissive version of GalNAzMe-1-phosphate **11** (Fig. 2*A*) (26) and equipped cells with the capacity to biosynthesize UDP-GalNAzMe **5** (Fig. 1*A*). We transfected HEK293T cells with single and double mutants of the AGX1 active site residues Phe381 and Phe383 (Fig. 2*A*). Feeding these cells with caged GalNAzMe-1-phosphate **11** led to a peak corresponding to UDP-GalNAzMe **5** only when AGX1^F383A^ termed “mut-AGX1” (26) was present (Fig. 2*A* and *SI Appendix*, Fig. S2*B*). This result was somewhat surprising in the context of our previous finding that both AGX1^F383A^ and AGX1^F383G^ accepted a different chemically modified GalNAc-1-phosphate analog (26). When UDP-GalNAzMe **5** was biosynthesized from precursor **11**, we never observed a peak with the retention time of UDP-GlcNAzMe in two different cell lines (*SI Appendix*, Fig. S2*B*). As a control, Ac_4_GalNAz feeding generated an approximate 3:8 equilibrium between UDP-GalNAz and UDP-GlcNAz even without overexpression of AGX1 (Fig. 2*A*) (15). Collectively, these data indicate that UDP-GalNAzMe **5** can be biosynthesized by mut-AGX1 in living cells and is not epimerized by endogenous GALE.

We next assessed incorporation of GalNAzMe into cell surface glycans. K-562 cells stably transfected with WT- or mut-AGX1 were treated with caged GalNAzMe-1-phosphate **11**, Ac_4_GalNAz, or dimethyl sulfoxide (DMSO). Azide-containing glycans on the surface of living cells were reacted with clickable (by CuAAC or SPAAC) fluorophores and visualized by flow cytometry or in-gel fluorescence imaging (Fig. 2 *C*–*E*) (13, 14). Caged GalNAzMe-1-phosphate **11** exhibited dose- (Fig. 2*C*) and time-dependent (*SI Appendix*, Fig. S3*A*) incorporation when cells expressed mut-AGX1 but little incorporation in the presence of WT-AGX1. Our data confirmed that UDP-GalNAzMe **5** must be biosynthesized for fluorescent labeling to be detectable, thereby ruling out nonspecific incorporation (36).

To elucidate the nature of azide-labeled cell surface glycans, we compared the glycoprotein patterns labeled with GalNAz or GalNAzMe by in-gel fluorescence. Feeding caged GalNAzMe-1-phosphate **11** labeled a subset of the glycoprotein bands of Ac_4_GalNAz (Fig. 2*D*), consistent with UDP-GalNAz **2** being epimerized and entering GlcNAc-containing glycans. The same behavior was observed in HepG2 cells (*SI Appendix*, Fig. S3*B*). To assess labeling specificity, we also tested glycoprotein susceptibility toward hydrolytic enzymes. We treated samples with the mucinase StcE that specifically digests highly O-GalNAcylated mucin domains or with sialidase that removes sialic acid from glycoconjugates (37). Following StcE treatment, the most intense bands labeled by both caged GalNAzMe-1-phosphate **11** and Ac_4_GalNAz feeding had disappeared. The remaining band pattern was much more complex in samples from Ac_4_GalNAz- than from **11**-fed cells (Fig. 2*D*). Flow cytometry confirmed that StcE treatment decreased the overall labeling intensity of cells fed with caged GalNAzMe-1-phosphate **11**, Ac_4_GalNAz, or the azide-tagged sialic acid precursor Ac_4_ManNAz (Fig. 2*E*). In contrast, sialidase treatment led to an increase of labeling with both **11** and Ac_4_GalNAz, presumably due to better accessibility by the click reagents to the azide-tagged glycan structures without sialic acid. The labeling intensity after feeding Ac_4_ManNAz was reduced by sialidase treatment (Fig. 2*E* and *SI Appendix*, Fig. S3*C*). These data suggest that GalNAzMe enters the mucin subset of GalNAz-modified glycoproteins, and neither GalNAc derivative substantially enters the sialic acid pool. We further found that both Ac_4_GalNAz and GalNAzMe-1-phosphate **11** exhibited a similar small growth reduction when fed repeatedly to K-562 cells (*SI Appendix*, Fig. S3*D*).

While characterizing the activity of mut-AGX1 in living cells, we found that the biosynthesis of both UDP-GalNAz and UDP-GlcNAz from per-acetylated precursors was enhanced in the presence of mut-AGX1 over WT-AGX1, leading to a severalfold increase of cell surface labeling (*SI Appendix*, Fig. S4). In contrast, Ac_4_ManNAz-induced labeling was not affected, indicating that mut-AGX1 is a versatile enzyme to facilitate GlcNAc- and GalNAc-based metabolic labeling.

**Fig. 3.**
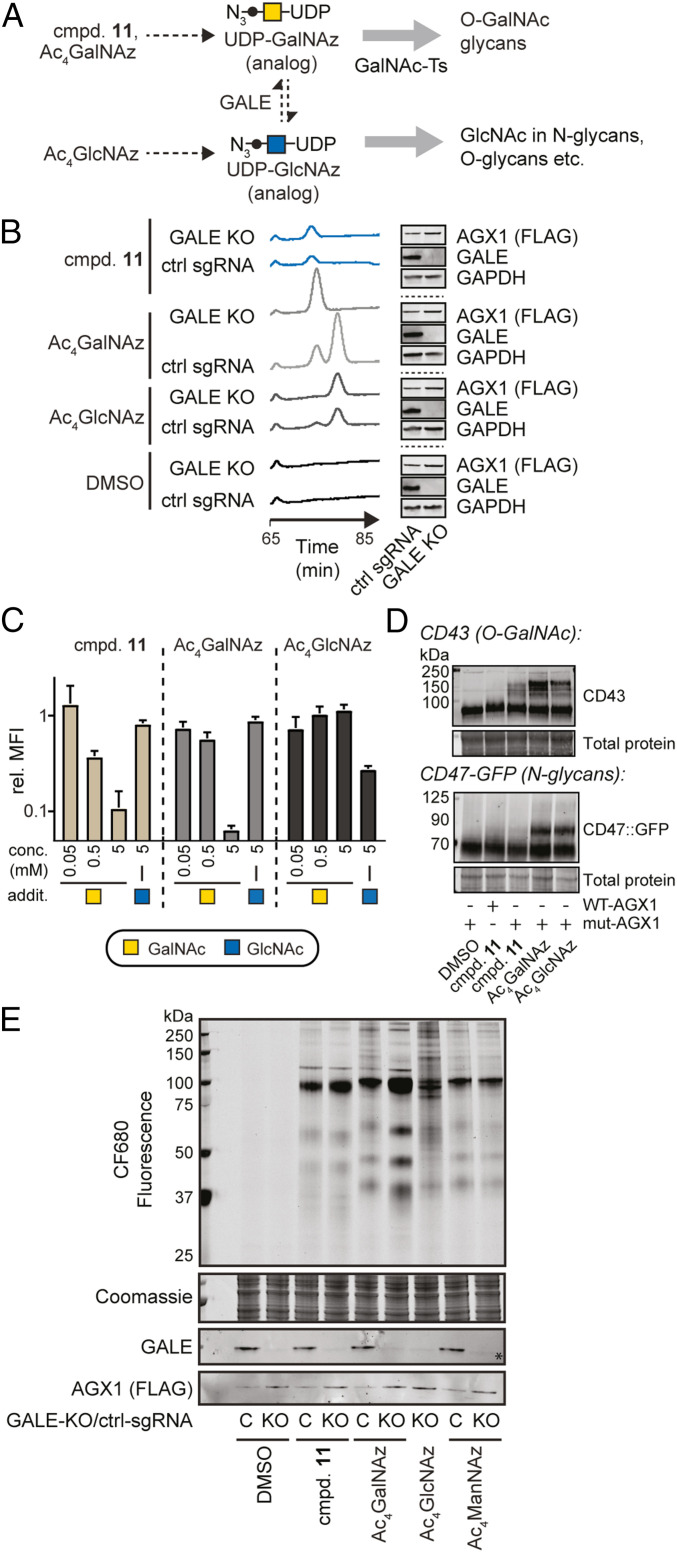
UDP-GalNAzMe is not epimerized and labeled a subset of the UDP-GalNAz–modified glycoproteome. (*A*) Schematic of the pathways probed herein. Both GalNAc-1-phosphate analog **11** and Ac_4_GalNAz are precursors for O-GalNAc glycosylation. (*B*) UDP-GalNAzMe is not epimerized in the living cell, while UDP-GalNAz and UDP-GlcNAz are epimerized. K-562 GALE-KO and control cells stably transfected with mut-AGX1 were treated with 200 μM compound **11**, Ac_4_GalNAz, DMSO, or Ac_4_GlcNAz, and UDP-sugar biosynthesis was assessed by HPAEC-PAD. (*C*) GALE-KO cells were treated with 100 μM compound **11**, 10 μM Ac_4_GalNAz, or 10 μM Ac_4_GlcNAz and supplemented with GalNAc or GlcNAc in the indicated concentrations. Cell surface labeling was assessed by flow cytometry after SPAAC using MB^TM ^488-DIBAC, and fluorescence intensity was normalized to DMSO-treated cells. Data are mean + SD from three independent experiments. (*D*) K-562 cells stably expressing WT- or mut-AGX1 were fed with DMSO, 100 µM compound **11**, 3 µM Ac_4_GalNAz, or 8 µM Ac_4_GlcNAz and subjected to PEG mass tagging. K-562 cells stably expressing WT- or mut-AGX1 and GFP::CD47 were fed with DMSO, 100 µM compound **11**, 3 µM Ac_4_GalNAz, or 8 µM Ac_4_GlcNAz and subjected to PEG mass tagging. (*E*) Cells were fed with compounds as in *D*, live cells were treated with CF680-alkyne under CuAAC conditions, and proteins in cell lysates were visualized by in-gel fluorescence. Ac_4_ManNAz (0.5 μM) was used as a positive control. GAPDH, glyceraldehyde-3-phosphate dehydrogenase; FLAG, DYKDDDDK epitope tag; MFI, mean fluorescence intensity; C, control-sgRNA.

We next confirmed that GalNAzMe specifically enters O-GalNAc glycosylation in living cells. We used mut-AGX1–transfected GALE-KO K-562 cells or the corresponding control cells carrying a noncoding sgRNA (26). In GALE-KO cells, GalNAz and GalNAzMe should enter the exact same subset of glycans. In cells expressing GALE, UDP-GalNAz **2** should be epimerized and label more cellular glycoproteins than UDP-GalNAzMe **5** (Fig. 3*A*). We first profiled UDP-sugar levels by HPAEC-PAD in azido sugar-fed cells.

As predicted, UDP-GalNAz **2** and UDP-GlcNAz (from the precursor Ac_4_GlcNAz) were not epimerized in GALE-KO cells while epimerization occurred in GALE-expressing cells (Fig. 3*B*) (26). UDP-GalNAzMe **5** levels were equal in both cell lines fed with **11**, and no epimerization was observed irrespective of the presence of GALE. To confirm that these azido sugars enter glycans, we performed a competition experiment in GALE-KO cells by flow cytometry. We used the free sugars GalNAc and GlcNAc to compete with metabolic labeling and SPAAC to fluorescently detect azide-containing glycoproteins (Fig. 3 *C* and *D*). Cells fed with both Ac_4_GalNAz and caged GalNAzMe-1-phosphate **11** lost fluorescence intensity in the presence of increasing concentrations of GalNAc, while only Ac_4_GlcNAz labeling was abrogated by an excess of GlcNAc (Fig. 3*C*).

We then assessed glycosylation of discrete bona fide O-GalNAc–glycosylated or *N*-glycosylated proteins with azido sugars. CD43, the most abundant cell surface glycoprotein on K-562 cells, is heavily O-GalNAc glycosylated (38). In contrast, CD47 contains six potential *N*-glycosylation sites and no predicted O-GalNAc glycans (39). We fed normal or CD47-GFP–overexpressing K-562 cells with caged GalNAzMe-1-phosphate **11**, Ac_4_GalNAz, Ac_4_GlcNAz, or DMSO. Cell lysis and subsequent conjugation with an azide-reactive 10-kDa polyethylene glycol (PEG) chain by SPAAC led to a mass shift visible by western blot whenever the azidosugar was incorporated (40, 41). We observed a clear mass shift in CD43 after feeding GalNAzMe-1-phosphate **11**, Ac_4_GalNAz, or Ac_4_GlcNAz to WT K-562 cells (Fig. 3*D*). The mass shift induced by GalNAzMe-1-phosphate **11** was only observed when mut-AGX1 was expressed. The Ac_4_GlcNAz-induced mass shift was lost in GALE-KO cells, confirming that these cells could not generate UDP-GalNAz from UDP-GlcNAz (*SI Appendix*, Fig. S5*A*). A mass shift in overexpressed CD47-GFP was only seen in lysates of cells fed with Ac_4_GalNAz or Ac_4_GlcNAz but not with caged GalNAzMe-1-phosphate **11** (Fig. 3*D*). CD43 was labeled by **11** in the same cell line (*SI Appendix*, Fig. S5*B*).

In-gel fluorescence confirmed that caged GalNAzMe-1-phosphate **11** and Ac_4_GalNAz led to identical band patterns of glycoproteins in GALE-KO cells (Fig. 3*E*). Strikingly, Ac_4_GlcNAz feeding of GALE-KO cells led to a diffuse pattern of low-intensity glycoprotein bands that resembled the background bands of WT cells fed with Ac_4_GalNAz. Furthermore, the GalNAzMe labeling pattern was not influenced by the presence or absence of GALE. Taken together, these data indicate that UDP-GalNAzMe **5** exclusively enters O-GalNAc glycans, while UDP-GalNAz **2** is epimerized and additionally enters GlcNAc-containing glycans. Notably, a 3- to 5-kDa difference in molecular mass was seen between proteins labeled with Ac_4_GalNAz in WT cells on one hand and either GALE-KO cells fed with Ac_4_GalNAz or any cell line fed with caged GalNAzMe-1-phosphate **11** on the other hand (Fig. 3*E*). We assume that the weight shift may be due to a difference in either glycan elaboration or glycosylation site occupancy and note that this labeling behavior is further validation that GalNAzMe in WT cells mimics the attributes of GalNAz in GALE-KO cells.

To further structurally confirm that UDP-GalNAzMe **5** is not accepted as a substrate by GALE but is accepted by GalNAc-Ts such as GalNAc-T2, we computationally docked UDP-GalNAzMe into the active sites of both enzymes. We found that the energy-minimized conformation would place the 2-azidopropioamide side chain closer (2.7 and 2.9 Å) than the N–C van der Waals radius of 3.3 Å from nearby amino acid side chains in GALE (*SI Appendix*, Fig. S5*C*). In contrast, UDP-GalNAzMe was accommodated in GalNAc-T2 without such steric clashes.

### GalNAzMe as an O-GalNAc–Specific Reporter Molecule.

**Fig. 4.**
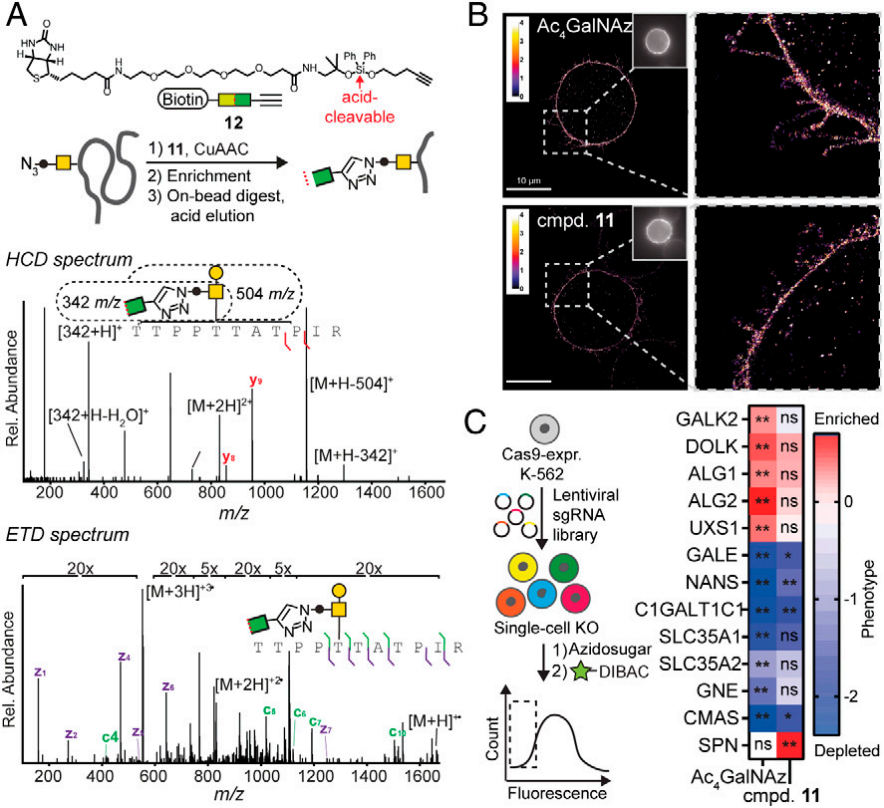
GalNAzMe is a reporter for the biology of O-GalNAc glycosylation. (*A*) GalNAzMe as a reporter in MS-based glycoproteomics of the HepG2 secretome. Exemplary mass spectra from GalNAzMe-containing glycopeptides. (*B*) GalNAzMe as a reporter for superresolution microscopy using K562 cells for labeling with GalNAzMe or GalNAz and CuAAC with Alexa Fluor 647 alkyne as a visualization strategy. (Scale bar, 10 µm.). *Insets*, whole cell images. (*C*) GalNAzMe as a reporter for a genome-wide CRISPR-KO screen in K-562 cells stably transduced with Cas9 and mut-AGX1 followed by feeding with Ac_4_GalNAz or compound **11**, labeled by MB^TM ^488-DIBAC, and subjected to FACS to sort the bottom 15% fluorescent cells and sequence sgRNAs. Effects on selected glycogenes are shown—color depicts the relative phenotype (positive/red: enriched in the low-fluorescence population; negative/blue: depleted in the low-fluorescence bottom population), while asterisks depict false discovery rate (FDR) as a measure of statistical significance from two independent experiments. ETD, electron-transfer dissociation; HCD, higher-energy collisional dissociation; ns, nonsignificant. *FDR 5%; **FDR 2%.

We obtained MS evidence for incorporation of GalNAzMe into O-GalNAc glycans. We first confirmed that global cell surface N- and O-glycome profiles of K-562 cells fed with either caged GalNAzMe-1-phosphate **11** or Ac_4_GalNAz did not differ substantially (*SI Appendix*, Fig. S6). We then used chemical MS glycoproteomics to assess the incorporation of GalNAzMe into cell surface O-GalNAc glycans. Biotin-containing, acid-cleavable alkynyl probe **12** served to enrich azide-containing glycoproteins from the de–N-glycosylated secretome of HepG2 cells (*SI Appendix*, Fig. S7*A*). Samples were digested with Lysyl endopeptidase (LysC) after enrichment on Lys-dimethylated Neutravidin beads with enhanced LysC resistance (42). Following glycopeptide release, tandem MS was used to sequence glycopeptides. Higher-energy collisional dissociation served to characterize glycan-derived ions, and spectra containing the ions for GalNAzMe (343.1617 mass-to-charge ratio [*m/z*]) and GalNAz (329.1461 *m/z*) triggered corresponding electron-transfer dissociation to sequence peptides (26). All spectra were manually validated. Both GalNAzMe and GalNAz were found as peptide-proximal residues in O-GalNAc glycans (Fig. 4*A*, Dataset S1, and *SI Appendix*, Fig. S7 *B* and *C*) and were extended by the downstream glycosylation machinery (43). For instance, biosynthetic considerations allowed the assignment of the disaccharide β-Gal-(1–3)-α-GalNAzMe-(Thr*) on the glycopeptide TTPPT*TATPIR of human fibronectin, along with other glycoforms and even a diglycosylated peptide TTPPT*T*ATPIR (Dataset S1). DMSO feeding did not lead to discernible signal. Taken together, GalNAzMe is a substitute of the peptide-proximal O-GalNAc residue.

We then probed the potential of GalNAzMe as an O-GalNAc–specific reporter molecule in methods of modern glycobiology. Superresolution microscopy was used to image the glycocalyx on mut-AGX1–transfected K-562 cells fed with caged GalNAzMe-1-phosphate **11** and Ac_4_GalNAz (Fig. 4*B*). Recently described mucin-covered tubules on these cells were clearly visible with both reagents, reflecting the fact that mucins are the most abundant glycoproteins in this cell line (44).

We next employed GalNAzMe-1-phosphate **11** as a reporter in a fluorescence-based genome-wide CRISPR-KO screen to investigate the genetic factors of glycan biosynthesis (Fig. 4*C* and Datasets S2 and S3). Specifically, we hypothesized that GalNAzMe labeling would be sensitive to knockout (KO) of genes that mediate cell surface O-glycan presentation, such as mucins. GalNAz labeling, conversely, is likely to be reduced by KO of a wider array of glycogenes. We thus conducted paired genome-wide KO screens to reveal, in an unbiased manner, the key genes that are essential for cell surface incorporation of the two metabolic labels. K-562 cells stably expressing *Streptococcus pyogenes* Cas9 and mut-AGX1 were transduced with a lentiviral plasmid library encoding 212,821 sgRNAs targeting 20,549 genes (10 sgRNAs per gene) (45). Cells were subsequently fed with caged GalNAzMe-1-phosphate **11** or Ac_4_GalNAz and treated with the fluorophore MB^TM ^488-Azadibenzylcyclooctyne (DIBAC) under SPAAC conditions. Cells with the 15% lowest fluorescence intensity were collected via fluorescence-activated cell sorting (FACS). Changes in sgRNA frequency were determined by deep sequencing and calculated relative to a nontreated control sample. Using the multiplicity of sgRNAs targeting the same gene, a statistical score and effect size could be derived for each gene using the Cas9 high-Throughput maximum Likelihood Estimator (casTLE) scoring system (46). The gene encoding for the GalNAc 1-kinase GALK2 was essential for labeling with Ac_4_GalNAz but not significant for labeling with caged GalNAzMe-1-phosphate **11** (Fig. 4*C* and *SI Appendix*, Fig. S7 *D* and *E*). This finding is consistent with the use of caged sugar-1-phosphates, such as **11**, to bypass the GALK2 step (26, 35). Strikingly, targeting the genes encoding for dolichol kinase DOLK and the mannosyltransferases ALG1 and ALG2 in the N-glycan biosynthesis pathway was detrimental for Ac_4_GalNAz labeling. In contrast, the same genes were not essential for labeling with caged GalNAzMe-1-phosphate **11**, consistent with our findings that GalNAzMe does not label N-glycans. KO of UDP-glucuronic acid decarboxylase (UXS1), an early enzyme in the biosynthesis of glycosaminoglycans such as heparin sulfate (HS), was also detrimental for GalNAz but not GalNAzMe labeling (Fig. 4*C* and *SI Appendix*, Fig. S7 *D* and *E*). UDP-GalNAz **1** may enter HS after epimerization to UDP-GlcNAz that can be used as a substrate by the HS polymerases EXT1/EXT2 (47). Conversely, one of the top genes associated with GalNAzMe signal was *SPN* encoding for CD43, consistent with CD43 being glycosylated with GalNAzMe (Fig. 3*D*). CD43 KO was not detrimental for GalNAz fluorescence, indicating that other glycans, including N-glycans, may compensate for the loss of CD43 under these conditions. Loss of several genes that encode for glycan biosynthetic determinants led to a net increase of fluorescence intensity. This was indicated by a depletion of sgRNAs targeting those genes in the sorted pool of 15% cells with lowest fluorescent labeling. These genes were generally associated with the elaboration of glycans that, upon loss, probably led to better accessibility of azido sugars to the click reagents. For instance, the chaperone C1GALTC1 is implicated in elaborating O-GalNAc glycans using UDP-galactose, a metabolite that is, in turn, shuttled into the Golgi compartment by the transporter SLC35A2. KO of both *C1GALTC1*and *SLC35A2* led to deenrichment in the low-labeling pool (Fig. 4*C*). Loss of GALE generally leads to a decrease of cellular UDP-GalNAc levels (26). As a consequence, azide-tagged UDP-GalNAc analogs might be preferentially used as substrates by GalNAc-Ts, explaining the concomitant increase in fluorescence labeling (26). Furthermore, impaired sialic acid biosynthesis by KO of the transporter SLC35A1 or the enzymes NANS, GNE, and CMAS led to an increase of labeling with both **11** and Ac_4_GalNAz. This finding is in line with our result that sialidase treatment of the cell surface increased the labeling intensity of a clickable fluorophore (*SI Appendix*, Fig. S3*C*). Taken together, these results validate GalNAzMe as a potent reporter tool for further genetic profiling of O-GalNAc glycan biosynthesis.

### BH-Mediated Increase of GalNAzMe Labeling by GalNAc-T2.

**Fig. 5.**
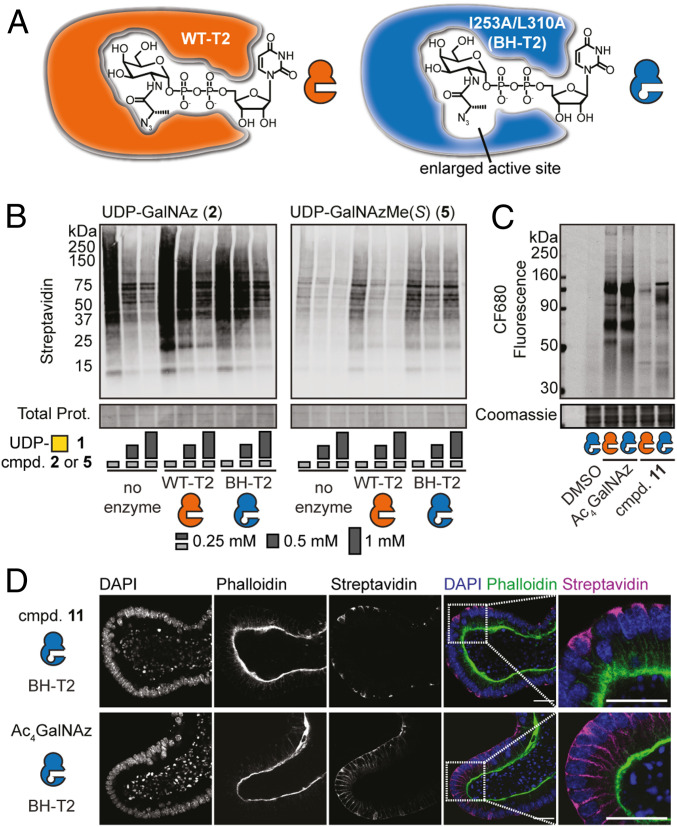
An engineered BH-T2 double mutant enhances GalNAzMe labeling. (*A*) The principle of BH engineering using engineered GalNAc-T2 (BH-T2) that preferentially accommodates UDP-GalNAzMe. (*B*) In vitro glycosylation using WT- or BH-T2 as enzyme sources. UDP-GalNAz **2** and UDP-GalNAzMe **5** were used as substrates, and UDP-GalNAc **1** was used as a competitor at different concentrations. Azide-labeled glycoproteins were visualized as in Fig. 2*B*. Data are from one representative of two independent replicates. (*C*) Live cell surface glycosylation by K-562 cells stably transfected with mut-AGX1 and WT- or BH-T2 and fed with DMSO, 50 μM compound **11**, or 3 μM Ac_4_GalNAz. Data are from one representative of two independent replicates. (*D*) Glycosylation in intestinal organoids transfected with mut-AGX1 and BH-T2. Organoids were fed with 50 µM compound **11** or 1.5 µM Ac_4_GalNAz, fixed, and treated with biotin alkyne under CuAAC conditions followed by streptavidin Alexa Fluor 647 staining. Data are from one representative of two independent experiments and shown as grayscale images for each channel and a color merge image of all three channels. (Scale bar, 100 µm.) *Insets*, magnifications. DAPI, 4′,6-diamidino-2-phenylindole.

Although UDP-GalNAzMe **5** can be biosynthesized by mut-AGX1 and enter O-GalNAc glycans, we consistently observed moderate glycoprotein labeling efficiency compared with UDP-GalNAz **2**. While it is not surprising that increasing specificity of a reagent impairs its efficiency, we tested whether GalNAzMe signal could be enhanced by a chemical genetics approach. One of the factors hampering signal was low acceptance by WT-GalNAc-Ts (*SI Appendix*, Fig. S2*A*). We therefore opted to develop a programmable labeling boost by making use of our BH-GalNAc-T technology (25, 26). We employed the GalNAc-T2^I253A/L310A^ double mutant (BH-T2) that exhibits a twofold increased activity with UDP-GalNAzMe **5** compared with the WT enzyme but displays lower activity with UDP-GalNAc **1** and UDP-GalNAz **2** (Fig. 5*A*) (25, 26). Labeling of membrane proteins with UDP-GalNAzMe **5** by WT-T2 in vitro was competed out by increasing concentrations of UDP-GalNAc **1** (Fig. 5*B*). In contrast, labeling with **5** by BH-T2 could not be competed out with UDP-GalNAc **1**. Labeling with UDP-GalNAz **2** was competed out by an excess of UDP-GalNAc **1** in the presence of both WT- and BH-T2. The presence of BH-T2 also led to a marked increase of glycoprotein labeling with caged GalNAzMe-1-phosphate **11** compared with WT-T2 in the living cell, as observed by in-gel fluorescence experiments (Fig. 5*C*). In contrast, Ac_4_GalNAz labeling was unchanged. These data indicate that O-GalNAc labeling by GalNAzMe can be enhanced by BH-engineered BH-T2.

### Labeling the O-GalNAc Glycome in Organoids.

We then turned to investigating O-GalNAc glycosylation in a multicellular model system. Intestinal organoids are instrumental in understanding some of the key concepts of bowel cancer formation as well as normal gut development and homeostasis (48–52). Production of O-GalNAc glycans in such systems is often probed by either backbone-directed antibodies or lectins (53, 54). We used GalNAzMe as an O-GalNAc glycan detection tool that is independent of both protein backbone and glycan capping but reports on the peptide-proximal, invariant GalNAc moiety. We stably transfected murine intestinal organoids with both mut-AGX1 and BH-T2 and fed either caged GalNAzMe-1-phosphate **11** or Ac_4_GalNAz (55). Treatment with a clickable biotin-alkyne under CuAAC conditions and fluorescently labeled streptavidin indicated a striking difference in labeling patterns between the two azido sugars by confocal microscopy (Fig. 5*D*). Ac_4_GalNAz labeling was generally found on all cell surfaces, including intercellular boundaries. In contrast, caged GalNAzMe-1-phosphate **11** labeling was focused on a subset of cells. Our labeling strategy was topologically restricted to the basolateral (nonluminal) side of the organoids, and GalNAzMe labeling was broadly localized to both cell surface and a subcortical space. Streptavidin signal was absent in both nontransfected, **11**-fed organoids as well as transfected, DMSO-fed organoids, excluding nonspecific labeling (*SI Appendix*, Fig. S8). We concluded that caged GalNAzMe-1-phosphate **11** is a valuable labeling tool with an O-GalNAc glycan precision that is not seen in the conventional reagent Ac_4_GalNAz.

## Discussion

Efforts to map the systems biology of organisms, tissues, and single cells demand specific and curated reporter tools. The capacity to accurately report on the presence and dynamics of individual glycan types is essential to understanding how glycans impact biological processes. Protein-based reporter reagents have enabled the study of glycobiology but rarely probe nonaccessible glycan core structures. Thus far, the forays made into developing chemical tools have yielded an arsenal of monosaccharide analogs: for instance, of ManNAc/Sia (8, 56–58), GlcNAc (18–20, 35), Fuc (59), Gal (60, 61), and GalNAc/GlcNAc (10, 15, 17, 62, 63). Probes are typically selected based on their labeling intensity, which in turn, is often a function of poor glycan specificity. The usefulness of these probes in biological applications is therefore limited, especially in the case of GalNAc analogs that can be epimerized to the corresponding UDP-GlcNAc analogs. UDP-GlcNAc is not only thermodynamically more stable than UDP-GalNAc but also used by a much more diverse set of glycosyltransferases (www.cazy.org) (15). The possibility to interconvert derivatives of both metabolites is therefore likely to create a GlcNAc-dependent labeling background if GalNAc is actually to be studied. Here, a panel of synthetic UDP-GalNAc analogs was essential to corroborate our structure-based design of a GalNAc-specific metabolic labeling reagent. GalNAzMe is a useful monosaccharide in a range of biological applications, showcased here by superresolution microscopy, chemical glycoproteomics, a genome-wide CRISPR-KO screen, and imaging of intestinal organoids. Our approach to study O-GalNAc glycosylation by metabolic labeling is compatible with the presence of GALE, which is of particular importance for advanced model systems such as organoids that would be difficult to establish in a GALE-KO background with predictable phenotypic perturbations (64). In line with a reciprocal relationship between probe specificity and sensitivity, caged GalNAzMe-1-phosphate **11** was associated with lower intensity in fluorescent labeling experiments when compared with the less specific reagent Ac_4_GalNAz. Our finding that GalNAzMe incorporation can be elevated by expressing a BH-engineered GalNAc-T double mutant is therefore valuable, especially for experiments in which high glycan labeling intensity is desired. While cells need to be transfected to incorporate GalNAzMe, this provides an additional control for background labeling in the absence of mut-AGX1 and/or BH-GalNAc-Ts. Further, the use of transposase-mediated transfection of both AGX1 and GalNAc-T from the same vector renders our approach amenable to a wide range of cell lines and even complex model systems such as intestinal organoids. GalNAzMe is a precision tool that will prove to be valuable in tackling important mucin-specific biological questions.

## Materials and Methods

Experimental details on expression and purification of GALE, in vitro epimerization, peptide glycosylation, plasmids, cell transfection, lysate labeling, analysis of nucleotide-sugar biosynthesis, metabolic cell surface labeling, growth assessment, flow cytometry and in-gel fluorescence, superresolution microscopy, PEG mass tagging, genome-wide CRISPR-KO screen, click and enrichment of HepG2 secretome glycoproteins, organoid culture and generation of stably overexpressing organoid lines, organoid labeling and immunofluorescence, and chemical synthesis can be found in *SI Appendix*. The MS proteomics data have been deposited in the ProteomeXchange Consortium via the Proteomics Identification Database (PRIDE) partner repository with the dataset identifier PXD020648 (65).

## Supplementary Material

Supplementary File

Supplementary File

Supplementary File

Supplementary File

## Data Availability

Glycoproteomics data have been deposited in PRIDE Proteomics database (dataset identifier no. PXD020648) and are accessible under https://www.ebi.ac.uk/pride/archive/projects/PXD020648.
