# Supplementary material for: Metabolic precision labeling enables selective probing of O-linked N-acetylgalactosamine glycosylation
Source: Proc Natl Acad Sci U S A. 2020 Sep 28;117(41):25293–301. doi: 10.1073/pnas.2007297117 (PMC7568240; doi:10.1073/pnas.2007297117)
Supplement: Supplementary File [file pnas.2007297117.sapp.pdf]

PNAS
www.pnas.org

Supplementary Information for

Metabolic precision labeling enables selective probing of O-linked *N*-acetylgalactosamine glycosylation

Marjoke F. Debets*, Omur Y. Tastan*, Simon P. Wisnovsky, Stacy A. Malaker, Nikolaos Angelis, Leonhard K. R. Moeckl, Junwon Choi, Helen Flynn, Lauren J. S. Wagner, Ganka Bineva-Todd, Aristotelis Antonopoulos, Anna Cioce, William M. Browne, Zhen Li, David C. Briggs, Holly L. Douglas, Gaelen T. Hess, Anthony J. Agbay, Chloe Roustan, Svend Kjaer, Stuart M. Haslam, Ambrosius P. Snijders, Michael C. Bassik, W. E. Moerner, Vivian S. W. Li, Carolyn R. Bertozzi, Benjamin Schumann

Corresponding author: Benjamin Schumann
Email: b.schumann@imperial.ac.uk

This PDF file includes:

Figures S1 to S8 (pp. 2-9)
Legends for Datasets S1 to S3 (p. 10)
Supplementary Methods and Materials (pp. 11-20)
Compound characterization (pp. 20-39)
SI References (pp. 40-41)

Other supplementary materials for this manuscript include the following:

Datasets S1 to S3

www.pnas.org/cgi/doi/10.1073/pnas.2007297117

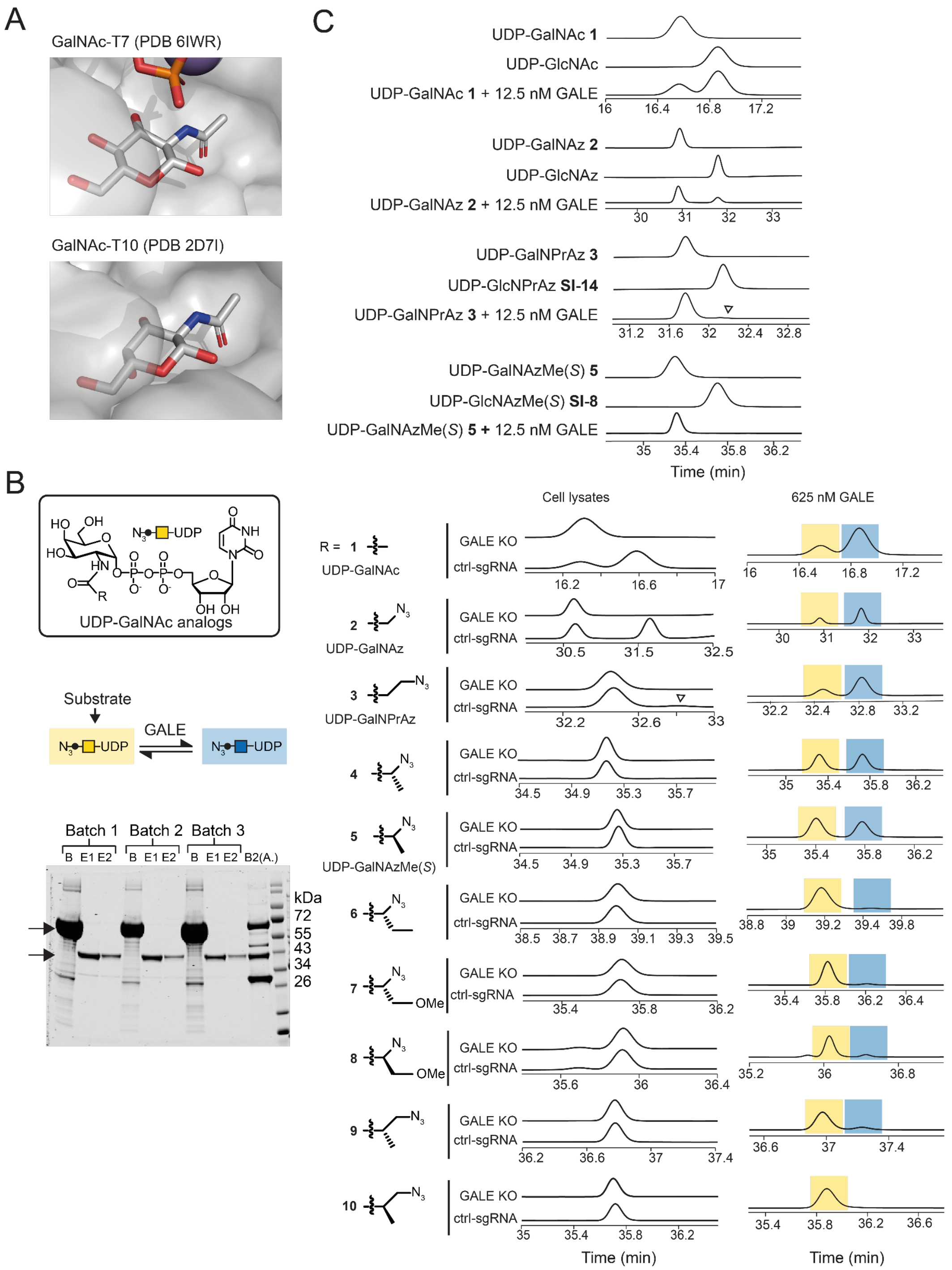

Fig. S1: Determinants of UDP-GalNAc analog acceptance by GalNAc-Ts and GALE. *A*, Modeling of GalNAc binding by GalNAc-T7 and T10. *B*, Ion pairs HPLC traces of *in vitro* glycosylation assays, using UDP-GalNAc analogs as substrates and either cell lysates from control or GALE-KO K-562 cells or a high concentration (625 nM) of purified GALE, shown in insert (arrows pointing to GALE before and after elution), as enzyme sources. Data are representative of three independent replicates (lysate samples) or from one experiment (625 nM GALE samples). *C*, selected traces from Fig. 1B using 12.5 nM GALE as an enzyme source, with reference HPLC traces of synthetic standards for UDP-GalNAc and UDP-GlcNAc analogs. Arrowhead depicts epimerization of compound **3**. Traces depict relative intensity of absorbance at 260 nm.

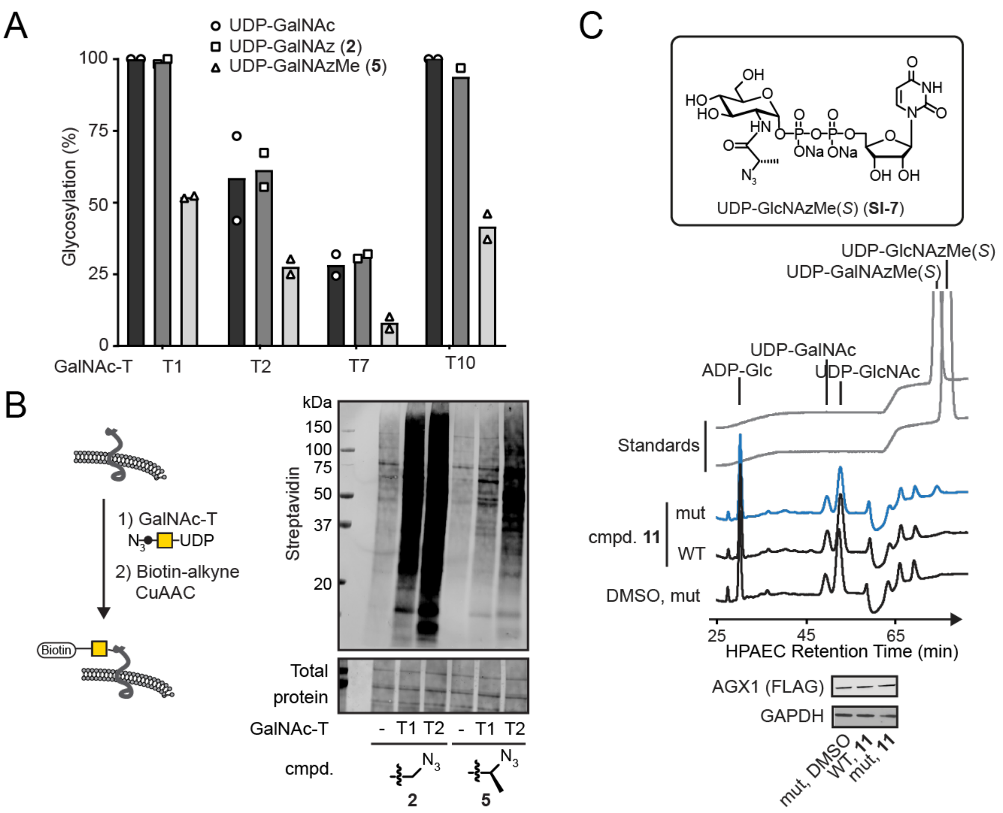

Fig. S2: UDP-GalNAzMe 5 recognition by GalNAc-Ts and delivery to the living cell. *A*, *in vitro* peptide glycosylation by purified GalNAc-Ts. Data are biological duplicates as average of technical duplicates. *B*, lysate protein glycosylation by GalNAc-T1 and GalNAc-T2. A membrane preparation was used as a lysate protein source, probed with soluble GalNAc-Ts and azide-tagged UDP-sugars, and subjected to CuAAC with clickable biotin. Streptavidin blot was used to visualize glycosylation. Data are from one representative out of three independent experiments. *C,* biosynthesis of UDP-GalNAzMe in K-562 cells stably transfected with WT- or mut-AGX1, as assessed by HPAEC-PAD. Standards include UDP-GalNAzMe (**5**) and its C4-epimer UDP-GlcNAzMe (**SI-7**).

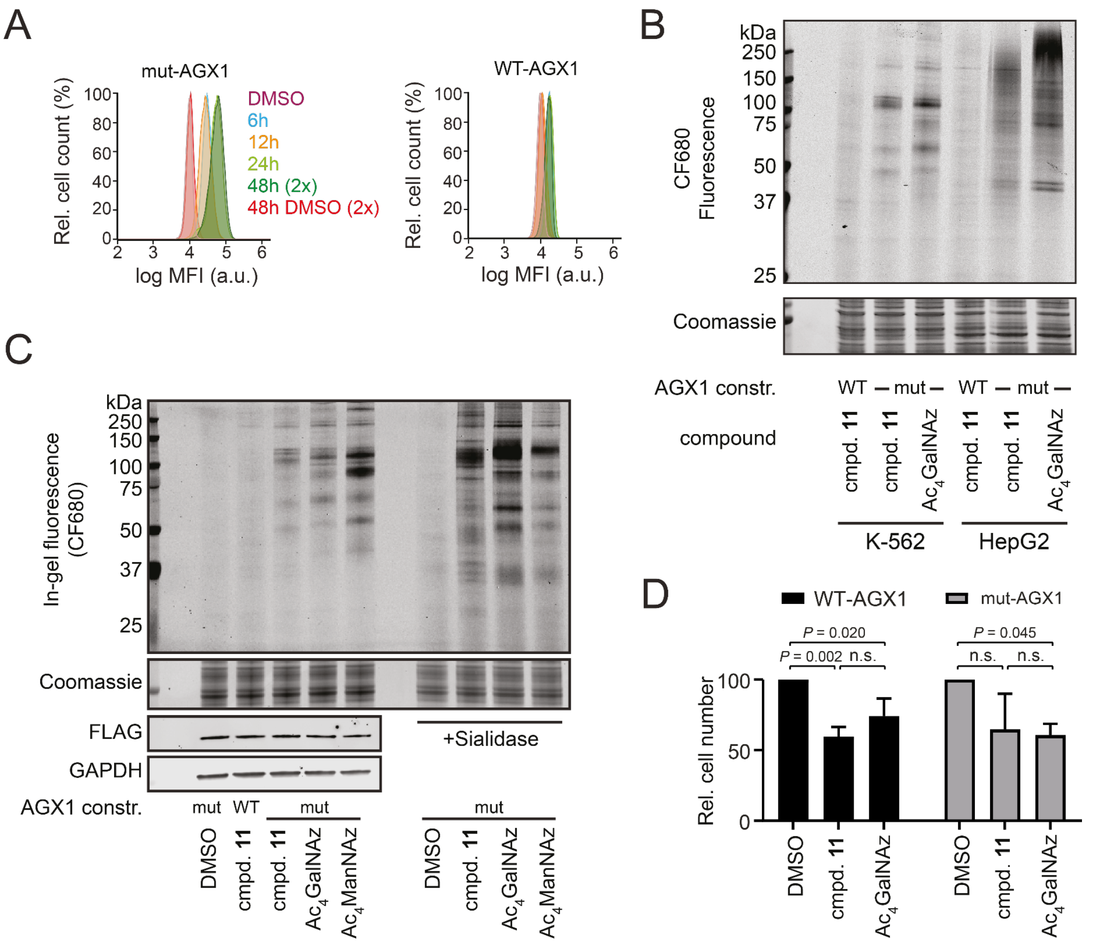

Fig. S3: Live cell labeling using caged GalNAzMe-1-phosphate 11. *A*, time-course of cell surface labeling with 100 µM compound **11**, as assessed by flow cytometry. "2x" denominates feeding for a total of 48 h, with feeding at 0 h and 24 h. *B*, comparison of labeling by K-562 and HepG2 cells stably expressing WT-AGX1 or mut-AGX1, as assessed by in-gel fluorescence. Data are from at least five (K-562) or one (HepG2) independent experiments. *C*, cell surface glycoprotein labeling by GalNAzMe, GalNAz, and ManNAz. K-562 cells stably expressing WT-AGX1 or mut-AGX1 were fed with DMSO, 3 µM Ac_4GalNAz, 100 µM compound **11**, or 1.5 µM Ac_4ManNAz and treated with CF680-alkyne under CuAAC conditions. Cells were optionally treated with 10 nM *Vibrio cholerae* sialidase before the click reaction. Data are from one experiment. *D*, effect of Ac4GalNAz or GalNAzMe-1-phosphate analog **11** feeding on cell growth. K-562 cells were fed twice with 100 µM compounds or DMSO over 48 h, and counted. Data are means + SD from three independent experiments. *P* values denote statistical evaluation by one-way ANOVA with Tukey's correction.

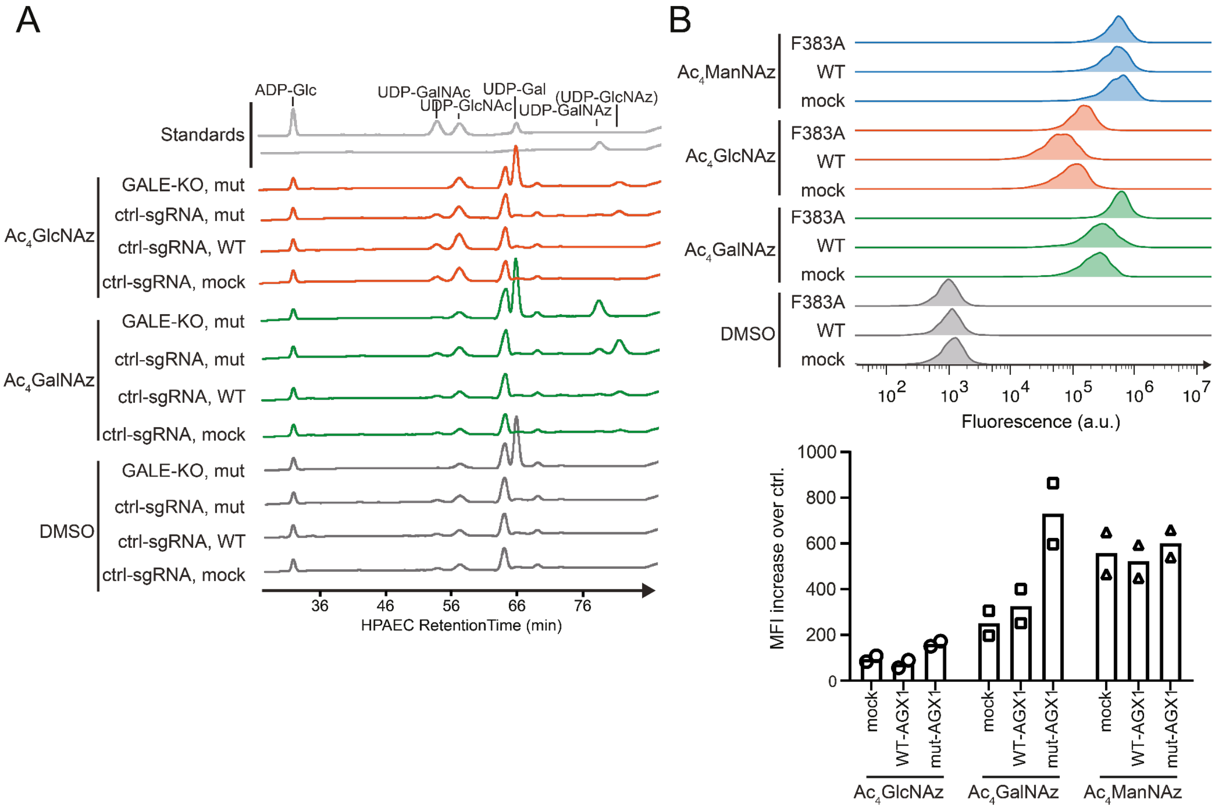

Fig. S4: Enhancing Ac4GalNAz and Ac4GlcNAz labeling by mut-AGX1 *A*, biosynthesis of UDP-GalNAz and UDP-GlcNAz in K-562 cells stably transfected with empty plasmid, WT- or mut-AGX1, as assessed by HPAEC-PAD. Data are from one representative out of two independent experiments. *B*, metabolic cell surface labeling of K-562 cells transfected with empty plasmid, WT-AGX1 or mut-AGX1 and fed with 10 µM Ac4GalNAz, 50 µM Ac4GlcNAz, 10 µM Ac4ManNAz, or DMSO, as assessed by flow cytometry, individual histograms and data from two independent experiments, normalized to control signal of DMSO-fed cells. MFI, median fluorescence intensity.

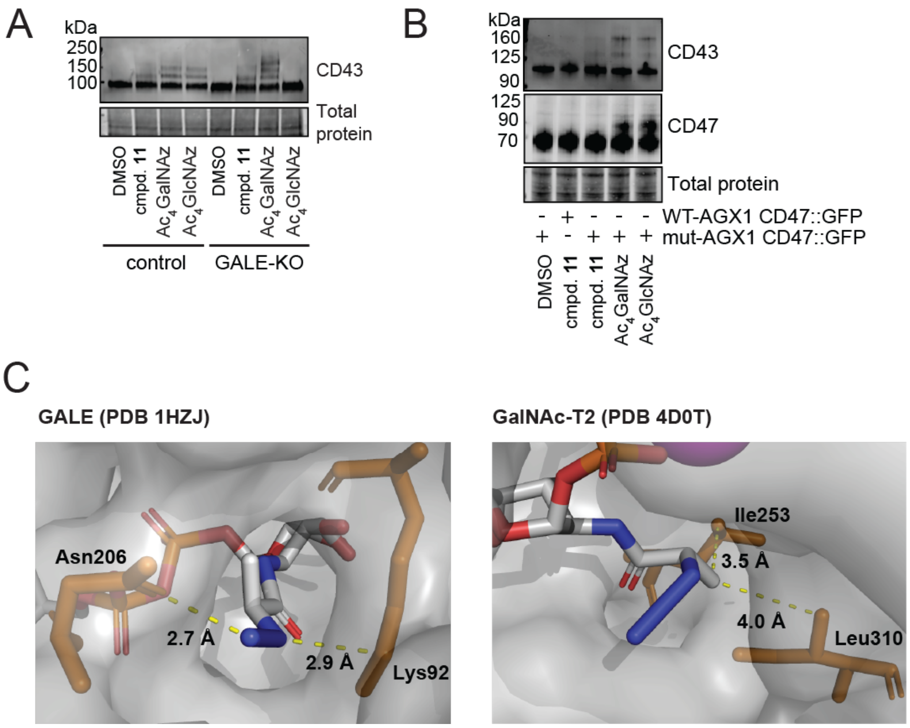

Fig. S5: Specific O-GalNAc labeling by GalNAzMe. *A*, K-562 GALE-KO or control cells were treated with DMSO, 100 µM compound **11**, 3 µM Ac_4GalNAz, or 8 µM Ac_4GlcNAz, and cell lysates were treated with a clickable 10 kDa PEG mass tag under SPAAC conditions. *B*, K-562 cells stably expressing WT- or mut-AGX1 GFP::CD47 were fed and subjected to PEG mass tagging as in *A* (replicate of Fig. 3D). Samples from the same SPAAC reaction were run side by side for detection of CD43 and CD47. *C*, docking of UDP-GalNAzMe into the active sites of GALE and GalNAc-T2. Distances shown are the closest interactions to amino acid residues in the active site after energy minimization.

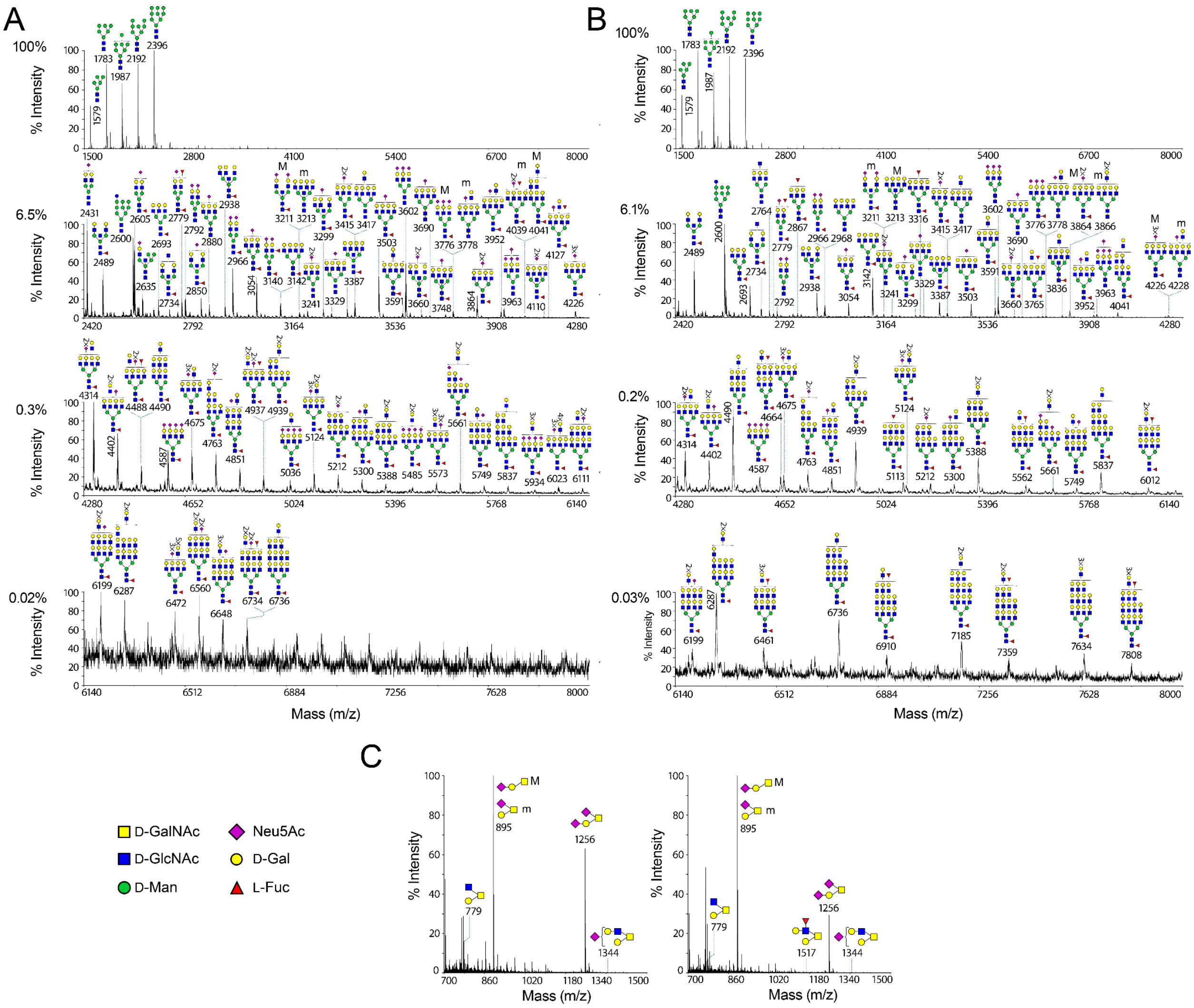

Fig. S6: The glycomes of cells treated with Ac4GalNAz and compound 11 are not substantially altered. *A, B*, MALDI-TOF mass spectra of permethylated N-glycans from lysates of K-562 cells treated with 3 μM $Ac_4GalNAz$ (*A*) or 100 μM compound **11** (*B*). *C*, O-glycomes from the same lysates as in *A* and *B* from cells treated with $Ac_4GalNAz$ (left panel) and compound **11** (right panel). Structures outside a bracket have not been unequivocally defined. “M” and “m” designations indicate major and minor abundances, respectively. Top panels in (*A*, *B*) depict the full spectra (*m/z* 1500-8000), while lower panels *(C)* depict partial MALDI-TOF MS spectra of the corresponding areas. Percentages in (*A*, *B*) on the left of each partial MALDI-TOF MS panel correspond to the relative intensity of the corresponding panel relative to the full spectrum (top panel). Putative structures are based on molecular ion composition, tandem MS/MS, and knowledge of biosynthetic pathways. All molecular ions are $[M+Na]^+$.

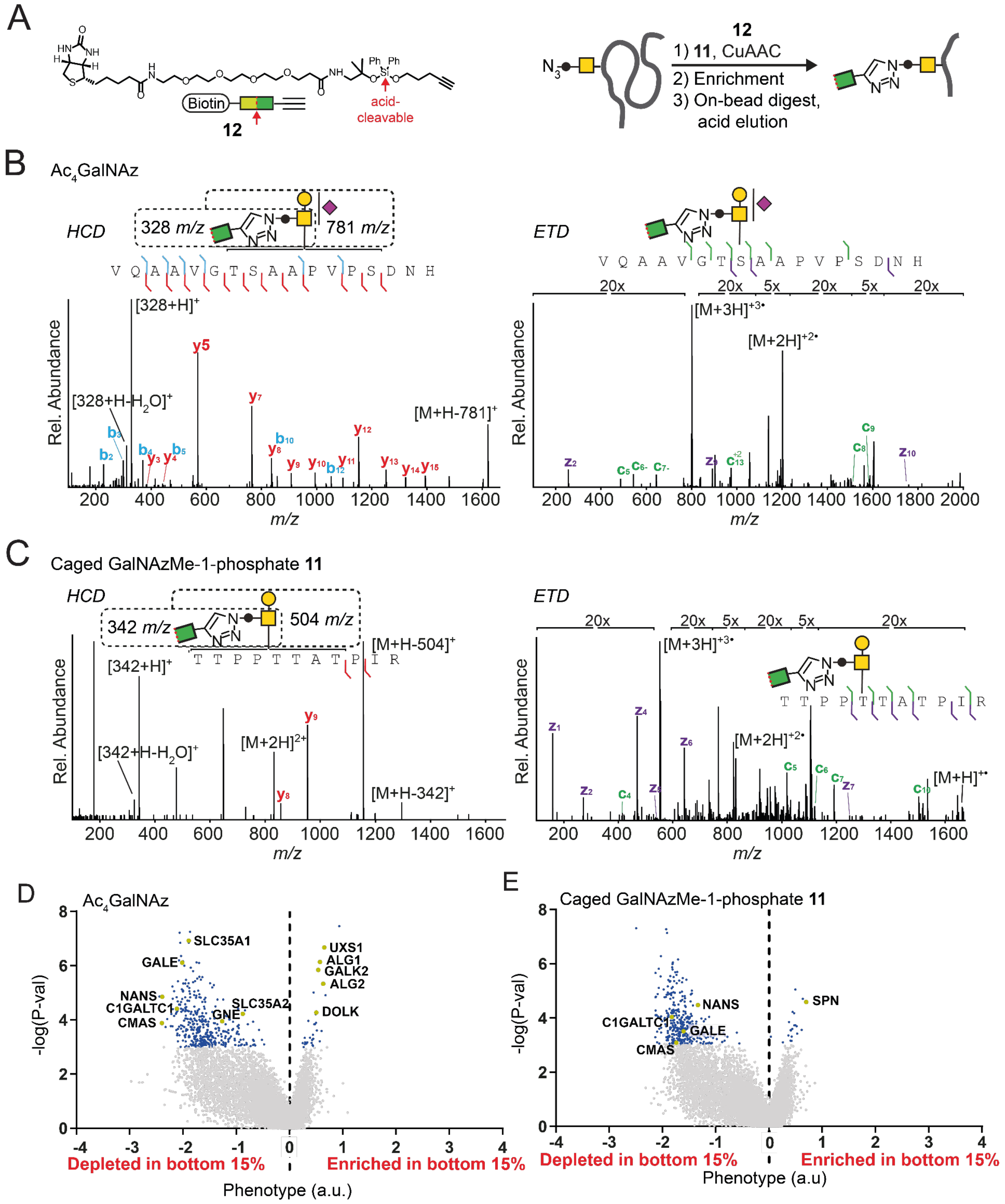

Fig. S7: GalNAzMe as a reporter molecule in glycoproteomics and a genome-wide CRISPR KO screen. *A*, MS glycoproteomics workflow using DADPS Biotin Alkyne **12**. *B*, exemplary mass spectra from GalNAz- and *C*, GalNAzMe-containing glycopeptides. *D* and *E*, Volcano plots of a genome-wide CRISPR-KO screen of K-562 cells treated as outlined in Fig. 4C. Genes with phenotypes (5% FDR) in the respective screens are highlighted in blue, and relevant genes shown in Fig. 4C are highlighted in yellow and annotated.

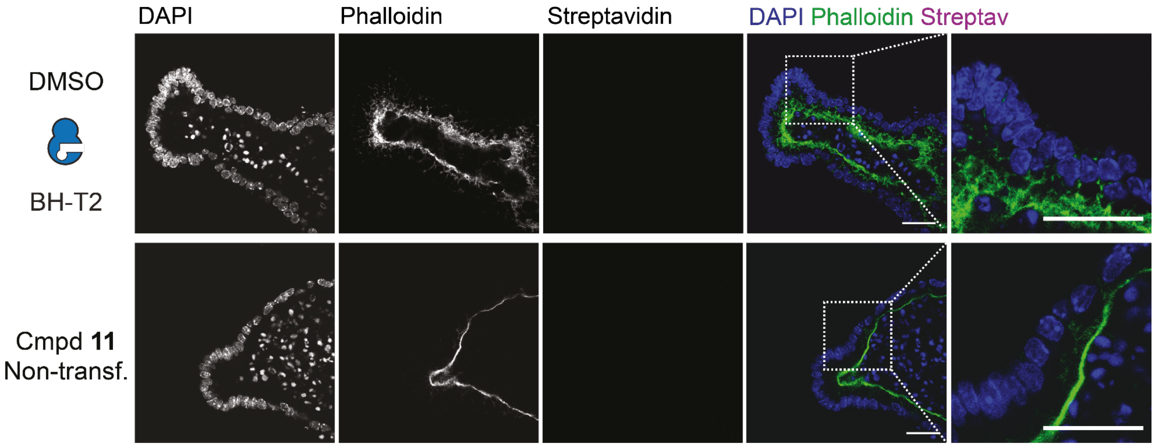

Fig. S8: Glycosylation in intestinal organoids transfected with mut-AGX1 and BH-GalNAc-T2 or non-transfected, control samples. Organoids were fed with DMSO (top) or 50 μM compound **11** (bottom), fixed and treated with biotin alkyne under CuAAC conditions followed by Streptavidin Alexa Fluor 647 staining. Data are from one representative out of two independent experiments and shown as grayscale images for each channel and a color merge image of all three channels. Scale bar, 100 μm.

Dataset S1 (separate file): Annotated glycopeptides from HepG2 secretome modified with GalNAz or GalNAzMe. Data are depicted according to the elution conditions (0.1% and 2% formic acid in water).

Dataset S2 (separate file): Genome-wide CRISPR KO screen on Ac_4GalNAz-fed cells. Data are processed from two independent replicates. Gene list, Phenotype, *P* values based on Mann-Whitney U test (MWU) and false-discovery rate (fdr) are provided according to a published procedure (24).

Dataset S3 (separate file): Genome-wide CRISPR KO screen on caged GalNAzMe-1-phosphate **11**-fed cells. Data are processed from two independent replicates. Gene list, Phenotype, *P* values based on Mann-Whitney U test (MWU) and false-discovery rate (fdr) are provided according to a published procedure (24).

Supplementary Methods and Materials

Expression and purification of human GALE from insect cells

The coding sequence of human GALE in pDONR221 (Clone ID HsCD00040708) was from DNASU (1–3). The coding sequence was cloned into pTriEx6-His-GST-3C-MCS (an in-house construct modified from pTriEx-6, Merck, Darmstadt, Germany) using the primers CCCTAAGCTTGGATCCAATGGCAGAGAAGGTGCTGG and GCTCGGTACCAGATCTCTAGGCTTGCGTGCCAAAG with a BamHI/BGI11 cloning strategy using the In-Fusion HD Cloning Kit (Takara, Kusatsu, Japan). Recombinant baculovirus was generated based on the *flash*BAC™ system (Oxford Expression Technologies, Oxford, UK). Sf21 cells were transfected with 0.5 µg of transfer plasmid and 100 ng of *flash*BAC™ DNA using Fugene HD (Promega, Madison, USA) according to manufacturer's instructions. After incubation overnight, 1 mL growth media with fungizone (1:1000, Thermo Fisher, Waltham, USA) was added and cells were incubated at 125 rpm, 27 °C for 5 days. Success of transfection and infection was judged by change in cell diameter and growth. To amplify the amount of viral stock (P1 to P2); 30 mL of Sf21 cells ($9x10^5$ cells/mL) were seeded at 6-well plates, incubated overnight and transferred to 30 mL Sf21 cell suspension for incubation at 125 rpm, 27 °C for 3 days. Supernatant was then collected (2000 *x g*, 5 min, 4 °C). Fetal bovine serum (FBS) was added to a concentration of 2% (v/v) to the filtered supernatant (0.22 µm filter). Viral supernatant (P2) was stored at 4 °C until required. For a final amplification of viral stock (P2 to P3); 100 mL of insect cells ($9x10^5$ cells/mL) incubated overnight and 100 µL of P2 was added and cells incubated at 125 rpm, 27 °C for 3 days. Supernatant was collected, purified and stored as described above (P3). The virus multiplicity of infection (MOI) was determined by qPCR.

GALE was expressed first by seeding 0.5 L Sf21 cells ($9x10^5$ cells/mL) and incubating at 27 °C. The following day, cells were infected with viral stocks (P3) using a MOI of 2. After incubation for 3 days, cells were harvested (2000 *x g*, 5 min, 4 °C) and stored at -80 °C. Pellets were thawed at room temperature and resuspended in 50 mL GALE Lysis Buffer (50 mM HEPES-KOH (pH 7.5), 150 mM NaCl, 1 mM EDTA, 1 mM DTT) with cOmplete protease inhibitors (Roche, Penzberg, Germany) and BaseMuncher mix (1:10,000, Expedeon, Cambridge, UK), and left at 4 °C for 1 h. Cells were then lysed by sonication using a Sonifier 450 (Branson, Hampton, USA) prior to ultra-centrifugation (108472 g, 30 min). The supernatant was collected and incubated overnight with 0.5 mL pre-equilibrated GST-4B Sepharose beads (Sigma Aldrich, St. Louis, USA) in ice-cold GALE Lysis Buffer containing 10% (v/v) glycerol. The supernatant was then collected (FT) (2000 *x g*, 3 min, 4 °C). The beads were washed twice with 10 mL GALE Lysis Buffer containing 10% (v/v) glycerol. An aliquot of 50 µL HRV 3C protease (produced in-house) and 2 mL of GALE Lysis Buffer containing 10% (v/v) glycerol was added before incubating at 4 °C for 2 h. Another 50 µL of protease were added, the incubation step was repeated and the supernatant collected (E1). The beads were further washed with 2 mL GALE Lysis Buffer containing 10% (v/v) glycerol and the supernatant collected (E2). Beads were then incubated overnight with 150 µL of HRV 3C protease and 2 mL GALE Lysis Buffer containing 10% (v/v) glycerol at 4 °C. The supernatant was collected (E3) and the beads washed twice with 2 mL GALE Lysis Buffer containing 10% (v/v) glycerol (E4, E5). E1-E5 were pooled and concentrated to 1 mL using a Vivaspin6 30K centrifugal tube (Sartorius, Göttingen, Germany). The concentrated sample was injected onto an ÄKTA™ Pure system, running a Superdex™ S75 16/60 gel filtration column (GE Life Sciences, Marlborough, USA), collecting 1 mL fractions in Gel Filtration Buffer (25 mM HEPES pH 7.4, 500 mM NaCl). Fractions were pooled, aliquoted (15.15 µM concentration) and stored at -80 °C.

In vitro epimerization

The protocol for *in vitro* epimerization was based on Kingsley et al. (4). K-562 GALE-KO and the corresponding control cells carrying a non-targeting sgRNA were prepared previously (5) and grown using RPMI with 10% (v/v) FBS, penicillin (100 U/mL), streptomycin (100 µg/mL), 20 µM galactose and 200 µM *N*-acetylgalactosamine. Five million cells were harvested (500 *x g*, 5 min, 4 °C), washed with PBS once, harvested and frozen at -80°C. Cells were treated with 250 µL 100 mM glycine-HCl, pH 8.7, and subjected to three freeze/thaw cycles between dry ice and room temperature (5 min each). Cell debris was harvested (12000 *x g*, 15 min, 4 °C), the supernatant

was transferred to a fresh tube and treated with 35 µL 80% (v/v) glycerol. Samples were aliquoted and stored at -80 °C. The protein concentration was between 6 µg/µL and 9 µg/µL.
In vitro epimerization reactions were run in 25 µL reactions, containing either cell lysates (12 µg protein) or purified GALE (12.5 or 625 nM) in 25 mM Glycine-HCl (pH 8.7), 200 µM NAD and 250 µM UDP-GalNAc analog. Reactions run with cell lysates additionally contained 5 mM sodium pyruvate. Reactions were run for 30 min (with purified GALE) or overnight (with cell lysates) at 37 °C, diluted with 75 µL water and cooled to 4 °C. Samples were run on a 1260 HPLC with diode array detector using a Poroshell 120, EC-C18, 2.7 µm, 3.0 x 150 mm column (Agilent, Santa Clara, USA). Solvents were: A = 100 mM potassium phosphate, pH 6.4, 8 mM tetrabutylammonium bisulfate; B = 80% A, 20% acetonitrile. Gradients were either 0 min 0% B; 30 min 40% B; 32 min 100% B; 34 min 0% B; 44 min 0% B, or 0 min 0% B; 19 min 25% B; 32 min 100% B; 34 min 100% B; 35 min 0% B; 46 min 0% B.
Samples were also run on an ICS-6000 with a quaternary pump and pulsed amperometric detection (Thermo Fisher) on a CarboPac PA1 4x250 mm column and a 4x50 mm guard column. Solvents were: A = 1 mM NaOH in degassed water; B = 1 mM NaOH, 1M NaOAc in degassed water. 0 min 60% B; 40 min 100% B; 45 min 100% B; 60 min 100% B.
Commercial or synthetic standards (200-500 µM) were used as controls.

Peptide glycosylation
HPLC was performed on a 1100 series HPLC system (Agilent). LC-MS experiments were carried out using a 1260 Infinity HPLC attached to a 6120 Quadrupole mass spectrometer (Agilent). Poroshell 120 EC-C18, 2.7 µm, 4.6 × 50 mm analytical LC columns (Agilent) were used for both HPLC and LC-MS.
In vitro glycosylation was performed according to our published protocol. Soluble GalNAc-T1, T2 and T10 enzymes were expressed according to a published procedure (6). Soluble GalNAc-T7 was expressed as a fusion construct with superfolder GFP in pGEn2-DEST (a kind gift from Kelley Moremen, University of Georgia, Athens, USA) in HEK293-F cells according to the manufacturer's instructions (Thermo) (7). Briefly, a 30 mL culture was transfected with 293Fectin (Thermo) according to the manufacturer's instructions. After 48 h, cells were harvested (500 g, 5 min, 4 °C) and supernatant was kept for protein isolation. The supernatant was centrifuged at (9000 g, 20 min, 4 °C), and a cOmplete protease inhibitor tablet (Roche, Basel, Switzerland) was added. Ni-NTA agarose (1 mL settled resin, Thermo) were washed with water and Phosphate Buffered Saline without Ca^{2+} or Mg^{2+} (PBS) containing 10 mM imidazole (pH 7.4), and added to the protein solution. The suspension was incubated for 1 h at 4 °C under rotation and poured into an empty polystyrene column (Bio-Rad, Hercules, USA). The resin was washed with 50 mL PBS containing 20 mM imidazole (pH 7.4), and elution was carried out with 10 mL PBS containing 250 mM imidazole (pH 7.4). GalNAc-T7 was concentrated with by centrifuge filtration (10 kDa MWCO, Millipore, Burlington, USA), washed with 25 mM Tris-Cl (pH 7.4), 150 mM NaCl and concentrated to approx. 1500 nM enzyme. Glycerol was added to a final concentration of 20% (v/v), and enzyme was frozen at -80 °C. Typically, 100-200 µg enzyme were obtained from a 30 mL culture.
For T2, T7 and T10, chromophore-containing, isoenzyme-optimized peptide substrates were used with an HPLC-based assay to assess conversion (6). For T1, EA2 peptide (Anaspec, Fremont, USA) was used as substrate with an LCMS-based assay to assess conversion, as azide-containing glycopeptides were not separable from the corresponding peptide substrate when a previously reported T1-optimized peptide was used (6).

All reaction mixtures contained 20.8 mM Tris-HCl (pH 7.4), 50 mM NaCl, 10 mM $MnCl_2$, 12.5% glycerol, GalNAc-T enzymes (80 nM T1, 25 nM T2, 50 nM T7, 60 nM T10), 250 µM UDP-sugar and 50 µM peptide substrate (EA2 for T1, (daa)GAGAPGPTPGPAGAGK for T2, (daa)GTT*PSPVPTTSTTSAP for T7 and T10; daa = 2,4-dinitrophenyl-5-L-alanine amide; T* = α-D-GalNAc-*O*-Thr) in 50 µL final volume. Reactions were carried out for 1 h (T2, T7, T10) or 2 h (T1), quenched with 150 mM EDTA pH 8.0 (25 µL) and analyzed by HPLC or LCMS (gradients as % acetonitrile in water with 0.1% (v/v) formic acid) (6): For T1 with all UDP-sugars (MS detection), 0 min 5%; 2 min 5%; 17 min 85%; 18 min 100%; 23 min 100%; 24 min 5%; 25 min 5%; for T2 (UV detection) with UDP-GalNAc, 0 min 21.5%; 35 min 21.5%; 36 min 100%; 40 min 100%; 41

min 21.5%; 45 min 21.5%; with UDP-GalNAzMe, 0 min 21.5%; 35 min 21.5%; 36 min 100%; 40 min 100%; 41 min 21.5%; 45 min 21.5%; with UDP-GalNAz, 0 min 20%; 40 min 20%; 41 min 100%; 45 min 100%; 46 min 20%; 50 min 20%; with UDP-GalNPrAz, 0 min 20%; 40 min 20%; 41 min 100%; 45 min 100%; 46 min 20%; 50 min 20%; for T7 and T10 with all UDP-sugars (UV detection), 0 min 22.5%; 25 min 22.5%; 26 min 100%; 30 min 100%; 31 min 22.5%; 35 min 22.5%.

For reactions containing T1, extracted ion chromatograms (EIC) were generated of singly and doubly glycosylated species, integrated and related to the intensity of unglycosylated peptide. The m/z values $[M+2H]^{2+}$ used were 658.71 (EA2), 760.31 (EA2-GalNAc), 861.9 (EA2-2xGalNAc), 780.81 (EA2-GalNAz), 902.915 (EA2-2xGalNAz), 787.83 (EA2-GalNAzMe, EA2-GalNPrAz), 917.94 (EA2-2xGalNAzMe, EA2-2xGalNPrAz). Of note, total intensities of peptide and glycopeptide species inversely correlated with the abundance of doubly glycosylated species. As doubly glycosylated species were mainly present in glycosylations with UDP-GalNAc and UDP-GalNAz, we note that the abundance of unglycosylated peptides was likely overestimated for glycosylations using UDP-GalNAzMe and UDP-GalNPrAz.

Plasmids

The plasmids pIRES-puro containing $AGX1^{WT}$, $AGX1^{F381G}$, $AGX1^{F383G}$, $AGX1^{F381G/F383G}$, $AGX1^{F381A}$, $AGX1^{F383A}$, $AGX1^{F381A/F383A}$, pSBtet-$AGX1^{WT}$ and pSBtet-$AGX1^{F383A}$ were generated in our previous work (5, 8). The term “mut-AGX1” depicts $AGX1^{F383A}$ throughout this manuscript. GFP_CD47_SU (65473) were used to generate GFP::CD47 K562 cell lines. GFP_CD47_SU was a gift from Christine Mayr (Addgene plasmid # 65473 ; http://n2t.net/addgene:65473 ; RRID:Addgene_65473) (9). pCMV(CAT)T7-SB100 was a gift from Zsuzsanna Izsvak (Addgene plasmid #34879; http://n2t.net/addgene:34879 ; RRID:Addgene_34879) (10).

Cell transfection

Tet-system approved FBS (Takara) was used to propagate all cell lines transfected with pSBtet-based plasmids. K-562 cells were a gift from Jonathan Weissman (University of California, San Francisco). K-562 cells with stable expression of *Streptococcus pyogenes* Cas9 (K-562-spCas9) were prepared in-house. Cells were grown using RPMI with 10% (v/v) FBS, penicillin (100 U/mL) and streptomycin (100 µg/mL). Cells were transfected with pSBtet-based plasmids using Lipofectamine LTX (Thermo) according to the manufacturer’s instructions, with a 20:1 (m/m) mixture of pSBtet and pCMV(CAT)T7-SB100 plasmid DNA. After 24 h, cells were harvested and selected in growth medium containing 150 µg/mL hygromycin B (Thermo) for 7-10 days to obtain stable cells.
HepG2 cells (ATCC HB-8065) were propagated in low-glucose DMEM (Caisson Labs, Smithfield, USA) with 10% (v/v) FBS, penicillin (100 U/mL) and streptomycin (100 µg/mL). Cells were transfected with Lipofectamine 3000 (Thermo Fisher) according to the manufacturer’s instructions, using a 20:1 (m/m) mixture of pSBtet and pCMV(CAT)T7-SB100 plasmid DNA. After 24 h, medium was aspirated, and cells were treated with fresh growth medium containing 600 µg/mL hygromycin B (Invitrogen) for two weeks to obtain stable cells. Following selection, cells were propagated in 200 µg/mL hygromycin B in growth medium.
HEK293T (ATCC CRL-3216) were grown in DMEM (Thermo Fisher) with 10% (v/v) FBS (Thermo Fisher), penicillin (100 U/mL) and streptomycin (100 µg/mL, GE Healthcare, Chicago, USA). Cells were transfected with pIRES-puro3 plasmids containing AGX1 constructs using TransIT-293 (Mirus Bio LLC, Madison, USA) according to the manufacturer’s instructions and 37.5 µg DNA per 15 cm dish or 15 µg DNA per 10 cm dish. After 24 h, medium was aspirated, and cells were treated with fresh growth medium and compounds for analysis of nucleotide-sugar biosynthesis (see below).

Lysate labeling

Membrane lysate labeling was performed as previously reported (5). Briefly, HepG2 cells were fractionated using the Subcellular Fractionation Kit for Cultured Cells (Thermo Fisher) according to the manufacturer’s instructions. The membrane fraction was heat-inactivated to abrogate endogenous GalNAc-T activity. Glycosylation reactions were performed on 10 µg membrane

protein in 20 µL reaction volume containing 62.5 mM Tris-HCl (pH 7.4), 150 mM NaCl, 10 mM $MnCl_2$, 250 µM UDP-GalNAc analog and soluble GalNAc-T1 or T2 at a final concentration of 20 nM (T1) or 10 nM (T2) at 37 °C for 12 h. Reactions were heat-inactivated at 95 °C for 20 s and subsequently cooled to 4 °C. Then, azide-containing reaction mixtures were sequentially treated with equal volumes (1.25 µL each) of 2 mM biotin-PEG_4-alkyne (Thermo Fisher), 2 mM BTTAA (Click Chemistry Tools, Scottsdale, USA), 20 mM $CuSO_4$ and 100 mM sodium ascorbate (final concentrations 100 µM biotin probe, 100 µM BTTAA, 1 mM $CuSO_4$ and 5 mM sodium ascorbate). Reactions were performed at room temperature for 2 h and quenched with 50 mM EDTA. Protein mixtures were then subjected to SDS-PAGE and blotted on nitrocellulose membranes. The total protein amount was assessed using the REVERT protein staining kit (LI-COR Biosciences, Lincoln, USA), and biotin signal was detected using IRDye 800CW Streptavidin (LI-COR Biosciences) according to the manufacturer's instructions.

Modeling
The crystal structures of human GALE, GalNAc-T2 (PDB 4D0T), GalNAc-T7 (PDB 6IWR), GalNAc-T10 (PDB 2D7I) and AGX1 (PDB 1JV3) were visualized with Pymol 2.0.0 (Schrodinger LLC, New York) (11–15). Modelling of UDP-GalNAzMe into GALE and GalNAc-T2 was performed using simple minimization in COOT (16). Ligand restraints for UDP-GalNAzMe were generated using phenix.elbow (17).

***In vitro* epimerization assay**
In vitro epimeriszation was performed according to a literature precedent (4). Briefly, 5 million K-562 GALE-KO or control cells stably transfected with pSBtet-AGX1^{F383A} were harvested, washed once with PBS and frozen at -80 °C. Cells were thawed on ice and treated with the Cytosolic Extraction Buffer of the Subcellular Fractionation Kit for Cultured Cells (250 µL). Cells were lysed according to the manufacturer's instructions. The protein content of the cytosolic extract was assessed by BCA and equal protein amounts were used for *in vitro* epimerization. Assays contained 2 µL protein extract, 100 µM NAD and 1 mM UDP-sugar in 20 µL water. Reactions were carried out for 16 h at 37 °C, quenched by the addition of 1 µL 100 µM NaOH, and diluted to 50 µL with water. Samples were analyzed by HPAEC-PAD.

Analysis of nucleotide-sugar biosynthesis by High Performance Anion Exchange Chromatography
Cells expressing AGX1-FLAG (transient or stably transfected HEK293T in 20 mL growth medium in a 10 cm dish or 5 million K-562 or K-562 GALE-KO cells stably transfected with pSBtet-AGX1^{WT} or pSBtet-AGX1^{F383A} in 4 mL growth medium) were fed Ac_4GalNAz or caged GalNAzMe-1-phosphate analog **11** (100 µM final concentration from a 100 mM stock solution in DMSO) or DMSO vehicle. After 7 h, cells were harvested. K-562 cells were centrifuged at 500 *x g*, 5 min, 4 °C and resuspended in PBS (1 mL). HEK293T cells were washed once on the plate with cold PBS (8 mL), scraped in cold 1 mM EDTA in PBS (8 mL), transferred to a conical tube and harvested (300 g, 5 min, 4 °C). Cell pellets were resuspended in PBS (1 mL). 0.9 mL cell suspension was transferred to O-ring tubes (1.5 mL, Thermo Fisher) and centrifuged. Zirconia/silica beads (0.1 mm, BioSpec, Bertlesville, USA) were added at a similar volume to the cell pellet, followed by 1:1 acetonitrile/water (1 mL). Cells were lysed using a bead beater (FastPrep-24, MP Biomedicals, Santa Ana, USA) at 6 m/s for 30 s, and the lysate was cooled at 4 °C for 10 min. Samples were centrifuged (14000 *x g,* 10 min, 4 °C), and the supernatant was transferred to a new tube. The solvent was evaporated by speed vac. The residue was dissolved in LCMS-grade water (Thermo Fisher, 0.2-0.4 mL) containing 15 µM ADP-α-D-glucose (Sigma Aldrich). The solution was dialyzed (30 min, 14000 *x g*) using a 3 kDa Amicon Ultra Centrifugal Filter Unit (Merck). High performance anion exchange chromatography was used to analyze lysates.
The residual cell suspension in PBS (0.1 mL) was centrifuged, and the pellet was resuspended in M-PER lysis buffer (Thermo Fisher) with cOmplete protease inhibitor (0.2 mL). The solution was incubated at room temperature for 10 min and centrifuged (14000 *x g*, 10 min, 4 °C). The supernatant was transferred into a new tube, the protein concentration was measured by BCA, and samples were used for analysis of protein expression.

High performance anion exchange chromatography was carried out using an ICS-5000 with a quaternary pump and pulsed amperometric detection (Thermo Fisher) on a CarboPac PA1 4x250 mm column and a 4x50 mm guard column. Solvents were: A = 1 mM NaOH in degassed water; B = 1 mM NaOH, 1M NaOAc in degassed water. 0 min 5% B; 20 min 40% B; 60 min 40% B; 63 min 50% B; 83 min 50% B; 87 min 100% B; 95 min 100% B; 97 min 5% B; 105 min 5% B. Commercial or synthetic standards (200-500 µM) were used as controls.

Metabolic cell surface labeling, growth assessment, flow cytometry and in-gel fluorescence

K-562 cells stably transfected with pSBtet-AGX1^{WT} or pSBtet-AGX1^{F383A} were seeded into well plates at a density of 250,000 cells/mL in growth medium without hygromycin. Cells were treated with DMSO, caged GalNAc-1-phosphate analog **11**, Ac_4GalNAz, Ac_4ManNAz, or Ac_4GlcNAz at the indicated concentrations and using either GalNAc or GlcNAc as additives in the indicated concentrations. Cells were grown for another 20 h.
To assess cell growth, cells were re-fed after 24 h, and counted after a total of 48 h.
Cells were optionally treated with enzymes before fluorescence-based readout. StcE (50 nM final concentration) was added directly to the cell suspension, while for sialidase treatment, cells were harvested, washed once with serum-free RPMI media and treated with *Vibrio cholerae* sialidase (10 nM final concentration, generated in-house) in serum-free media (18). Enzyme treatment was performed for 2 h at 37 °C.

For in-gel fluorescence, cells were harvested in a V-shaped 96 well plate and washed twice with 2% FBS in PBS (Labeling Buffer, 0.2 mL). Cells were resuspended in Labeling Buffer (35 µL), treated with a solution of 200 µM $CuSO_4$, 1200 µM BTTAA (Click Chemistry Tools, Scottsdale, USA), 5 mM sodium ascorbate, 5 mM aminoguanidinium chloride and 200 µM CF680 picolyl azide in Labeling Buffer (35 µL), and incubated for 7 min at room temperature on an orbital shaker. The click reaction was quenched with 3 mM bathocuproinedisulfonic acid in PBS (35 µL). Cells were centrifuged, washed twice with Labeling Buffer and then with PBS, and treated with ice-cold Lysis Buffer (50 mM Tris-HCl pH 8, 150 mM NaCl, 1% (v/v) Triton X-100, 0.5% (v/v) sodium deoxycholate, 0.1% (w/v) SDS, 1 mM $MgCl_2$, and 100 mU/µL benzonase (Merck) containing cOmplete protease inhibitors, (0.1 mL). Cells were lysed for 20 min at 4 °C on an orbital shaker and centrifuged (1500 *x g*, 20 min, 4 °C). Supernatant was transferred to a new plate and BCA was used to measure protein concentration. For enzyme treatment, equal amounts of protein (typically 15 µg) were diluted to 40 µL with Lysis Buffer or PBS, treated with either SialEXO (4 µL of a 4 U/µL solution in 50 mM Tris-HCl (pH 6.5), Genovis, Lund, Sweden) or the glycoprotease StcE (50 nM in PBS) (19) and incubated for 2 h at 37 °C. The reaction was quenched by heating to 95 °C for 10 s with subsequent cooling at 4 °C. Loading buffer (a 1:1:1:0.5 (v/v/v/v) mixture of 1 M Tris-HCl pH 6.5, 80% (v/v) glycerol, 10% (w/v) SDS and 1 M DTT) was added, samples were heated at 95 °C for 30 s, run on a 10% Criterion™ gel (Bio-Rad, Hercules, USA) for SDS-PAGE, and imaged on an Odyssey CLx imager (LI-COR Biosciences, Lincoln, USA). Total protein was stained with Coomassie using Acquastain (Bulldog Bio, Portsmouth, USA). Protein expression was assessed by Western blot with a different set of samples, using antibodies against GALE (sc-390407, Santa Cruz Biotechnology, Dallas, USA), GAPDH (ab128915, Abcam) or FLAG tag (mouse anti-FLAG M2, Sigma Aldrich).

For flow cytometry, cells were harvested, washed twice with Labeling Buffer, resuspended in 100 µM DIBAC-sulfo-biotin (DBCO-sulfo-biotin, Jena Bioscience, Jena, Germany) in Labeling Buffer (100 µL) and incubated for 1 h at room temperature on an orbital shaker. Cells were washed twice, and treated with DTAF-streptavidin (1:000, Jackson ImmunoResearch, Cambridge, UK). Cells were incubated for 1 h at room temperature, washed twice and treated with SYTOX red (1:1000, Thermo) in Labeling Buffer. Flow cytometry was performed on an Accuri C6 flow cytometer (Becton Dickinson, Franklin Lakes, USA).

Superresolution microscopy

K-562 cells stably transfected with pSBtet-AGX1^{WT} or pSBtet-AGX1^{F383A} were seeded into well plates at a density of 180,000 cells in 680 µL growth medium without hygromycin. Cells were

treated with either DMSO, 100 µM compound **11** or 5 µM $Ac_4GalNAz$. Cells were incubated for 16 h. An 8-chamber coverslip (Lab-Tek II 155409, Thermo) was coated with 250 µL human Fibronectin (20 µg/mL, Sigma Aldrich) for 1 h at 37 °C, and washed with PBS. Cells were transferred to one well each and further incubated for 5 h at 37 °C. Cells were moved to 4 °C, medium was aspirated, and cells were washed with ice-cold PBS (4x 300 µL). Cells were then treated first with 100 µL PBS and 100 µL of a freshly prepared solution containing 1200 µM BTTAA, 200 µM CuSO4, 5 mM sodium ascorbate, 5 mM aminoguanidinium chloride and 400 µM biotin-PEG3-alkyne (Click Chemistry Tools). The reaction was carried out for 6 min at 4 °C, the supernatant was removed and cells were washed with PBS (4x 300 µL). Cells were incubated with 20 µg/mL AF647-streptavidin (Jackson ImmunoResearch) for 30 min at 4 °C, then washed again. Cells were fixed with 4% (v/v) paraformaldehyde (Thermo) and 0.2% (v/v) glutaraldehyde (Sigma Aldrich) in PBS for 30 min at 4 °C, then washed with PBS (4x300 µL).
The instrument setup is based on an inverted microscope (IX71, Olympus, Tokyo, Japan). The laser used for illumination (120 mW 647 nm, CW, Coherent, Santa Clara, CA) was spectrally filtered (ff01-631/36-25 excitation filter, Semrock, Rochester, NY) and circularly polarized (LPVISB050-MP2 polarizers, Thorlabs, Newton, NJ, WPQ05M-633 quarter-wave plate, Thorlabs). The beam was expanded and collimated using Keplerian telescopes. Shutters were used to toggle the lasers (VS14S2T1 with VMM-D3 driver, Vincent Associates Uniblitz, Rochester, NY). The laser was introduced into the back port of the microscope via a Köhler lens. The sample was mounted onto an XYZ stage (PInano XYZ Piezo Stage and High Precision XY Microscope Stage, Physik Instrumente, Karlsruhe, Germany). Emitted light was detected using a high NA detection objective (UPLSAPO100XO, x100, NA 1.4, Olympus) and spectrally filtered (Di01-R405/488/561/635 dichroic, Semrock; ZET647NF notch filter, Chroma, Bellows Falls, VT; ET700/75m bandpass filter, Chroma, 3RD650LP longpass filter, Omega Optical, Austin, TX), and focused by the microscope tube lens. The emitted light entered a 4f imaging system (f= 90 mm) and was focused onto an EMCCD camera (iXon3 897, Andor, Belfast, UK) by the second lens of the 4f imaging system.

PBS was replaced by a reducing, oxygen scavenging buffer (20), consisting of 20 mM cysteamine, 2 µL/mL catalase, 560 µg/mL glucose oxidase (all Sigma-Aldrich), 10% (w/v) glucose (BD Difco, Franklin Lakes, USA), and 100 mM Tris-HCl (Life Technologies). Imaging was performed at 647 nm excitation with a laser intensity of 5 kW/cm^2. The exposure time was 50 ms and the calibrated EM gain was 186. SR reconstructions were reconstructed from approx. 40000 frames using the ImageJ plugin Thunderstorm (21). Images were filtered with a B-spline filter of order 3 and scale 2.0. Single-molecule signals were detected with 8-neighborhood connectivity and a threshold of three times the standard deviation of the first wavelet level. Detected local maxima were fitted with a 2D-Gaussian using least squares. Drift correction was done by cross-correlation, followed by filtering (σ of the fitted Gaussian <300 nm; uncertainty of localization <30 nm). Images were reconstructed as 2D histograms with a bin size of 32 nm, corresponding to a five-time magnification compared to the pixel size of 160 nm.

PEG Mass Tagging
Mass tagging was performed according to Woo et al. (22). Briefly, K-562 or K-562 GALE-KO cells stably transfected with pSBtet-$AGX1^{WT}$ or pSBtet-$AGX1^{F383A}$ were fed with azide-containing sugars as described above, and lysed using approx. 100 µL /500,000 cells of Lysis Buffer supplemented with 50 µM PUGNAc (Sigma Aldrich). Lysate corresponding to 30 µg protein was treated with 20% (v/v) to a final concentration of 1% and incubated for 10 min at 65 °C. The solution was treated with iodoacetamide (Sigma Aldrich) to a final concentration of 15 mM, and incubated in the dark for 30 min at room temperature. DIBAC-PEG 10 kDa (DBCO-PEG 10, Jena Bioscience) was added to a final concentration of 200 µM, and the solution was incubated overnight at room temperature. A 1:1 (v/v) mixture of 1 M Tris-HCl (pH 6.5) and 80% (v/v) glycerol was added (10% of the final volume), and mass tagging was assessed by Western Blot.

Genome-wide CRISPR knockout screen
A CRISPR sgRNA library targeting all 20,500 human protein-coding genes was synthesized, cloned and packaged into lentivirus as described previously (23). 250 million K-562-Cas9 cells

stably transfected with pSBtet-AGX1^{F383A} were infected with lentivirus at a multiplicity of infection of 0.4 for 24 h in media containing 8 µg/mL polybrene. Cells were subsequently selected for 128 h with 1 µg/mL puromycin, then changed into fresh media without puromycin and allowed to recover for 24 h. Cells were pooled, harvested in four batches of 50 million cells each, and resuspended in 200 mL medium each. Cells were treated with caged GalNAc-1-phosphate analog **5** (100 µM as 200 µL of a 100 mM stock solution in DMSO diluted to 2 mL with medium), DMSO (200 µL diluted to 2 mL with medium), or Ac$_4$GalNAz (10 µM as 200 µL of a 10 mM stock solution in DMSO diluted to 2 mL with medium). Two t = 0 samples of 100 million cells each were washed with PBS once and frozen at -80 C.

After 20 h, cells were harvested (500 *x g*, 5 min, 4 °C) and washed twice with Labeling Buffer. The DMSO-treated samples were frozen at -80 °C except for a 400 µL aliquot that was harvested and resuspended in 400 µL 50 µM MB488-DIBAC (MB488-DBCO, Click Chemistry Tools) in Labeling Buffer.

GalNAc analog treated samples were resuspended in 50 µM MB488-DIBAC in Labeling Buffer (50 mL, MB488-DBCO, Click Chemistry Tools). Samples were incubated for 30 min at room temperature. Cells were harvested, washed twice with Labeling Buffer resuspended as a 15 million cells/mL suspension containing 5 nM SYTOX Red. Intact, viable cells were defined by sorting on FSC/SSC and SYTOX Red channels. A cell population representing both the top and bottom 15% of the fluorescence distribution for GalNAz/GalNAzMe was then isolated. Sorting was conducted until at least 50 million events (cells) had been processed for each sample. Sorted cells were then pelleted and frozen at -80 °C in preparation for subsequent processing. Aliquots of 50 million unsorted cells from a DMSO-treated sample were also pelleted and frozen down in parallel for normalization.

CRISPR Screen DNA Extraction and Data Analysis: Frozen cell pellets were thawed and genomic DNA extraction was performed using either the QIAamp DNA Blood Maxi Kit (Qiagen, Hilden, Germany) for unsorted samples or the GeneElute Mammalian Genomic DNA Miniprep kit (Sigma) for sorted samples according to manufacturer's specifications. The sgRNA-encoding regions were amplified via nested PCR and sequenced on a NextSeq500 (Illumina, San Diego, USA). Reads were aligned to the sgRNA library and the log2 fold change was calculated for each sgRNA. Median phenotypes for each gene were calculated as previously described (24). *P*-values were calculated using a Mann-Whitney U-test and adjusted FDRs were computed using the Benjamini–Hochberg procedure.

Click & enrichment of HepG2 secretome

HepG2 cells stably transfected with pSBtet-AGX1^{F383A} were seeded into one 10 cm dish per treatment (8 mL) without hygromycin B. After 24 h (40% confluency), cells were fed with either GalNAzMe (100 µM), Ac$_4$GalNAz (3µM), or DMSO vehicle. After another 24 h, the medium was aspirated, cells were washed with pre-warmed serum-free low-Glucose DMEM and fed with GalNAzMe (100 µM), Ac$_4$GalNAz (3µM) or DMSO in serum-free medium and incubated for 20 h. Conditioned supernatant was collected and centrifuged at 500 *x g* for5 min. The supernatant was concentrated to 2 mL using an Amicon Ultra-15 Centrifugal Filter Unit (3 kDa MWCO, Merck). Samples were treated with PNGase F (Promega, 5 µL of a 1:10 dilution in PBS) and incubated for 4 h at 37 °C. Then, azide-containing reaction mixtures were sequentially treated with 1200 µM BTTAA (stock solution 50 mM in 9:1 DMSO : water), 600 µM CuSO4 (stock solution 20 mM in water), 5 mM sodium ascorbate, 5 mM aminoguanidine chloride, and 100 µM DADPS Biotin Alkyne (Click Chemistry Tools, stock solution 10 mM in DMSO). The click reaction was carried out for 3 h at room temperature with inversion. Then, samples were transferred into 15 mL Falcon tubes and treated with 10 mL (5-fold excess) ice-cold methanol. Samples were left at -80 °C overnight, when a white precipitate had formed. Samples were centrifuged at 3700 *x g*, 4 °C, 20 min. Supernatant was discarded, and pellets were washed with 5 mL methanol twice, with centrifugation each time. Supernatant was completely removed by air-drying, and samples were treated with 250 µL 0.1% RapiGest in PBS. Samples were sonicated (water bath) for 25 min, then centrifuged at 3700 *x g* for 5 min. Supernatant was saved, and pellets were treated with 250 µL 6 M urea in PBS. Samples were sonicated and centrifuged again, and the supernatant was saved. The pellets were treated with 250 µL of PBS, sonicated and centrifuged again. RapiGest, urea and PBS supernatants were combined, and samples were diluted with PBS to 2 mL.

Dimethylated Sera-Mag SpeedBeads Neutravidin Magnetic Beads (150 µL slurry = 75 µL settled resin) were washed with PBS twice in LoBind tubes (Eppendorf) and added to the lysate (25, 26). Samples were incubated for 16 h at 4 °C under rotation. The beads were harvested and the supernatant was discarded. The beads were washed sequentially with 1% RapiGest in PBS (3x), 6 M urea in PBS (3x), and PBS (2x), and resuspended in PBS (200 µL). Beads were treated with 100 mM DTT in PBS (10 µL), and shaken for 30 min, at room temperature, 950 rpm. Then, 500 mM iodoacetamide in PBS (4 µL) was added and samples were shaken for another 30 min in the dark. Beads were harvested and washed with PBS and 50 mM ammonium bicarbonate in LC/MS-grade water (3x, "ABC buffer"). Beads were resuspended in ABC buffer (200 µL), treated with RapiGest to a final concentration of 0.05% (v/v), and LysC was added (500 ng in 3 µL of ABC buffer). Samples were shaken at 37 °C for 2-3 h, and another 500 ng LysC was added. The reactions were shaken overnight at 37 °C. The beads were harvested, washed with ABC (200 µL) and with LC-MS grade water (3x200 µL). Supernatants and washes were combined and centrifuged (18000 *x g*, 5 min, RT) and concentrated by SpeedVac which formed the peptide fraction to be analysed by mass spectrometry. Then, beads were treated with 150 µL of 0.1% aq. formic acid (FA, Optima grade, Thermo Fisher) and shaken for 30 min, at room temperature, 950 rpm. This step was repeated, beads were washed with 100 µL of water, all washes were combined and centrifuged (18000 *x g*, 5 min, room temperature), and finally concentrated by SpeedVac. Remaining beads were treated with 2% FA and subjected to the same washes as above. All samples were then resuspended with 25 ng trypsin in 100 µL of 50 mM ABC buffer and incubated for 6 h at 37 °C, 450 rpm. Samples were then dried by SpeedVac and desalted by Strata-X columns using 0.1% aq. FA as washing solution and 80% MeCN/water with 0.1% FA (v/v) as elution buffer. The eluted samples dried by SpeedVac.

Peptides dried by vacuum centrifugation into 0.5 mL LoBind tubes (Eppendorf) were resuspended into 16 µL of 0.1 % (v/v) FA with sonication using an ultrasonic water bath followed by vortexing. The solubilised peptides were centrifuged for 5 min at 18,000 *x g* and the solution transferred into Total Recovery vials (Waters, Milford, USA) for injection. Samples were analysed by online nanoflow LC-MS/MS using an Orbitrap Fusion Lumos mass spectrometer (Thermo Scientific) coupled to an Ultimate 3000 RSLCnano (Thermo Scientific). Sample (15 µL) was loaded via autosampler into a 20 µL sample loop and pre-concentrated onto an Acclaim PepMap 100 75 µm x 2 cm nanoviper trap column with loading buffer, 2% v/v acetonitrile, 0.05% v/v trifluoroacetic acid, 97.95% water (Optima grade, Fisher Scientific) at a flow rate of 7 µL/min for 6 min in the column oven held at 40 ºC. Peptides were gradient eluted onto a C_{18} 75 µm x 50 cm, 2 µm particle size, 100Å pore size, reversed phase EASY-Spray analytical column (Thermo Scientific) at a flow rate of 275 nL/min and with the column temperature held at 40 ºC, and a spray voltage of 2100 V using the EASY-Spray Source (Thermo Scientific). Gradient elution buffers were A 0.1% v/v FA, 5% v/v DMSO, 94.9% v/v water and B 0.1% v/v FA, 5% v/v DMSO, 20% v/v water, 74.9% v/v acetonitrile (all Optima grade, Fisher Scientific aside from DMSO, Honeywell Research Chemicals). The gradient elution profile was 2% B to 40% B over 98 minutes. The instrument method used an MS1 Orbitrap scan resolution of 120,000 at FWHM m/z 200, quadrupole isolation, mass range 300-1500 m/z, RF Lens 30%, AGC target 4e5, maximum injection time 50 ms and spectra were acquired in profile. Monoisotopic Peak Determination was set to the peptide mode, and only precursors with charge states 2-6 were permitted for selection for fragmentation. Dynamic Exclusion was enabled to exclude after n=3 times within 10 s for 10 s with high and low ppm mass tolerances of 10 ppm. HCD was performed on all selected precursor masses using a cycle time-based data dependant mode of acquisition set to 3 s. MS2 scans were acquired in the Orbitrap at a resolution of 30000 FWHM m/z 200, following HCD fragmentation with fixed collision energy of 28% after quadrupole isolation with an isolation window width of 2 m/z. The parameters used for the HCD MS2 scan were first mass 100 m/z, AGC target 5e4, maximum injection time 54 ms and the scan data was acquired in centroid mode. ETD fragmentation was only performed if precursors were within the precursor selection range m/z 300-1000 and if 2 of the following list of mass trigger ions were present in the HCD MS2 spectra +/- 0.1 m/z and above the relative intensity threshold of 10% (126.055, 138.0549, 144.0655, 168.0654, 186.076, 204.0855, 274.0921, 292.1027, 343.1617, 329.1461 m/z). ETD MS2 scans were recorded in the ion trap with rapid scan rate following quadrupole isolation with

an isolation window width of 3 m/z. ETD activation used calibrated charge-dependent ETD parameters, the automatic scan range mode was used with the first mass set to 100 m/z and parameters were set for the AGC target 1e4, maximum injection time 100 ms and scan data acquired in centroid mode.

Data evaluation was performed with Byonic™ (Protein Metrics, Cupertino, USA). Data files were first searched against the Uniprot human proteome (downloaded June 26, 2016). Search parameters included semi-specific cleavage specificity at the C-terminal site of R and K, with two missed cleavages allowed. Mass tolerance was set at 10 ppm for MS1s, 0.1 Da for HCD MS2s, and 0.35 Da for ETD MS2s. Methionine oxidation (common 2), asparagine deamidation (common 2), and N-term acetylation (rare 1) were set as variable modifications with a total common max of 3, rare max of 1. Cysteine carbamidomethylation was set as a fixed modification. Peptide hits were filtered using a 1% FDR. Additionally, a cut-off value of Log Prob = 5 was set for any further analysis. Proteins that were found in these searches were entered into a "focused database" for glycopeptide searches. Then, the raw files were searched against these "focused databases" containing only those proteins found in the corresponding peptide samples. Search parameters for the glycopeptide analysis included semi-specific cleavage specificity at the C-terminal site of R and K, with two missed cleavages allowed. Mass tolerance was set at 10 ppm for MS1s, 0.1 Da for HCD MS2s, and 0.35 Da for ETD MS2s. Methionine oxidation (common 2), asparagine deamidation (common 2), and N-term acetylation (rare 1) were set as variable modifications with a total common max of 2, rare max of 1. O-glycans were also set as variable modifications (common 2), using a custom database, whereby HexNAc, HexNAc-NeuAc, HexNAc-Hex, HexNAc-Hex-NeuAc, and HexNAc-Hex-NeuAc2 were searched with an additional 139.0746 (GalNAzMe) or 125.0589 (GalNAz) to account for the chemical modifications. HCD was used to confirm that the peptides were glycosylated, as the modified sugars have signature ions present at 343.1617/325.1506 m/z (GalNAzMe) or 329.1461/311.1435 m/z (GalNAz). Following confirmation, ETD spectra were used for site-localisation of glycosylation sites. All spectra with these modifications were manually annotated.

Organoid culture and generation of stably overexpressing organoid lines
Organoids were established from freshly isolated wild type small intestine from adult mice, as previously described (27). Upon isolation intestinal crypts were cultured in Cultrex® BME, Type 2 RGF PathClear (Amsbio, 3533-010-02) and IntestiCult™ Organoid Growth Medium (Stem Cell technologies, #06005) was used to drive differentiation of all epithelial cell types. Generation of stably overexpressing organoid lines was performed as previously described (28). Briefly, organoids were dissociated in single cells with Accumax (Merck) and counted. 500,000 cells were electroporated using a NEPA21 electroporator with pSBtet-GalNAc-T2^{WT}-AGX1^{F383A} or pSBtet-GalNAc-T2^{DM}-AGX1^{F383A} and seeded at high confluency. Organoids were treated with Rho kinase inhibitor Y-27632 (Sigma Aldrich, Y0503) and Gsk3 inhibitor CHIR99021 (Tocris, 4423) for 48 h prior to and after electroporation and with DMSO for 24 h prior to and after electroporation to increase efficiency. After 4 days, organoids were treated with fresh medium containing 200 µg/ml Hygromycin B.

Organoid labelling and immunofluorescence
Organoids were seeded on an 8-well glass chamber slide (Nunc, 154534), allowed to establish for 72 h and, subsequently, fed twice with 1.5 µM Ac_4GalNAz or 50 µM compound **11** for 2 days. After feeding, organoids were washed twice with PBS and fixed with 10% Formalin for 20 min at room temperature. The reaction was quenched with addition of 50 mM NH_4Cl for 5 min. Organoids were then treated with 150 µL PBS and 150 µL of a freshly prepared solution containing 1200 µM BTTAA, 200 µM CuSO4, 10 mM sodium ascorbate, 10 mM aminoguanidine chloride and 200 µM biotin-PEG3-alkyne (Click Chemistry Tools) for 10 min, then washed with PBS (3 x 300µL), and incubated with 20 µg/mL AF647-streptavidin (Jackson ImmunoResearch) for 30 min at 4 °C. After washing with PBS (3 x 300µL), organoids were permeabilized with 0.8 % Triton X-100 for 20 min at room temperature, blocked with 1 % BSA for 30 min at room temperature and incubated with Phalloidin-FITC (Merck, P5282) and 4',6'-diamidino-2-phenylindole (DAPI) for 1 h at room temperature. Slides were washed (3 x 300µL) and mounted

with ProLong Gold Antifade mountant (Thermo Fisher, P36934). Samples were imaged using a Leica TCS SPE confocal microscope.

Synthetic chemistry

Solvents and reagents were of commercial grade. Anhydrous solvents were obtained from a Dry Solvent System. Water-sensitive reactions were carried out in heat-dried glassware and under a nitrogen atmosphere. Thin layer chromatography was performed on Kieselgel 60 F254 glass plates pre-coated with silica gel (0.25 mm thickness). Spots were developed with ceric ammonium molybdate stain (5% (w/v) ammonium molybdate, 1% (w/v) cerium (II) sulfate and 10% (v/v) sulfuric acid in water) or sugar stain (0.1% (v/v) 3-methoxyphenol, 2.5% (v/v) sulfuric acid in EtOH) dipping solutions. Flash chromatography was carried out on Fluka Kieselgel 60 (230-400 mesh). Solvents were removed under reduced pressure using a rotary evaporator and high vacuum (1 mbar). Medium pressure chromatography was performed on an Isolera Prime system (Biotage, Uppsala, Sweden).

^{1}H, ^{13}C and 2D NMR spectra were measured with an AS400 spectrometer, an AS600 spectrometer (Varian, Palo Alto, USA) or a Bruker Avance-400 MHz spectrometer at 298 K. Chemical shifts (σ) are reported in parts per million (ppm) relative to the respective residual solvent peaks ($CDCl_3$: σ 7.26 in ^{1}H and 77.16 in ^{13}C NMR; acetone-D_6: σ 2.05 in ^{1}H and 29.84 in ^{13}C NMR). Two-dimensional NMR experiments (HH-COSY, CH-HSQC) were performed to assign peaks in ^{1}H spectra. The following abbreviations are used to indicate peak multiplicities: s singlet; d doublet; dd doublet of doublets; dt doublet of triplets; m multiplet. Coupling constants (*J*) are reported in Hertz (Hz). High resolution mass spectrometry by electrospray ionization (ESI-HRMS) was performed at Stanford University Mass Spectrometry, with a micrOTOF-Q II hybrid quadrupole time-of-flight mass spectrometer (Bruker, Billerica, USA) equipped with a 1260 UPLC (Agilent). Low resolution mass spectrometry by electrospray ionization (ESI-LRMS) was performed on an UPLC-MS (Waters) equipped with ACQUITY UPLC® BEH C18 column. HPLC purification was performed on Perkin Elmer 200 Series equipped with ZORBAX 300SB-C8 column (21.2x250 mm, 7 μm), UV detector and a flow rate of 8 mL/min. Elution was monitored at 214 nm.

Compounds **SI-1**, **3**, **4**, **5**, **6**, **7**, **8**, **9** and **10** were made previously (6). Compounds **1**, **2**, UDP-GlcNAc and UDP-GlcNAz are commercially available (Thermo).

Compound Characterization

Bis(*S*-acetyl-2-thioethyl) 3,4,6-tri-*O*-acetyl-2-[2-(*S*)-azidopropionamido]-2-deoxy-2-α-D-galactopyranosyl phosphate (11)

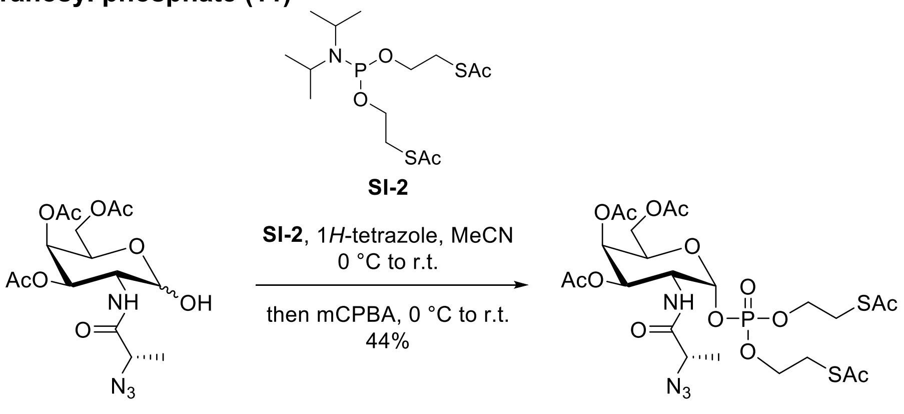

SI-1 **11**

To a stirred solution of lactol **SI-1**(6) (100 mg, 249 μmol) and phosphoramidite **SI-2**(29) (128 mg, 347 μmol) in MeCN (1.6 mL) was added at 0 °C 1*H*-tetrazole (26 mg, 371 μmol, 867 μL of a 3% (w/v) solution in MeCN). The reaction was warmed to room temperature and stirred for 1 h. The mixture was cooled to 0 °C and treated with mCPBA (64 mg, 372 μmol). The mixture was warmed to room temperature, stirred for 30 min, diluted with EtOAc (10 mL) and quenched with 10% aq. Na_2SO_3 (10 mL). The solution was washed with 10% aq. Na_2SO_3 (10 mL), sat. aq. $NaHCO_3$ (2x30

mL) and brine (50 mL). After every washing step, the aqueous layer was back-extracted with EtOAc (10 mL). The combined organic layers were dried over $MgSO_4$, filtered and concentrated. The residue was purified by flash chromatography (hexanes/EtOAc 1:1 to 0:1) to give phosphotriester **11** (75 mg, 109 µmol, 44%) as a clear oil. ^{1}H NMR (400 MHz, acetone-D_6) δ 7.59 (d, *J* = 8.3 Hz, 1H), 5.88 – 5.77 (m, 1H), 5.58 – 5.47 (m, 1H), 5.25 (dd, *J* = 11.7, 3.2 Hz, 1H), 4.63 – 4.45 (m, 2H), 4.32 – 3.98 (m, 7H), 3.30 – 3.14 (m, 4H), 2.36 (d, *J* = 1.2 Hz, 6H), 2.16 (s, 3H), 2.01 (s, 3H), 1.94 (s, 3H), 1.45 (d, *J* = 6.9 Hz, 3H); ^{13}C NMR (100 MHz, acetone-D_6) δ 195.2, 195.0, 172.0, 170.7, 170.6, 170.5, 97.2, 69.6, 67.9, 67.8, 67.1, 67.0, 67.0, 62.4, 58.7, 48.7, 20.7, 20.6, 20.6, 17.5; HRMS (ESI) calcd. for $C_{23}H_{35}N_4O_{14}PS_2$ (M+Na+) 709.1227 found 709.1219 *m/z*.

2-[(*S*)-Azidopropionamido]-2-deoxy-1,3,4,6-tetra-*O*-acetyl-D-glucopyranose (SI-3)

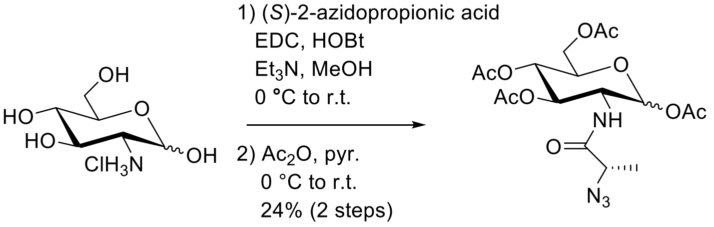

SI-3

To a stirred solution of acid (*S*)-2-azidopropionic acid(30) (1.7 g, 14.8 mmol) in MeOH (60 mL) were added D-glucosamine hydrochloride (1.32 g, 6.1 mmol) and triethylamine (2.28 mL, 16.28 mmol). The solution was cooled to 0 °C, and EDC (2.53 g, 16.28 mmol) and HOBt (1.13 g, 7.4 mmol) were added. The reaction was warmed to room temperature and was stirred for 72 h. The mixture was concentrated and passed twice through a plug of silica gel (CH_2Cl_2/MeOH 9:1) to give a residue that was dissolved in MeOH (2.5 mL), treated with $CHCl_3$ (100 mL) and precipitated at -20 °C to give the intermediary amide as a yellow precipitate that still contained traces of EDC and HOBt.

To a stirred solution of the intermediary amide in anhydrous pyridine (11 mL) was added at 0 °C acetic anhydride (6.3 mL, 66.3 mmol). The reaction was warmed to room temperature and stirred for 6 h. The mixture was diluted with water (100 mL) and EtOAc (100 mL), and the layers partitioned. The aqueous phase was extracted with EtOAc (3x50 mL), the combined organic extracts were washed with 0.2 M aq. HCl (3x50 mL), water (50 mL) and brine (50 mL), dried over $MgSO_4$, filtered and concentrated. The residue was co-evaporated with toluene (20 mL) and purified by flash chromatography (hexanes/EtOAc 1:0 to 1:3 to 1:2 to 1:1) to give tetraacetate **SI-3** (642 mg, 1.44 mmol, 24% over two steps, 7:3 α:β) as a clear oil. ^{1}H NMR (400 MHz, $CDCl_3$) δ 6.78 – 6.62 (m, 0.3H), 6.45 (d, *J* = 8.7 Hz, 0.7H), 6.17 (t, *J* = 3.7 Hz, 0.7), 5.76 (dd, *J* = 8.7, 1.7 Hz, 0.3H), 5.37 – 4.96 (m, 2H), 4.45 – 3.76 (m, 5H), 2.27 – 1.79 (m, 12H), 1.50 – 1.30 (m, 3H); ^{13}C NMR (100 MHz, $CDCl_3$) δ 171.5, 170.8, 170.7, 170.7, 170.5, 170.2, 169.4, 169.3, 169.2, 168.7, 92.3, 90.2, 72.9, 72.0, 70.3, 69.9, 68.0, 67.5, 61.7, 61.6, 59.3, 59.1, 53.0, 51.4, 29.7, 20.9, 20.8, 20.8, 20.7, 20.7, 20.7, 20.7, 20.6, 17.2, 17.2. HRMS (ESI) calcd. for $C_{17}H_{24}N_4O_{10}$ (M+Na$^+$) 467.1390 found 467.1387 *m/z*.

2-[(*S*)-Azidopropionamido]-2-deoxy-3,4,6-tri-*O*-acetyl-D-glucopyranose (SI-4)

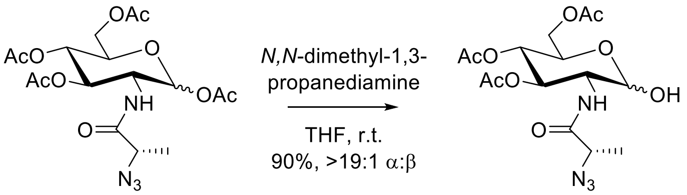

SI-3 **SI-4**

To a stirred solution of tetraacetate **SI-3** (320 mg, 0.72 mmol) in THF (3.5 mL) was added 3-(dimethylamino)-1-propylamine (0.26 mL, 2.15 mmol). The reaction was stirred at room temperature for 2 h, diluted with CH_2Cl_2 (50 mL), washed with 1 N aq. HCl (2x20 mL) and brine (20 mL). The organic phase was dried over $MgSO_4$, filtered and concentrated. The residue was purified by flash chromatography (hexanes/EtOAc 1:0 to 2:1) to give lactol **SI-4** (263 mg, 0.65 mmol, 90%, >19:1 α:β) as a clear oil. Rf (hexanes/EtOAc 3:2) = 0.35. ^{1}H NMR (400 MHz, $CDCl_3$) δ 6.68 (d, *J* =

9.3 Hz, 1H), 5.31 (t, J = 9.4 Hz, 1H), 5.23 (s, 1H), 5.11 (t, J = 9.4 Hz, 1H), 4.29 – 4.17 (m, 3H), 4.14 – 4.06 (m, 1H), 4.01 – 3.92 (m, 1H), 2.07 (s, 3H), 2.02 (s, 3H), 1.99 (s, 3H), 1.46 (d, J = 7.1 Hz, 3H); ^{13}C NMR (100 MHz, $CDCl_3$) δ 171.3, 171.1, 170.6, 169.6, 91.4, 70.9, 68.3, 67.6, 62.2, 58.9, 52.5, 20.9, 20.8, 20.7, 17.2; HRMS (ESI) calcd. for $C_{15}H_{22}N_4O_9P$ (M+Na$^+$) 425.1285 found 425.1283 *m/z.*

Bis-*O*-allyl 2-[(*S*)-Azidopropionamido]-2-deoxy-3,4,6-tetra-*O*-acetyl-α-D-glucopyranosyl phosphate (SI-5)

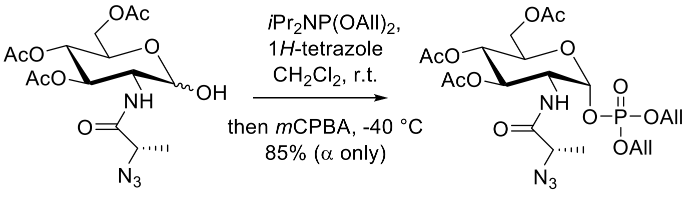

SI-4 **SI-5**

Lactol **SI-4** (210 mg, 0.52 mmol) and 1*H*-tetrazole (174 mg, 2.48 mmol) were co-evaporated with anhydrous toluene (5 mL), suspended in anhydrous toluene (5 mL) and sonicated for 1 h at room temperature in a bath sonicator. The solvent was evaporated, and the residue dissolved in anhydrous CH_2Cl_2 (7.8 mL). The stirred solution was cooled to 0 °C, and diallyl *N*,*N*-diisopropylphosphoramidite (220 µL, 0.83 mmol) was added. After 20 min, the solution was cooled to -40 °C, and mCPBA (222 mg, 0.99 mmol) was added. Another 126 mg (0.56 mmol) mCPBA was added after 10 min to drive the reaction to completion. After another 10 min, the reaction was quenched with 20% aq. Na_2SO_3 (10 mL), and warmed to room temperature. The mixture was diluted with CH_2Cl_2 (20 mL), and the layers were separated. The organic phase was washed with sat. aq. $NaHCO_3$ (10 mL), and the combined aqueous phase was re-extracted with CH_2Cl_2 (20 mL). The combined organic phase was washed with brine (20 mL), the organic phase wash dried over $MgSO_4$, and concentrated. The residue was purified by flash chromatography (hexanes/EtOAc 1:0 to 1:2 to 3:2) to give phosphate **SI-5** (248 mg, 0.44 mmol, 85%, α anomer only) as a clear oil. Rf (hexanes/EtOAc 3:2) = 0.3. ^{1}H NMR (400 MHz, $CDCl_3$) δ 6.74 (d, J = 8.9 Hz, 1H), 6.00 – 5.81 (m, 2H), 5.67 (dd, J = 6.2, 3.3 Hz, 1H), 5.41 – 5.19 (m, 5H), 5.14 (t, J = 9.7 Hz, 1H), 4.61 – 4.49 (m, 4H), 4.31 (m, 1H), 4.24 – 4.12 (m, 2H), 4.05 (dd, J = 12.2, 2.0 Hz, 1H), 3.96 (q, J = 7.0 Hz, 1H), 2.03 (s, 3H), 2.00 (s, 3H), 1.98 (s, 3H), 1.44 (d, J = 7.0 Hz, 3H); ^{13}C NMR (100 MHz, $CDCl_3$) δ 171.1, 170.6, 170.5, 169.2, 132.0, 132.0, 132.0, 131.9, 119.1, 119.0, 95.7, 95.6, 69.9, 69.7, 68.9, 68.85, 68.8, 68.7, 67.4, 61.5, 58.9, 52.2, 52.1, 20.7, 20.6, 20.6, 17.2; HRMS (ESI) calcd. for $C_{21}H_{31}N_4O_{12}P$ (M+Na$^+$) 585.1573 found 585.1565 *m/z*.

Uridine 5'-diphospho-2-((*S*)-azidopopionamido)-2-deoxy-α-D-glucopyranoside tributylammonium salt (SI-7)

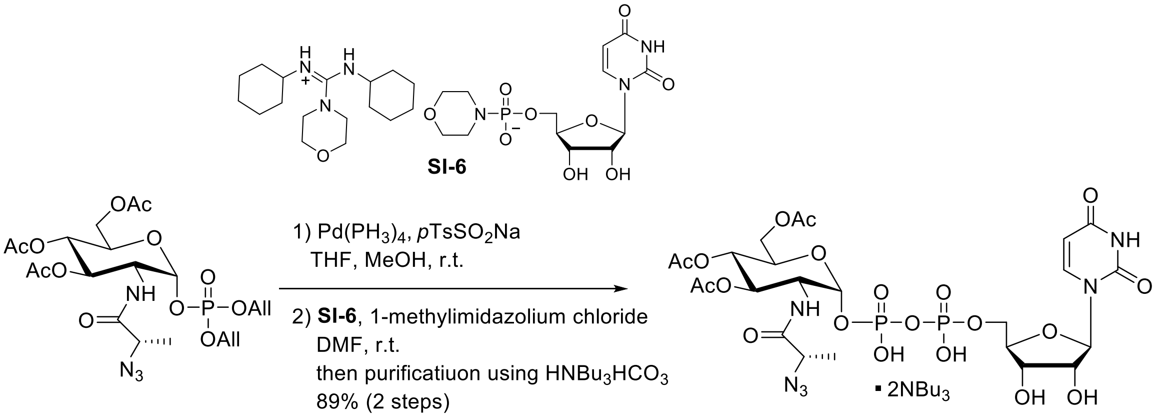

SI-5 **SI-7**

To a stirred solution of diallyl phosphotriester **SI-5** (50 mg, 89 µmol) in THF/MeOH (1:1, 1.78 mL) were added tetrakis(triphenylphosphine)palladium (5 mg, 4.5 µmol) and sodium *para*-

toluoenesulfinate (32 mg, 178 µmol). The reaction was stirred at room temperature for 3 h, and tetrakis(triphenylphosphine)palladium (5 mg, 4.5 µmol) and sodium *para*-toluoenesulfinate (32 mg, 178 µmol) were added to drive the reaction to completion. The mixture was stirred at room temperature for 16 h, and the solvents were evaporated. The residue was co-evaporated with anhydrous toluene (2x5 mL) and dissolved in DMF (1.78 mL). Uridine monophosphomorpholidate **SI-6** (98 mg, 145 µmol) and 1-methylimidazolium chloride (57 mg, 0.49 µmol) were added and the reaction was stirred at room temperature for 16 h. The mixture was concentrated and purified by medium-pressure flash chromatography (60 g SNAP C18 column; A: 10 mM tributylammonium bicarbonate,(6) B: MeOH; 6 column volumes 100% A; 9 column volumes linear gradient to 100% B, then 6 column volumes 100% B), and fractions were concentrated and lyophilized repeatedly to give pyrophosphate **SI-7** as the tributylammonium salt (92 mg, 79 µmol, 89% over two steps) as a white foam. Rf (CH_2Cl_2/MeOH 2:1 + 1% AcOH) = 0.3. ^{1}H NMR (600 MHz, CD_3OD) δ 8.08 (d, *J* = 8.2 Hz, 1H), 5.93 (d, *J* = 4.4 Hz, 1H), 5.81 (d, *J* = 8.1 Hz, 1H), 5.65 (dd, *J* = 7.3, 3.3 Hz, 1H), 5.28 (t, *J* = 10.0 Hz, 1H), 5.11 (t, *J* = 9.8 Hz, 1H), 4.47 – 4.04 (m, 10H), 2.05 (s, 3H), 1.98 (s, 3H), 1.92 (s, 3H), 1.37 – 1.33 (m, 2H); 13C NMR (150 MHz, CD_3OD) δ 174.6, 172.5, 171.7, 171.4, 166.3, 159.3, 152.7, 142.7, 103.1, 95.8, 90.3, 85.0 84.9, 75.9, 73.4, 70.7, 69.9, 69.6, 65.8, 62.9, 58.2, 56.0, 55.3, 55.2, 53.9, 53.1, 53.1, 22.6, 21.1, 20.7, 20.7, 18.1; HRMS (ESI) calcd. for $C_{24}H_{33}N_6O_{20}P2$ (M-H$^+$) 787.1225 found 787.1227 *m/z*.

Uridine 5'-diphospho-2-((*S*)-azidopopionamido)-2-deoxy-α-D-glucopyranoside sodium salt (SI-8)

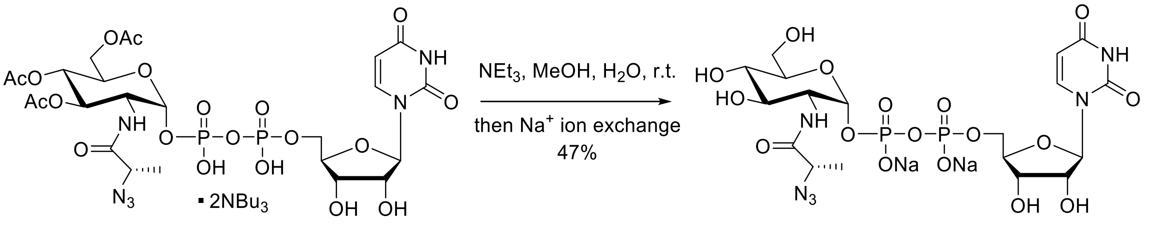

SI-7 UDP-GlcNAzMe(*S*) **SI-8**

To a stirred solution of triester **SI-7** (8.3 mg, 7.2 µmol) in MeOH/water (5:2, 1.5 mL) was added triethylamine (300 µL). The reaction was stirred at room temperature for 16 h and concentrated. The residue was lyophilized repeatedly, passed through a short (3 g resin) ion exchange column (Dowex 50W X8 Na$^+$ form, Sigma Aldrich), and concentrated. The residue was purified by reverse-phase solid-phase extraction (HyperSep^{TM} C18, Thermo Fisher Scientific, Waltham, USA) and lyophilized to give UDP-GlcNAzMe(*S*) **SI-8** as the disodium salt (2.4 mg, 3.4 µmol, 47%) as a white solid. ^{1}H NMR (600 MHz, D_2O) δ 7.97 (d, *J* = 8.1 Hz, 1H), 6.03 – 5.86 (m, 2H), 5.56 (dd, *J* = 7.0, 3.3 Hz, 1H), 4.44 – 4.35 (m, 2H), 4.34 – 4.23 (m, 3H), 4.22 – 4.15 (m, 1H), 4.05 (m, 1H), 3.96 (m, 1H), 3.92 – 3.80 (m, 3H), 3.57 (dd, *J* = 10.1, 9.2 Hz, 1H), 1.50 (d, *J* = 7.0 Hz, 3H); ^{13}C NMR (150 MHz, D_2O) δ 174.2, 141.4, 102.6, 94.5, 94.4, 88.4, 83.1, 83.0, 73.7, 73.0, 70.8, 69.6, 69.5, 64.9, 60.2, 58.1, 53.6, 53.6, 16.8; HRMS (ESI) calcd. for $C_9H_{15}N_4O_5^+$ (oxacarbenium ion) 259.1042 found 259.1046 *m/z*.

2-[3-Azidopropionamido]-2-deoxy-1,3,4,6-tetra-*O*-acetyl-D-glucopyranose (SI-10)

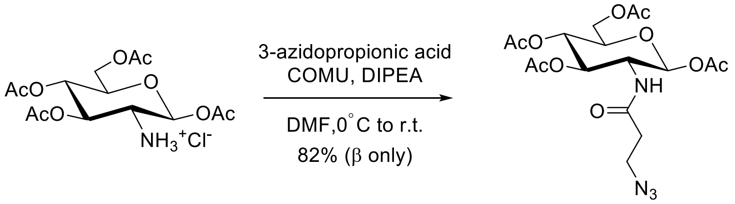

SI-9 **SI-10**

A mixture of peracetylated glucosamine hydrochloride **SI-9** (31) (384 mg, 1 mmol), 3-azidopropionic acid (93 µl, 1 mmol) and DIPEA (0.522 ml, 3 mmol) in DMF (8 ml) was cooled to 0 °C. COMU (856 mg, 2 mmol) was added and the reaction mixture stirred at 0 °C for 1 h. The solution was warmed to room temperature and stirred for another 3 h. The mixture was diluted with EtOAc (100 ml) and

the organic layer was washed with 1 N aq. HCl (2x50 mL), sat. $NaHCO_3$ (2x50 ml) and brine, dried over $MgSO_4$, filtered and concentrated. The residue was purified by medium-pressure flash chromatography (25g SNAP-KP-SIL; A: cyclohexane, B: EtOAc; 20 CV linear gradient from 30% to 70%, then 5 CV from 70% to 100%B) to give tetraacetate **SI-10** (366 mg, 0.82 mmol, 82% β-anomer only) as a clear oil. ^{1}H NMR (400 MHz, $CDCl_3$) δ 5.72 (d, *J* = 8.8 Hz, 1H), 5.66 (d, *J* = 9.4 Hz, 1H), 5.20 – 5.11 (m, 2H), 4.36 – 4.24 (m, 2H), 4.13 (dd, *J* = 12.5, 2.3 Hz, 1H), 3.81 (m, 1H), 3.62 – 3.55 (m, 2H), 2.34 (m, 2H), 2.12 (s, 3H), 2.09 (s, 3H), 2.05 (d, *J* = 3.7 Hz, 6H); ^{13}C NMR (100 MHz, $CDCl_3$) δ 171.3, 170.7, 170.0, 169.5, 169.2, 92.5, 73.0, 72.4, 67.7, 61.6, 53.2, 47.1, 35.9, 20.8, 20.7, 20.6; LRMS (ESI) calcd. for $C_{17}H_{24}N_4O_{10}$ (M-H$^+$) 444.4 found 443.2 *m/z*.

2-[3-Azidopropionamido]-2-deoxy-3,4,6-tri-*O*-acetyl-D-glucopyranose (SI-11)

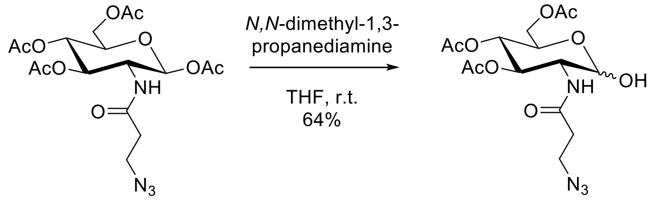

SI-10 SI-11

To a stirred solution of tetraacetate **SI-10** (151 mg, 0.34 mmol) in THF (1.65 mL) was added 3-(dimethylamino)-1-propylamine (0.123 mL, 1.02 mmol). The reaction was stirred at room temperature for 2 h, diluted with CH_2Cl_2 (25 mL), washed with 1 N aq. HCl (2x10 mL) and brine (10 mL). The organic phase was dried over $MgSO_4$, filtered and concentrated. The residue was purified by medium-pressure flash chromatography (10 g SNAP; A: cyclohexane, B: EtOAc; 20 CV linear gradient from 30% to 100% B, then 5 CV 100% B) to give tetraacetate **SI-11** (88 mg, 0.22 mmol, 64%, >19:1 α:β) as a clear oil. ^{1}H NMR (400 MHz, $CDCl_3$) δ 5.91 (d, *J* = 9.3 Hz, 1H), 5.35 – 5.28 (m, 2H), 5.18 – 5.11 (m, 1H), 4.35 (m, 1H), 4.26 – 4.10 (m, 3H), 3.59 (t, *J* = 6.3 Hz, 2H), 3.01 (dd, *J* = 3.8, 1.6 Hz, 1H), 2.39 (td, *J* = 6.2, 4.0 Hz, 2H), 2.10 (s, 3H), 2.04 (d, *J* = 2.3 Hz, 6H). ^{13}C NMR (100 MHz, $CDCl_3$) δ 171.5, 171.0, 170.1, 169.4, 91.6, 70.8, 68.2, 67.7, 62.1, 52.3, 47.2, 35.7, 20.8, 20.7, 20.6; LRMS (ESI) calcd. for $C_{15}H_{22}N_4O_9$ (M-H$^+$) 402.14 found 401.21 *m/z*.

Bis-*O*-allyl 2-[3-Azidopropionamido]-2-deoxy-3,4,6-tetra-*O*-acetyl-α-D-glucopyranosyl phosphate (SI-12)

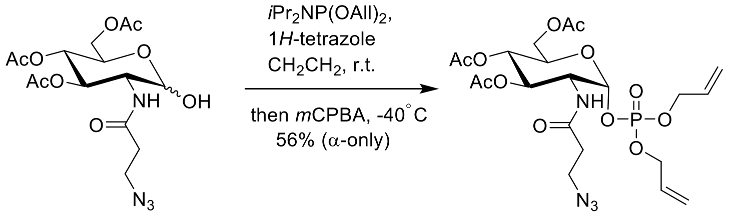

SI-11 SI-12

Lactol **SI-11** (88 mg, 0.22 mmol) and 1*H*-tetrazole (69 mg, 2.2 mL of 0.45M solution in ACN, 1 mmol) were co-evaporated with anhydrous toluene (2 mL), suspended in anhydrous toluene (2 mL) and sonicated for 1 h at room temperature in a bath sonicator. The solvent was evaporated and the residue dissolved in anhydrous CH_2Cl_2 (3.2 mL). The stirred solution was cooled to 0 °C and diallyl *N*,*N*-diisopropylphosphoramidite (93 µL, 0.35 mmol) was added. After 20 min, the solution was cooled to -40 °C, and mCPBA (149 mg, 0.66 mmol) was added. After 30 min, the reaction was quenched with 1 M aq. Na_2SO_3 (8 mL), and warmed to room temperature. The mixture was diluted with CH_2Cl_2 (10 mL) and the layers were separated. The organic phase was washed with sat. aq. $NaHCO_3$ (10 mL) and the combined aqueous phase was re-extracted with CH_2Cl_2 (2x20 mL). The combined organic phase was washed with brine (20 mL), dried over $MgSO_4$, and concentrated. The residue was purified by medium-pressure flash chromatography (10g SNAP-KP-SIL; A: cyclohexane, B: EtOAc; 25 CV linear gradient from 30% to 95%, then 5 CV from 95%B) to give phosphate **SI-12** (70 mg, 0.12 mmol, 56%, α-anomer only) as a clear oil. ^{1}H NMR (400 MHz, $CDCl_3$) δ 6.41 (d, *J* = 9.1 Hz, 1H), 6.04 – 5.84 (m, 2H), 5.67 (dd, *J* = 6.3, 3.3 Hz, 1H), 5.47 – 5.12 (m, 6H), 4.58 (m, 4H), 4.43 (m, 1H), 4.28 – 4.16 (m, 2H), 4.14 – 4.05 (m, 1H), 3.56 (m, 2H), 2.47 – 2.32 (m,

2H), 2.06 (s, 3H), 2.01 (d, *J* = 2.3 Hz, 6H). ^{13}C NMR (100 MHz, $CDCl_3$) δ 171.2, 170.6, 170.2, 169.2, 132.2, 132.1, 132.0, 131.9, 119.1, 96.0, 70.0, 69.8, 68.9, 68.8, 68.8, 68.7, 67.5, 61.4, 51.9, 51.8, 47.0, 35.4, 29.7, 20.7, 20.6, 20.5; LRMS (ESI) calcd. for $C_{21}H_{31}N_4O_{12}P$ (M-H$^+$) 562.17 found 561.18 *m/z*.

Uridine 5'-diphospho-2-(3-azidopropionamido)-2-deoxy-α-D-glucopyranoside ammonium salt (SI-13)

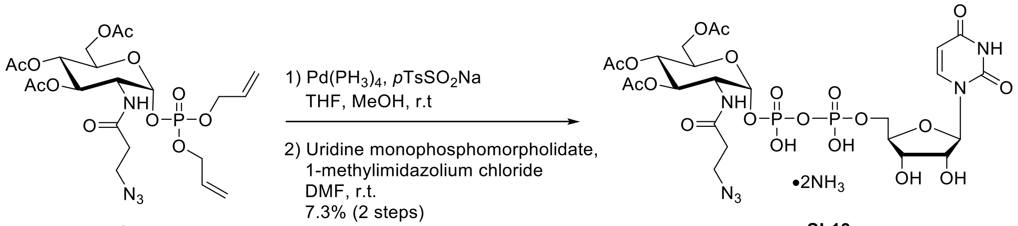

To a stirred solution of diallyl phosphotriester **SI-12** (66 mg, 117 µmol) in THF/MeOH (1:1, 2.4 mL) were added tetrakis(triphenylphosphine)palladium (14 mg, 12 µmol) and sodium *para*-toluoenesulfinate (84 mg, 468 µmol). The mixture was stirred at room temperature for 16 h, and the solvents were evaporated. The residue was co-evaporated with anhydrous toluene (2x10 mL) and dissolved in DMF (2.4 mL). Uridine monophosphomorpholidate (128 mg, 187 µmol) and 1-methylimidazolium chloride (75 mg, 0.64 µmol) were added and the reaction was stirred at room temperature for 16 h. Additional uridine monophosphomorpholidate (128 mg, 187 µmol) and 1-methylimidazolium chloride (75 mg, 0.64 µmol) were added to the mixture and left to react for 16 h. The mixture was concentrated and purified by medium-pressure flash chromatography (30 g SNAP C18 column; A: 10 mM ammonium acetate, B: MeOH; 5 CV 100% A; 20 CV linear gradient to 100% B, then 5 CV 100% B) and then by preparative HPLC (ZORBAX 300SB-C8; A: 10 mM Ammonium Acetate, B: MeOH; linear gradient from 0 to 60% B over 40 min). The fractions were concentrated and lyophilized to give pyrophosphate **SI-13** as the ammonium salt (7 mg, 8.5 µmol, 7.3% over two steps) as a white foam. ^1H NMR (400 MHz, CD_3OD) δ 8.09 (d, *J* = 8.1 Hz, 1H), 5.97 (d, *J* = 4.5 Hz, 1H), 5.86 (d, *J* = 8.1 Hz, 1H), 5.63 (dd, *J* = 7.3, 3.3 Hz, 1H), 5.31 (dd, *J* = 10.6, 9.4 Hz, 1H), 5.14 (dd, *J* = 10.2, 9.4 Hz, 1H), 4.45 – 4.36 (m, 3H), 4.35 (m, 1H); 4.31 (m, 2H), 4.22 – 4.16 (m, 2H), 3.66 – 3.49 (m, 2H), 2.69 – 2.57 (m, 2H), 2.07 (s, 3H), 2.02 (s, 3H), 1.97 (s, 3H). ^{13}C NMR (100 MHz, CD_3OD) δ 172.3, 171.1, 170.6, 170.0, 164.9, 151.3, 141.3, 101.7, 94.5, 88.9, 83.6, 81.4, 74.4, 71.7, 69.5, 68.5, 68.3, 64.6, 61.5, 51.7, 39.0, 34.6, 19.3; LRMS (ESI) calcd. for $C_{24}H_{33}N_6O_{20}P_2$ (M-H$^+$) 787.13 found 787.10 *m/z*.

Uridine 5'-diphospho-2-(3-azidopropionamido)-2-deoxy-α-D-glucopyranoside sodium salt SI-14

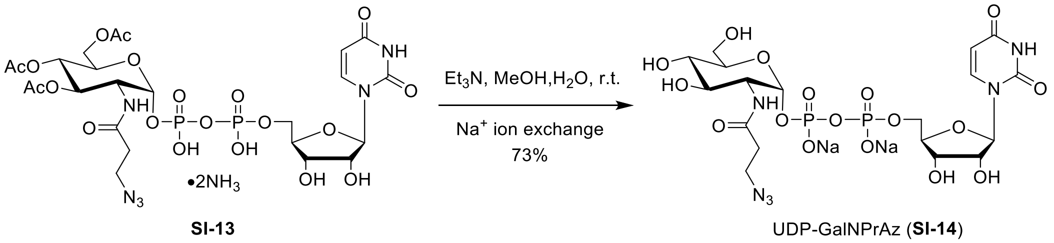

To a stirred solution of triester **SI-13** (7 mg, 8.5 µmol) in MeOH/water (5:2, 1.8 mL) was added triethylamine (375 µL). The reaction was stirred at room temperature for 16 h. The residue was concentrated and lyophilized repeatedly. The residue was passed through a short (4 g resin) ion exchange column of Dowex 50W X8 Na$^+$ form (Serva, Heidelberg, Germany), and concentrated. The residue was purified by reverse-phase solid-phase extraction (Sep-Pak C18, 5g, Waters) and lyophilized to give UDP-GlcNAc analog **SI-14** as the disodium salt (4.4 mg, 6.2 µmol, 73%) as a white solid. ^1H NMR (400 MHz, D_2O) δ 7.80 (d, *J* = 8.1 Hz, 1H), 5.88 – 5.76 (m, 2H), 5.36 (m, 1H), 4.26 – 4.17 (m, 2H), 4.12 (m, 1H), 4.04 (m, 2H), 3.88 (m, 1H), 3.78 (m, 1H), 3.71 (dd, *J* = 12.5, 2.4 Hz, 1H), 3.66 – 3.62 (m, 2H), 3.47 – 3.36 (m, 3H), 2.49 (m, 2H). ^{13}C NMR (100 MHz, D_2O) δ 174.1,

166.4, 151.9, 141.6, 102.6, 94.6, 88.5, 83.1, 73.8, 73.0, 70.9, 69.6, 69.5, 65.0, 60.3, 53.7, 47.1, 34.8; LRMS (ESI) calcd. for $C_{18}H_{28}N_6O_{17}P_2$ ($M-H^+$) 661.10 found 661.10 *m/z*.

^{1}H NMR (400 MHz, acetone-D$_6$)

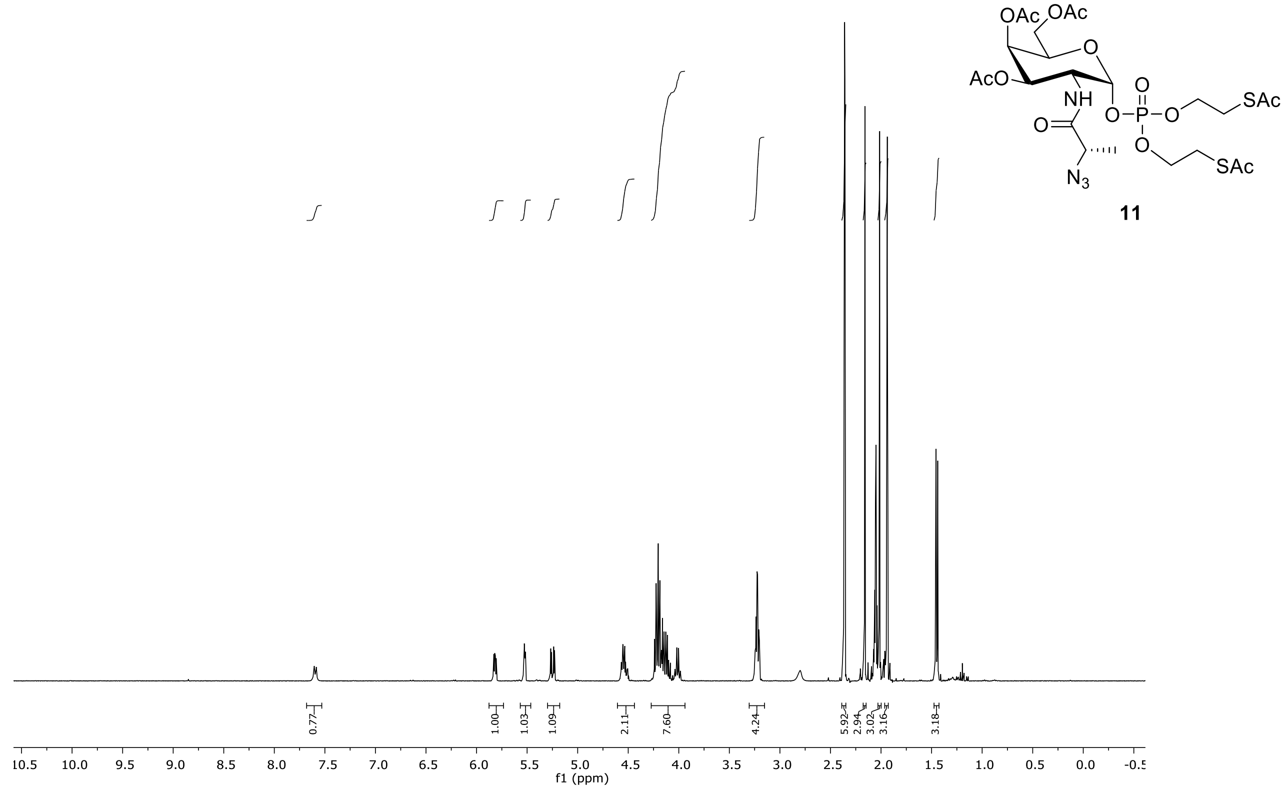

^{1}H NMR (400 MHz, acetone-D$_6$)

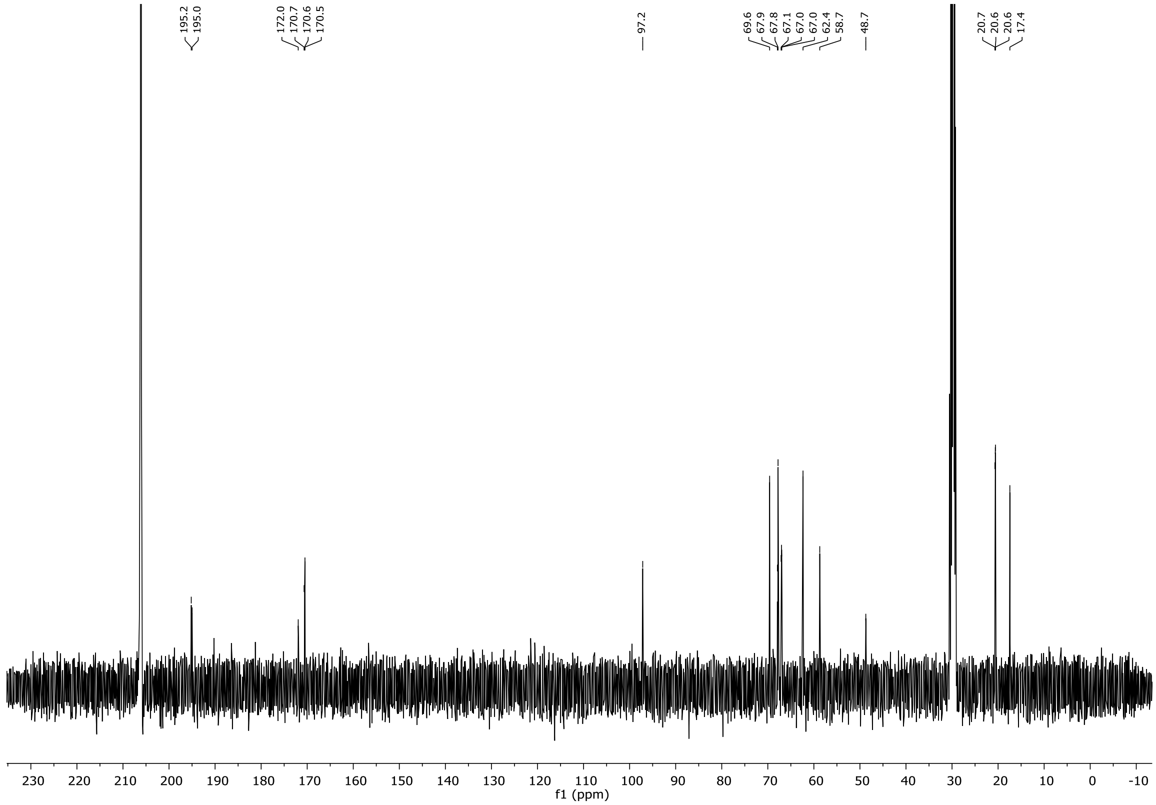

^{1}H NMR (400 MHz, $CDCl_3$)

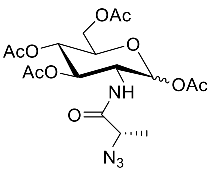

SI-3

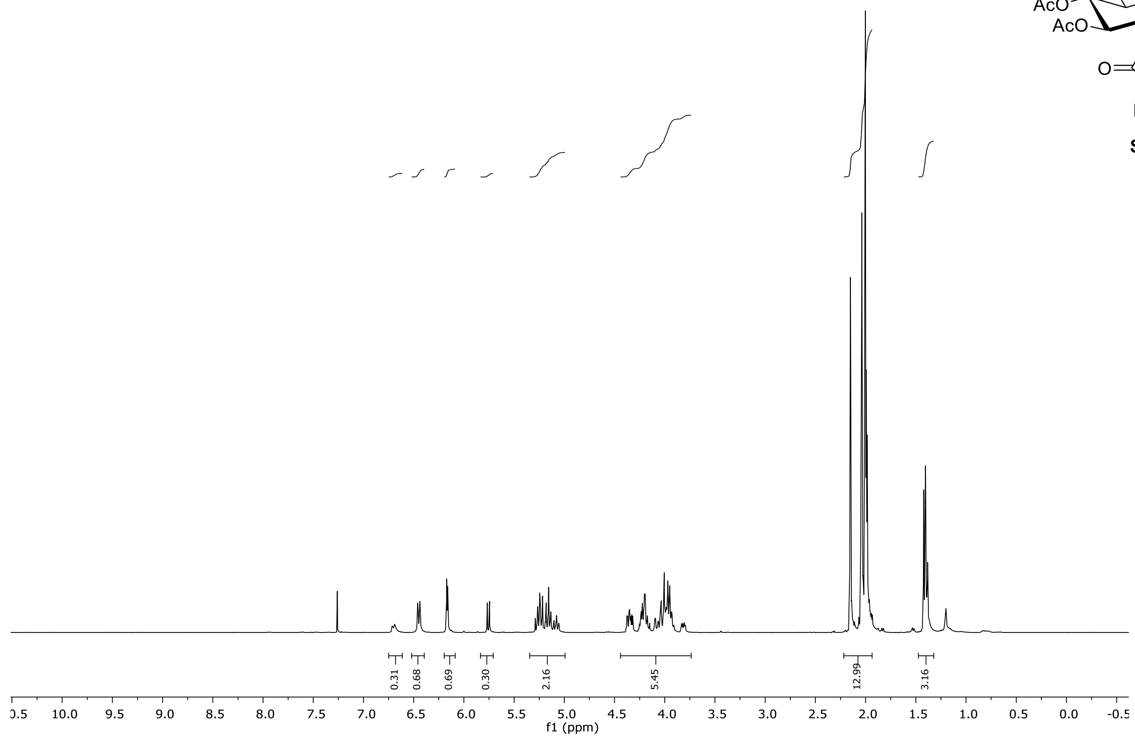

^{13}C NMR (100 MHz, $CDCl_3$)

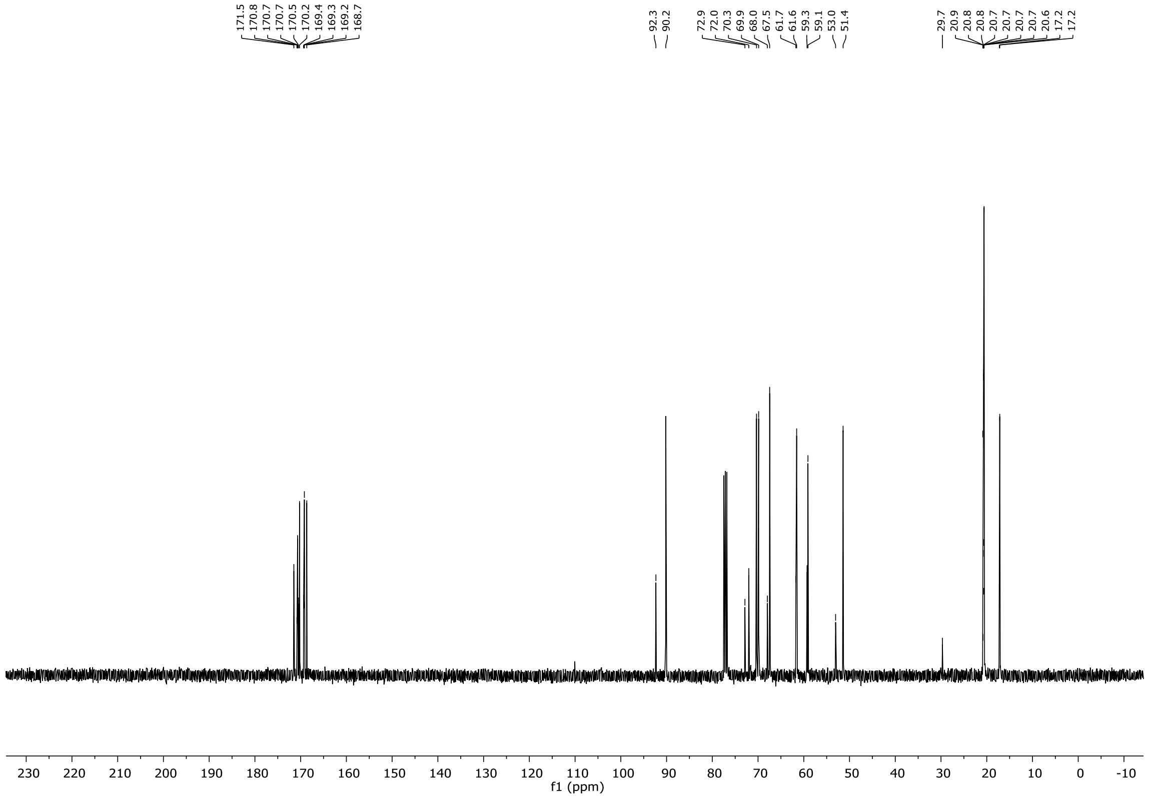

^{1}H NMR (400 MHz, $CDCl_3$)

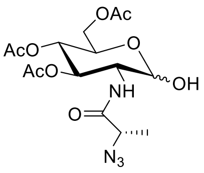

SI-4

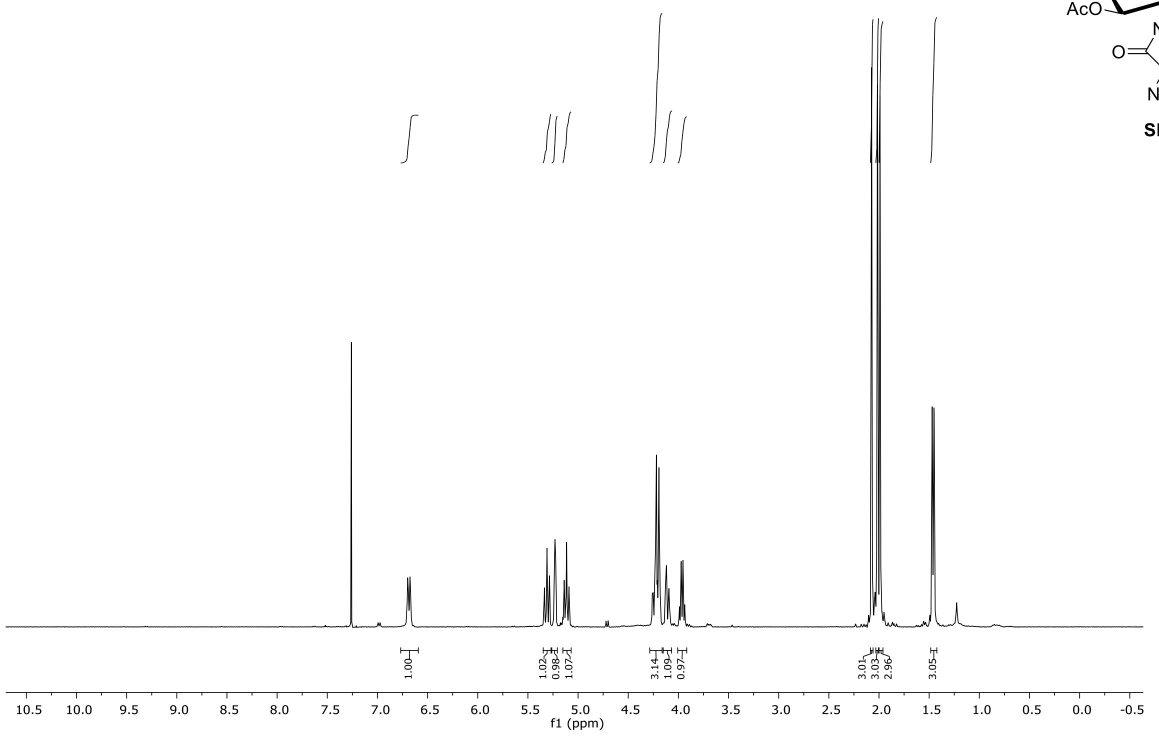

^{13}C NMR (100 MHz, $CDCl_3$)

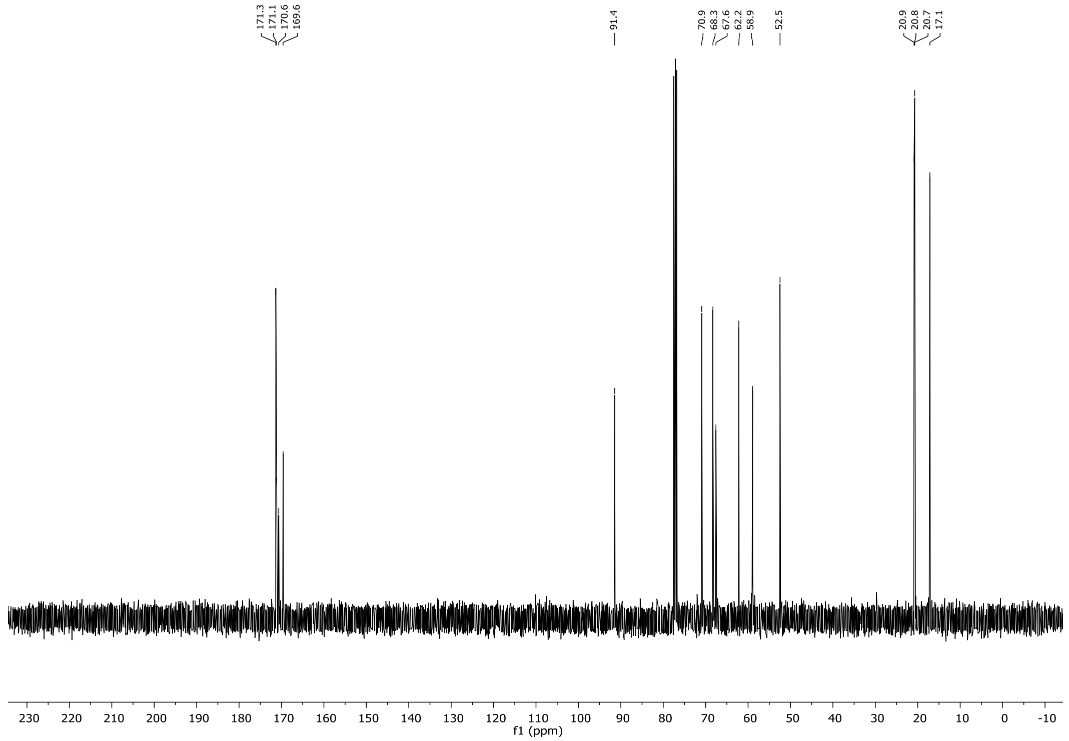

¹H NMR (400 MHz, CDCl₃)

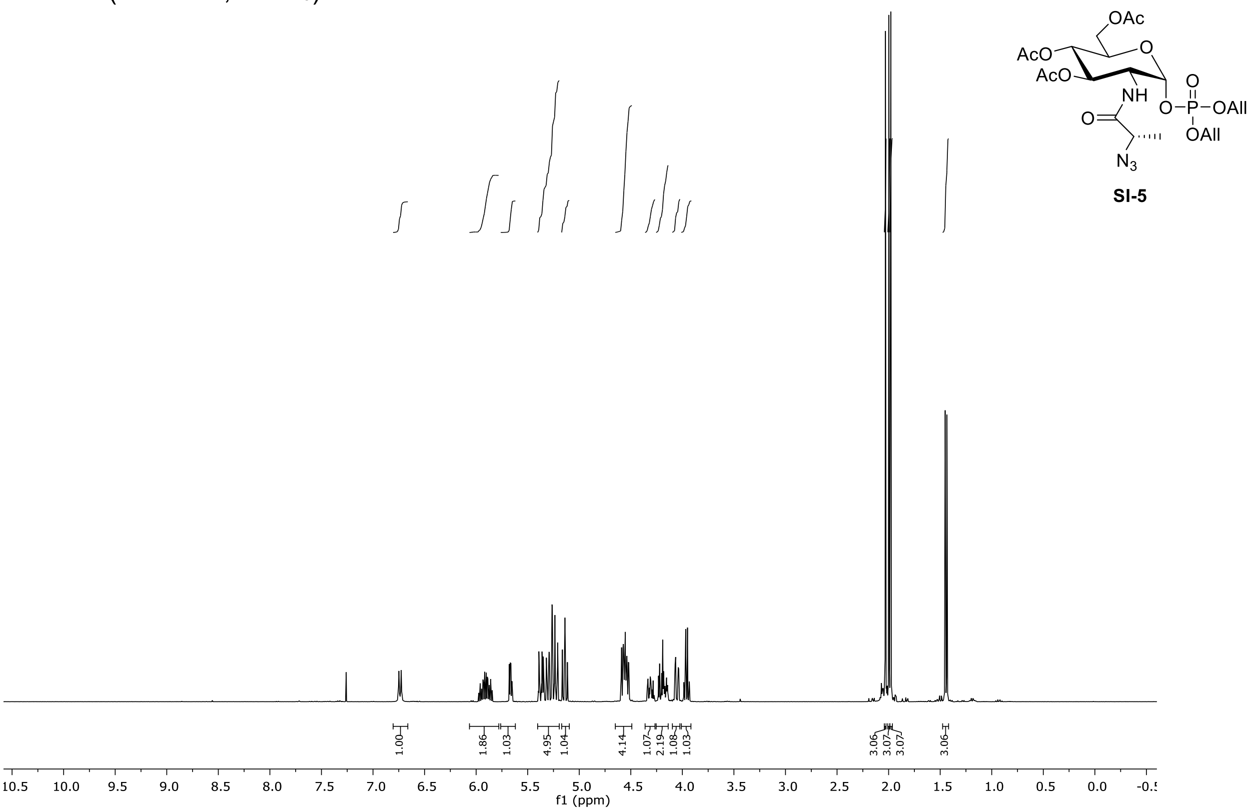

SI-5

¹³C NMR (100 MHz, CDCl₃)

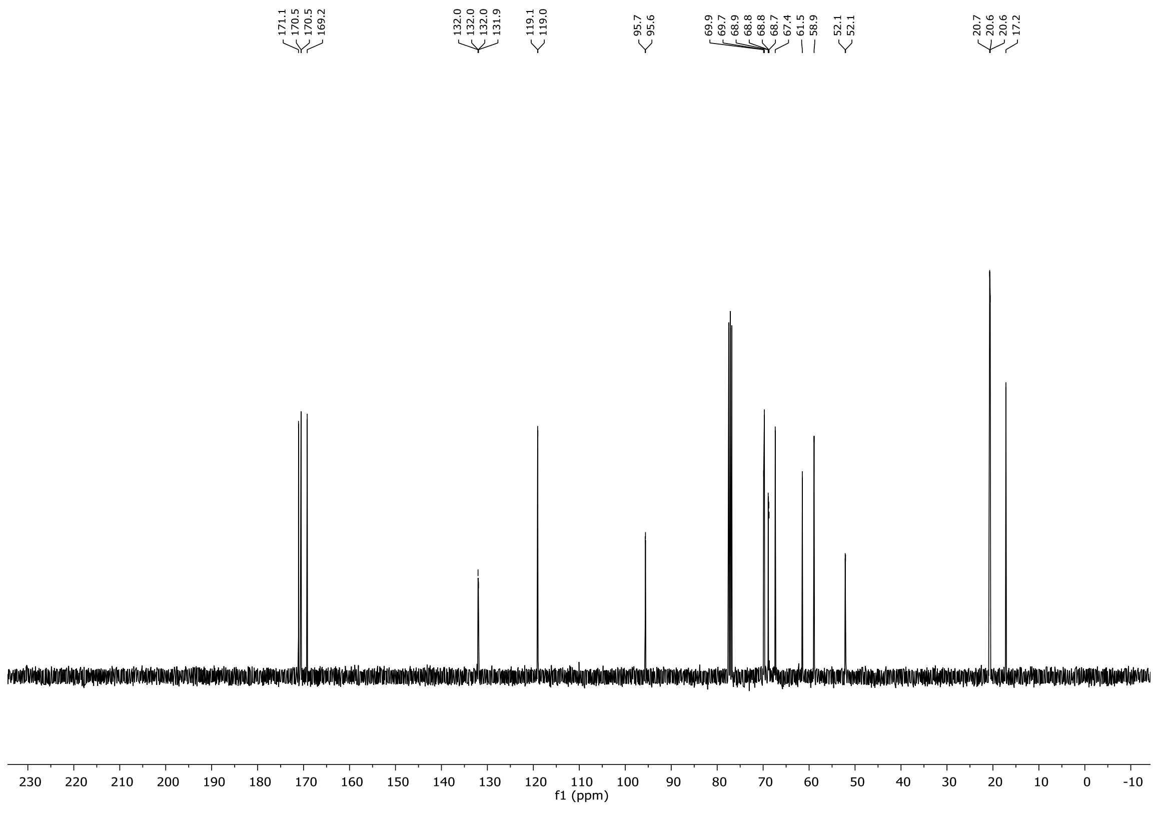

CH-HSQC NMR (400 MHz, CDCl3)

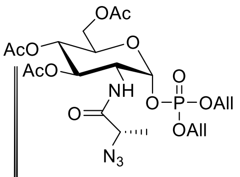

SI-5

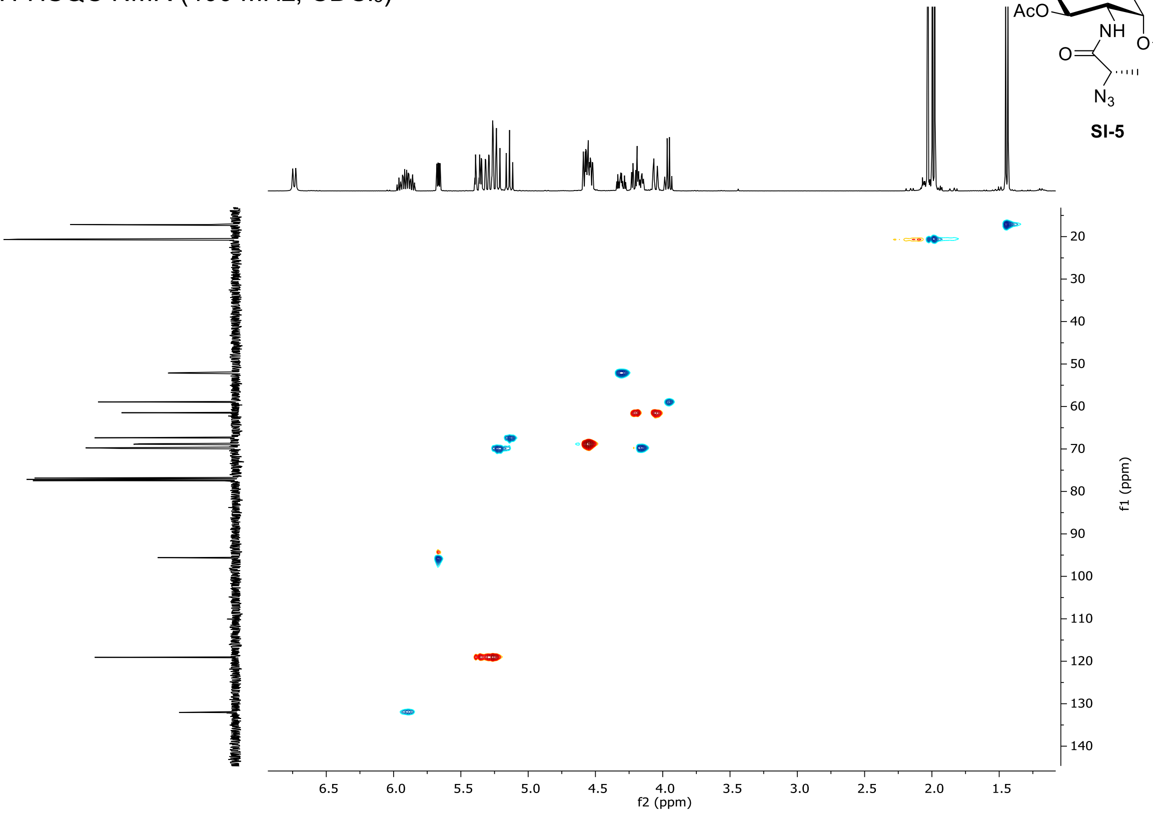

HH-COSY NMR (400 MHz, CDCl3)

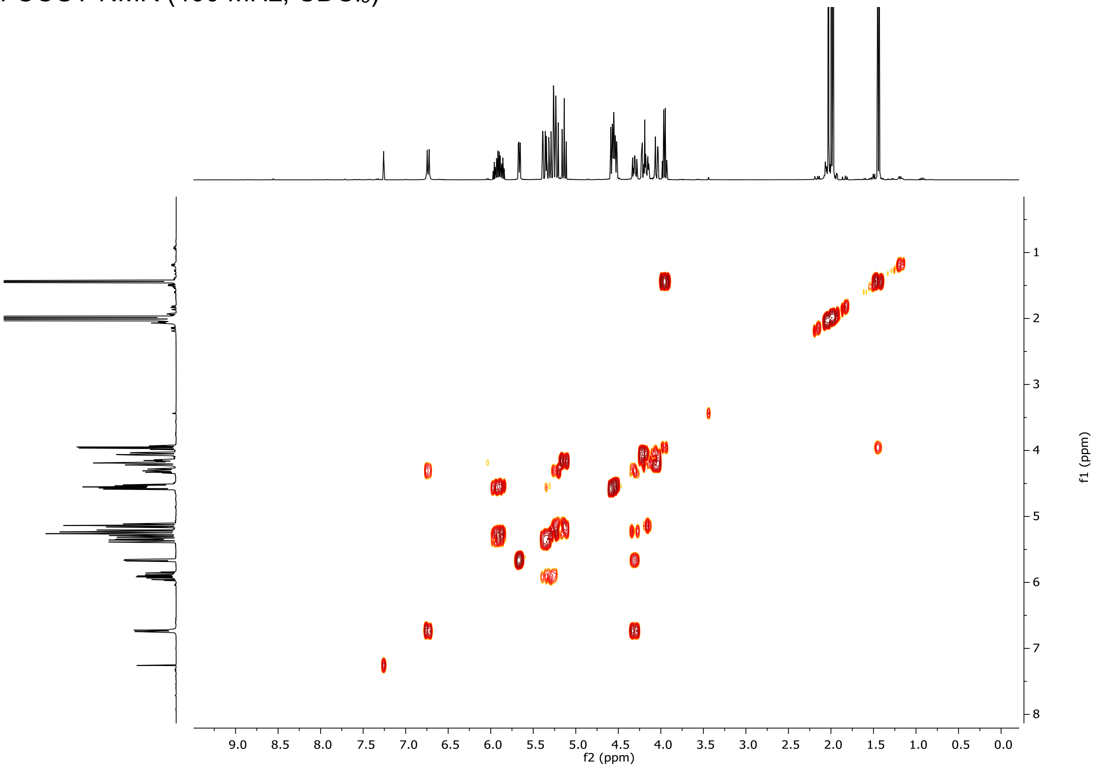

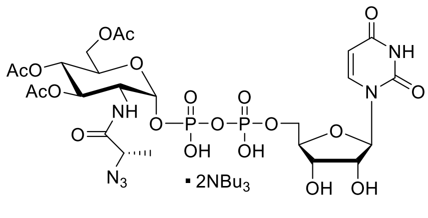

SI-7

^{1}H NMR (600 MHz, CD_3OD)

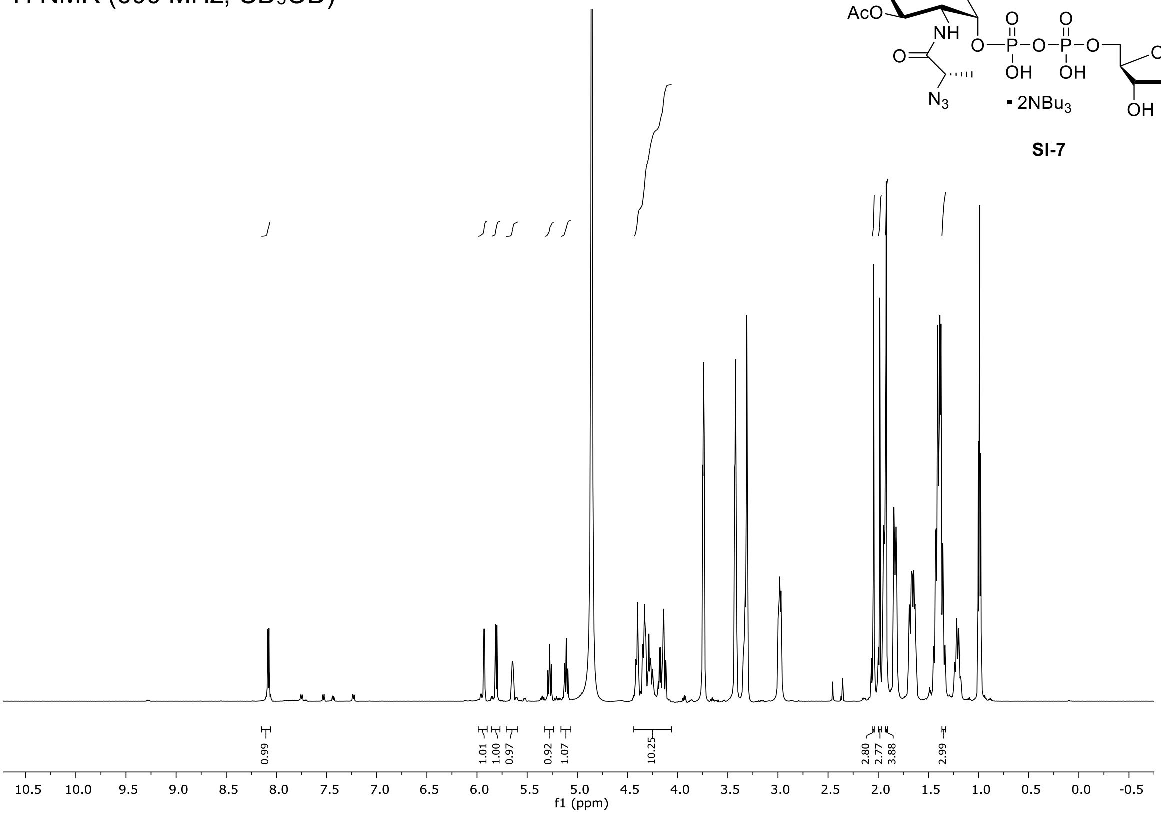

^{13}C NMR (150 MHz, CD_3OD)

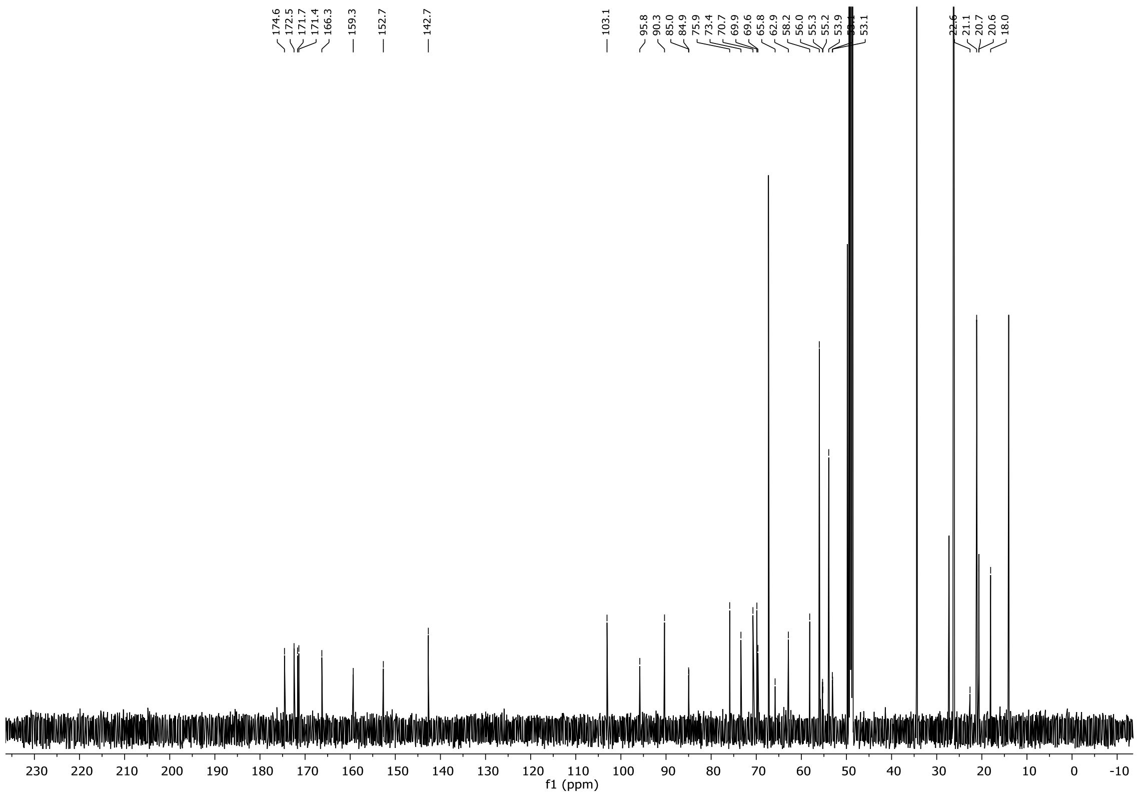

^{1}H NMR (600 MHz, CD_3OD)

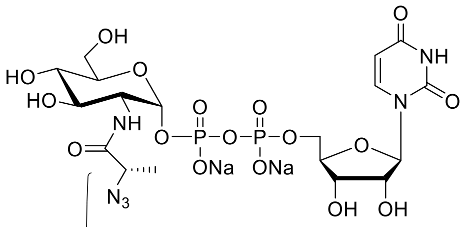

SI-8

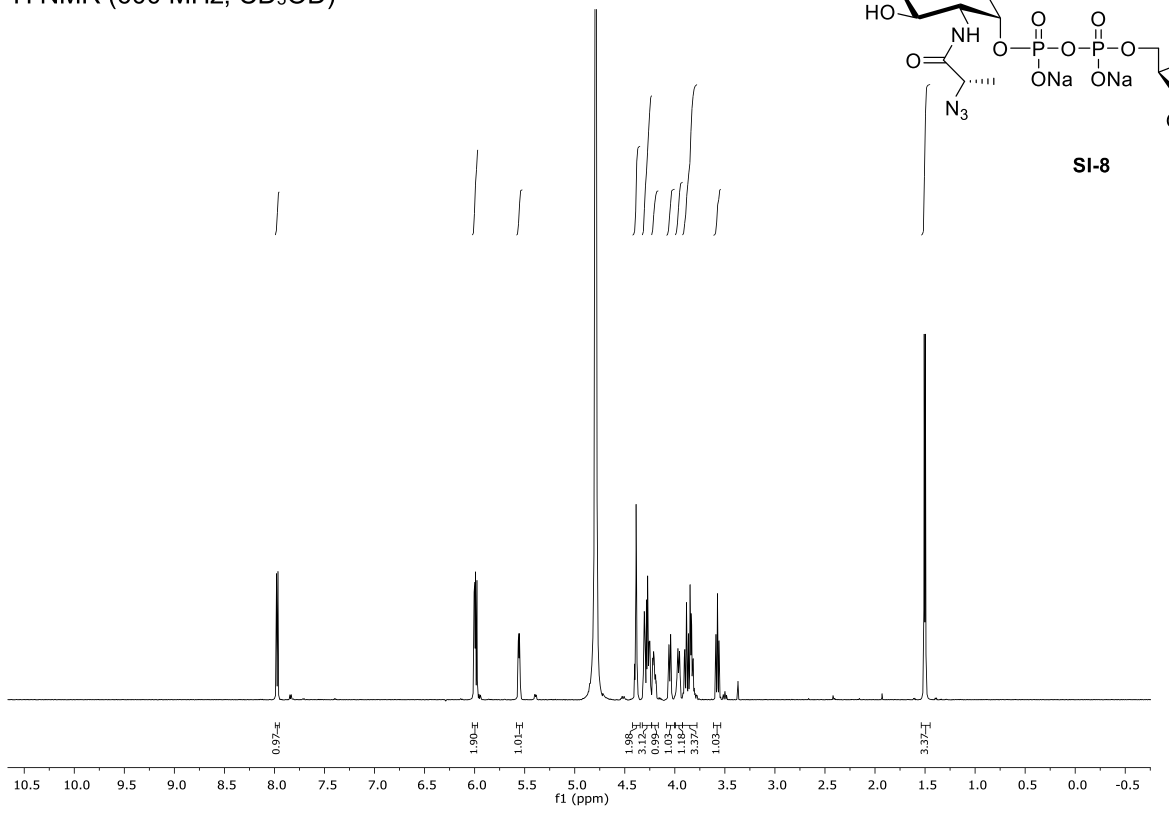

^{13}C NMR (150 MHz, D_2O)

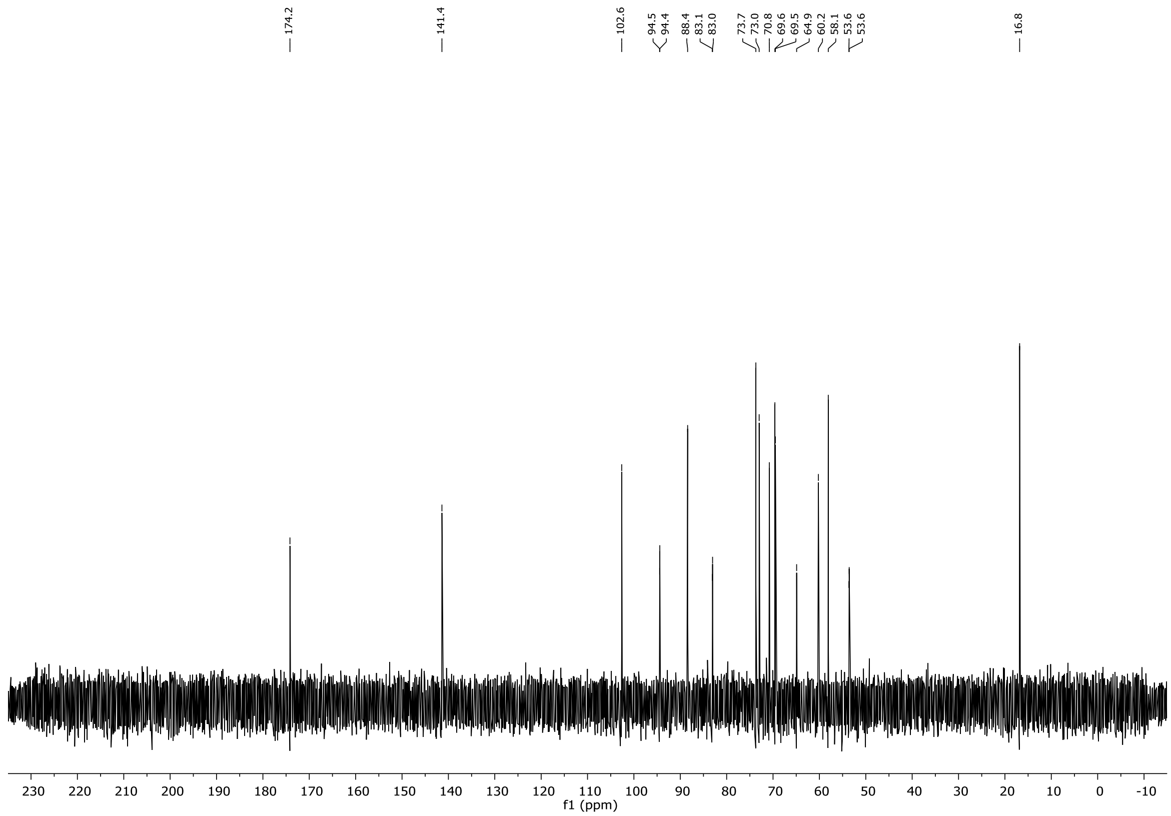

HH-COSY NMR (600 MHz, D_2O)

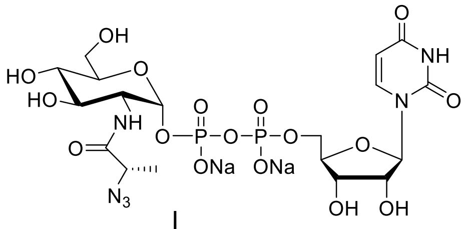

SI-8

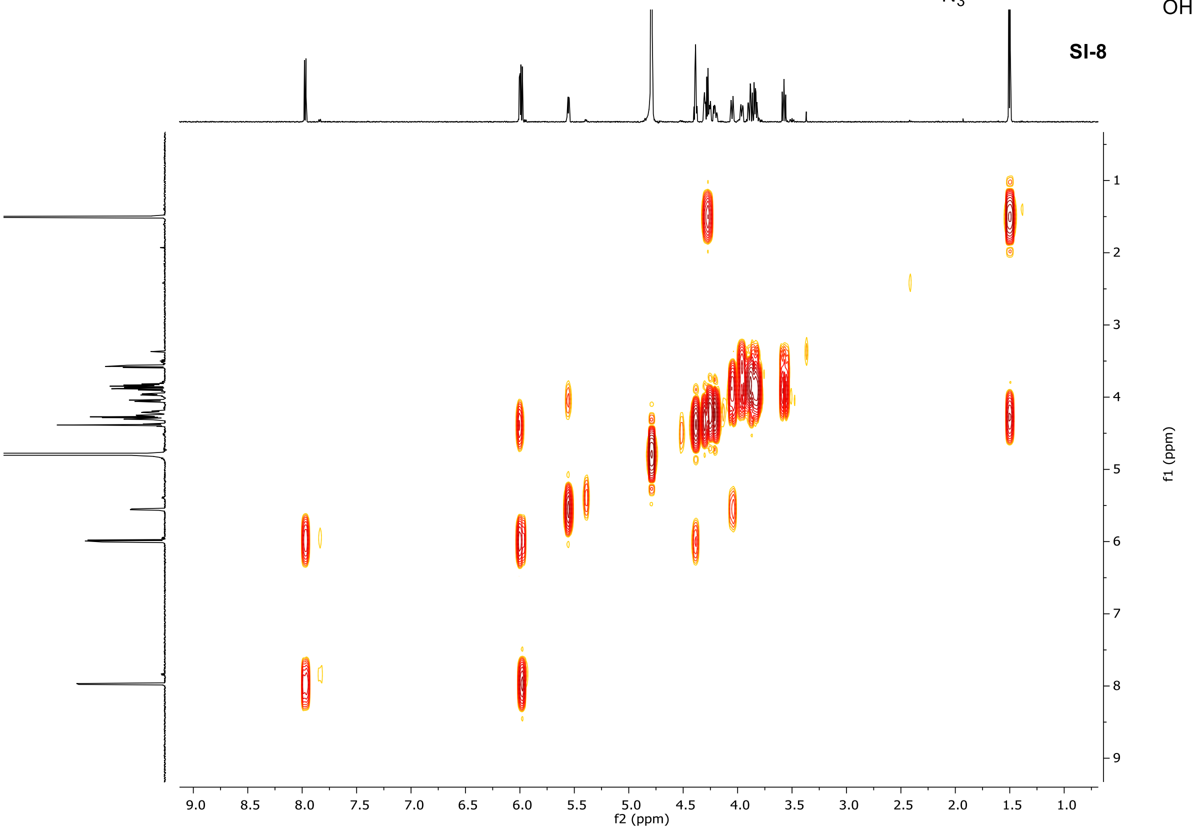

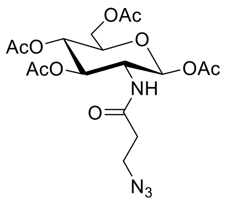

SI-10

^{1}H NMR (400 MHz, $CDCl_3$)

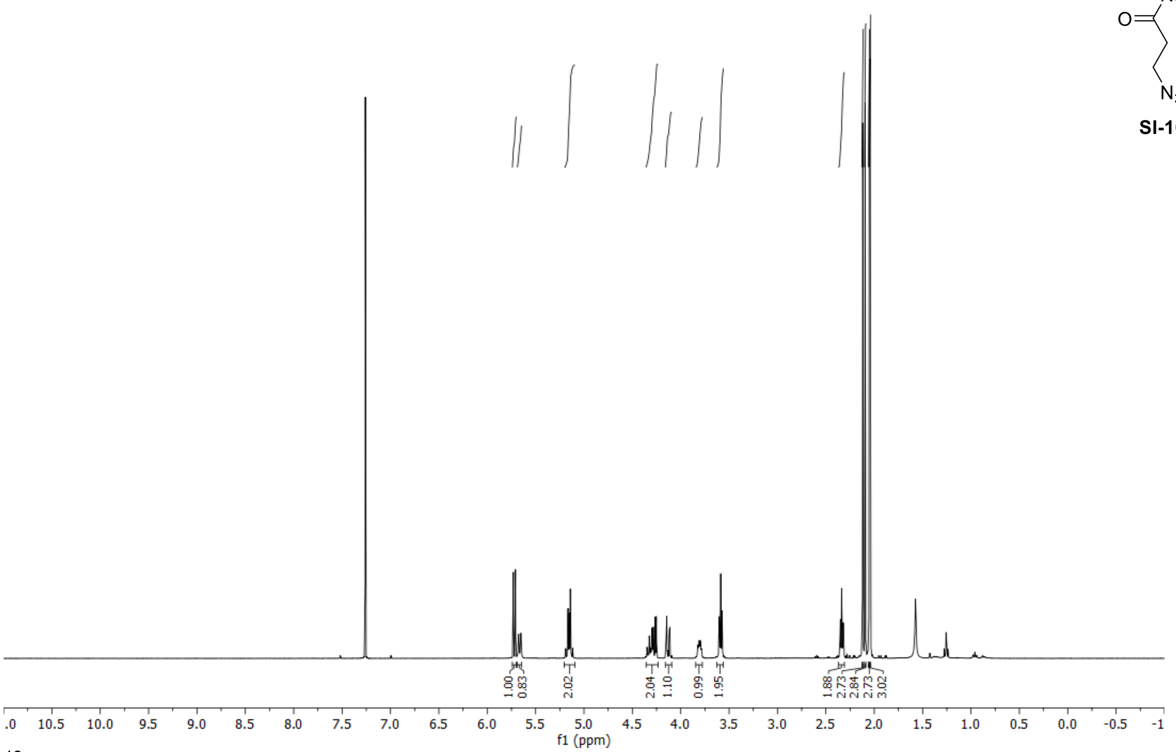

^{13}C NMR (100 MHz, $CDCl_3$)

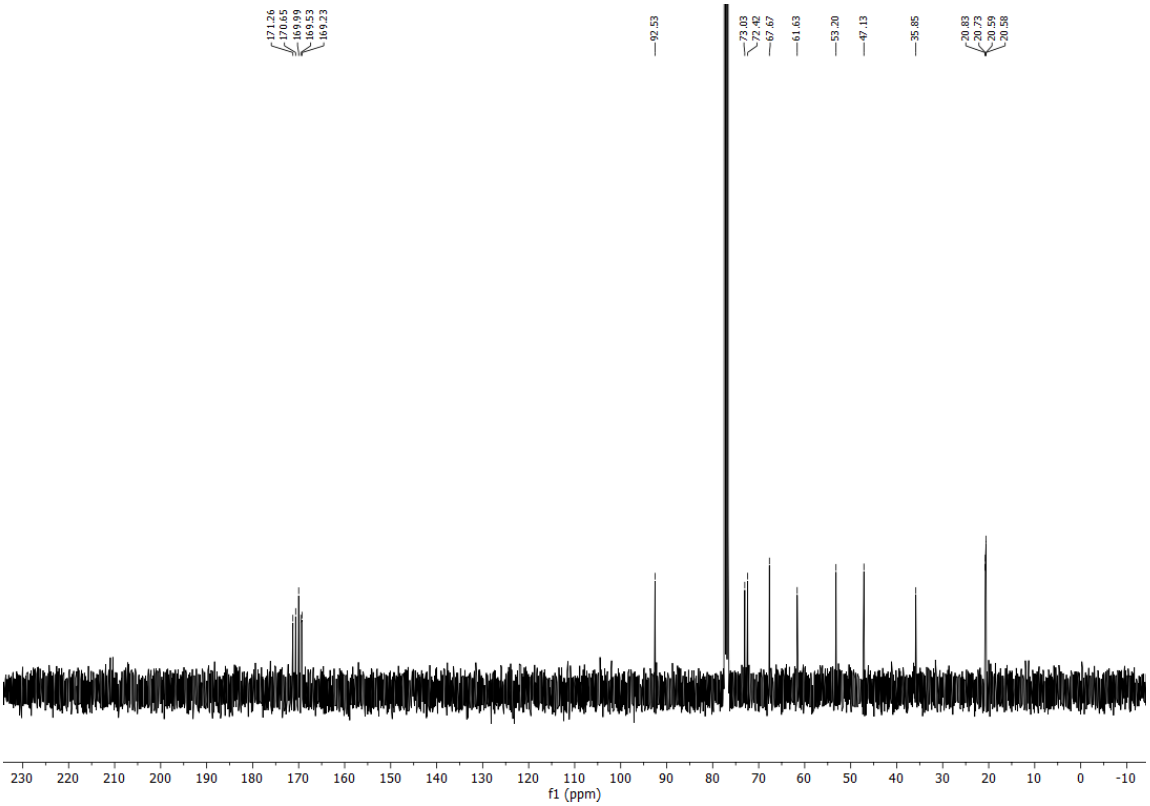

^{1}H NMR (400 MHz, $CDCl_3$)

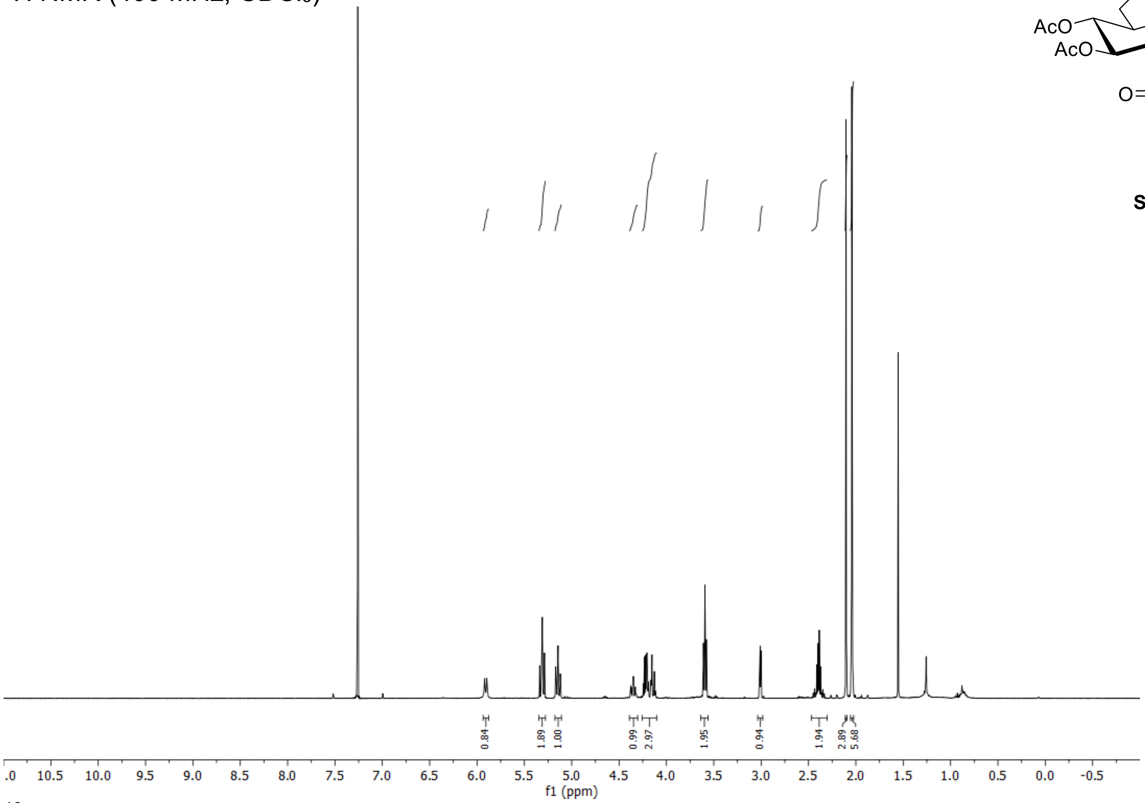

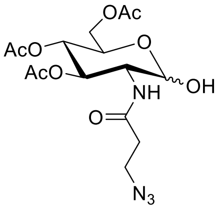

SI-11

^{13}C NMR (100 MHz, $CDCl_3$)

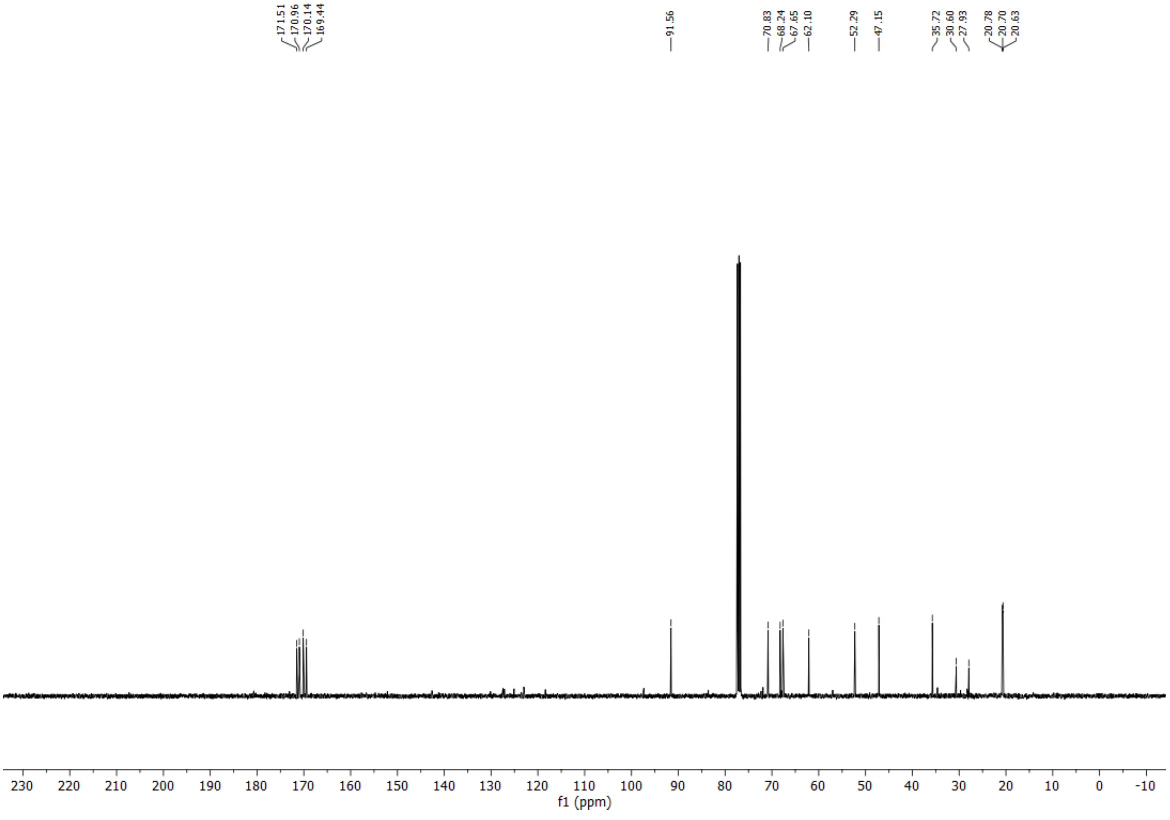

^1H NMR (400 MHz, $CDCl_3$)

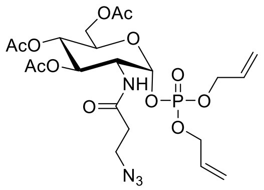

SI-12

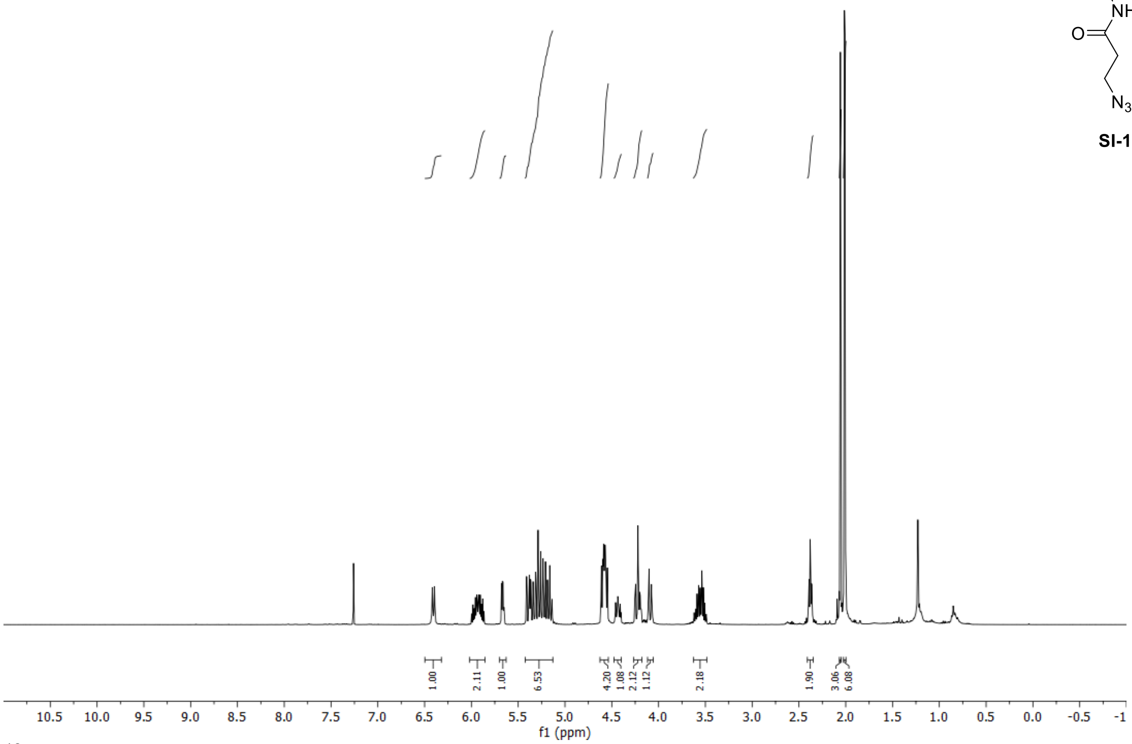

^{13}C NMR (100 MHz, $CDCl_3$)

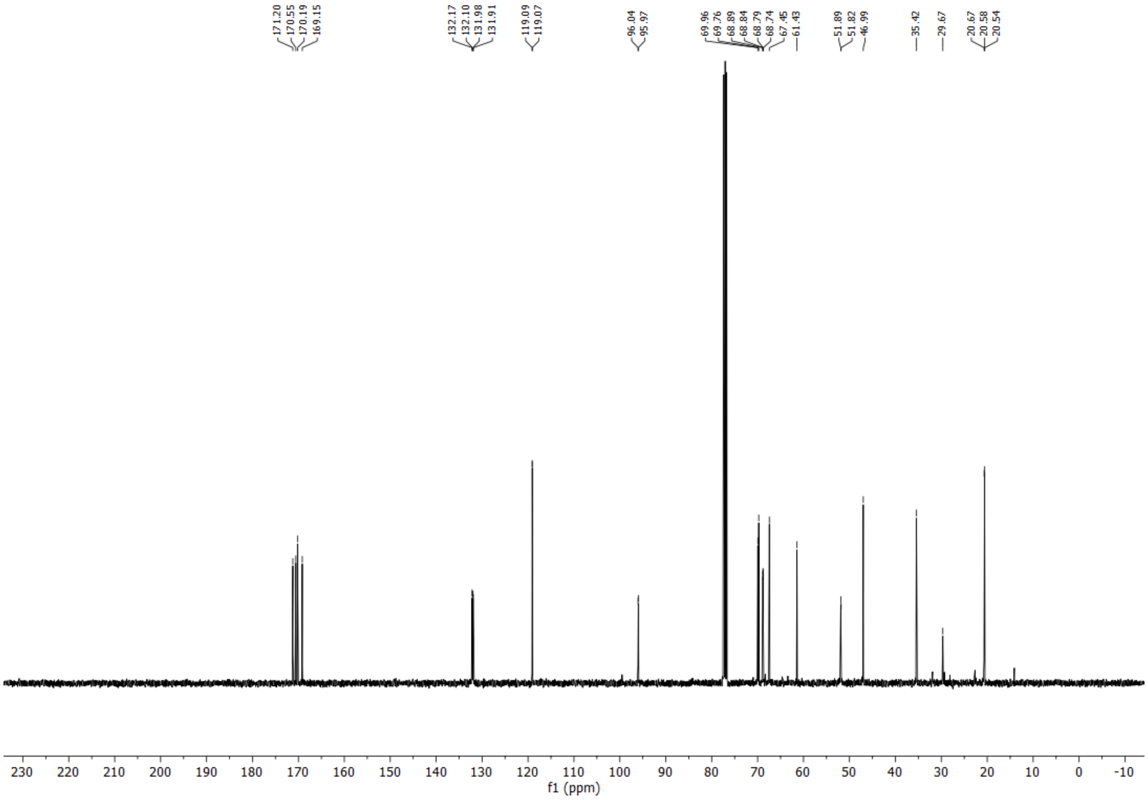

^{1}H NMR (400 MHz, CDCl$_3$)

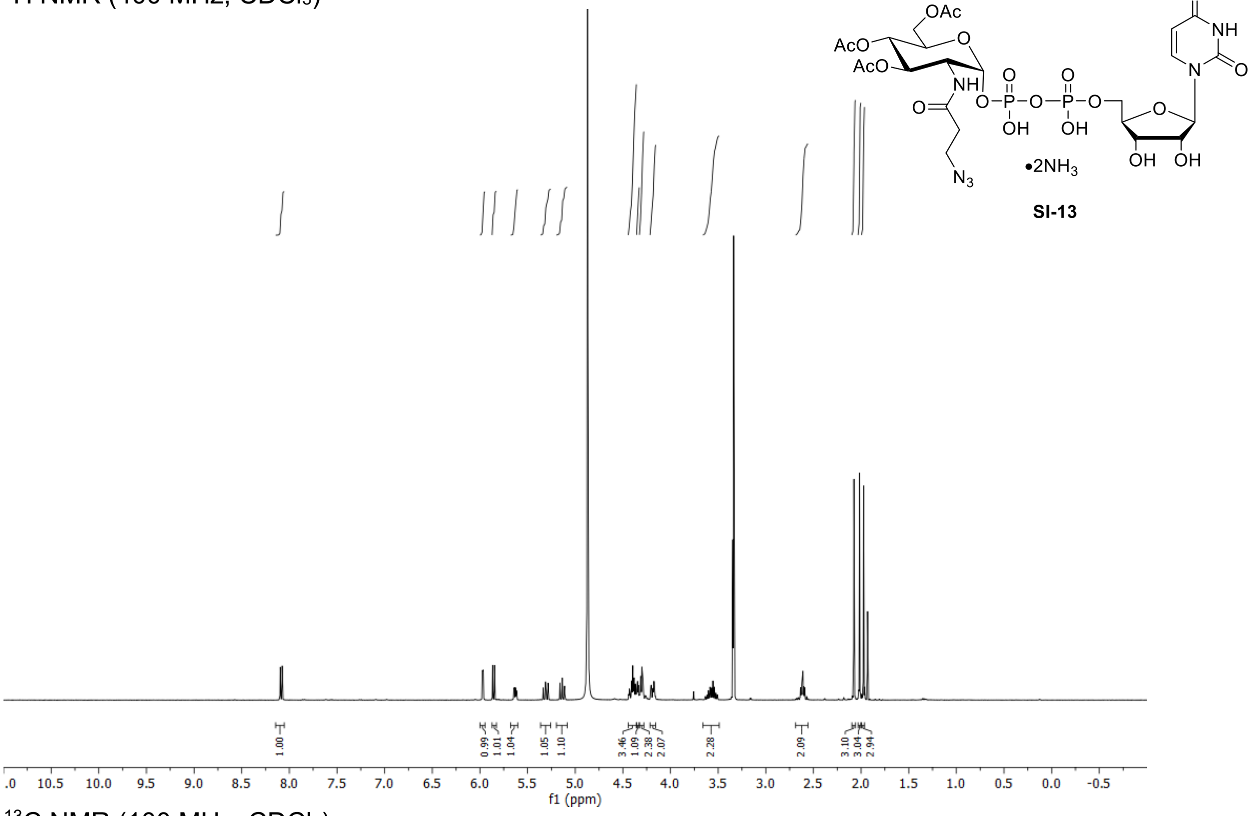

^{13}C NMR (100 MHz, CDCl$_3$)

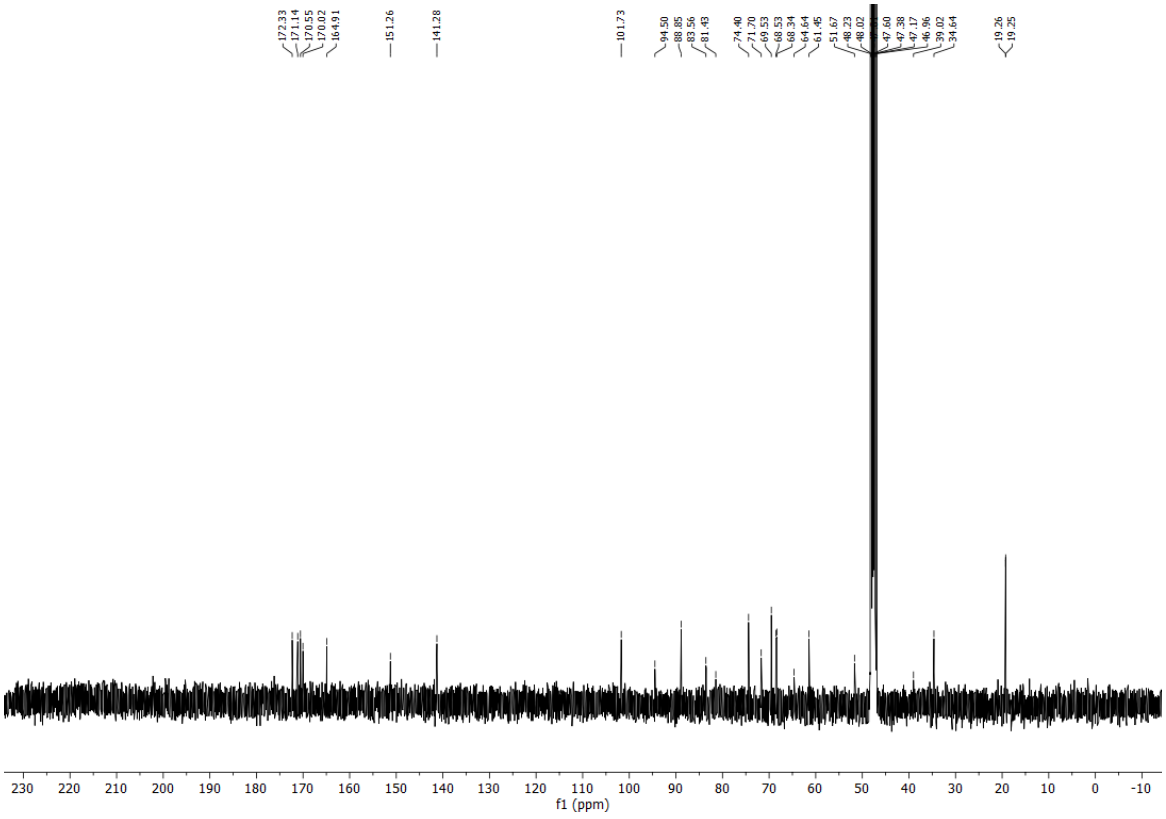

^{1}H NMR (400 MHz, $CDCl_3$)

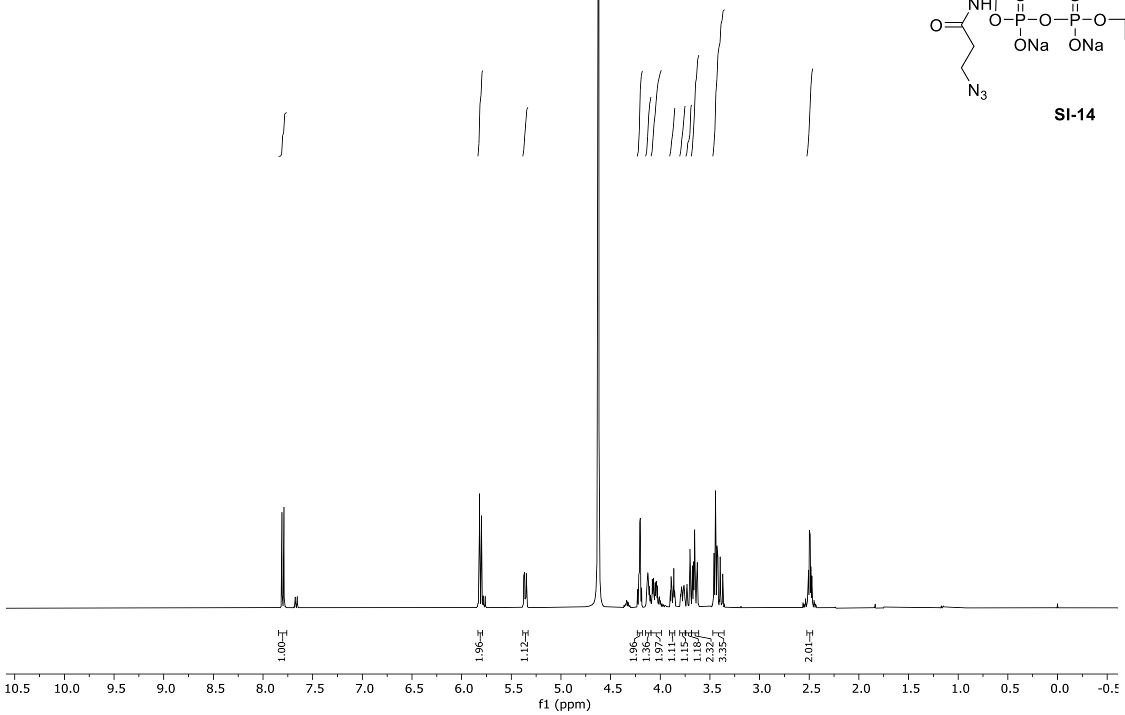

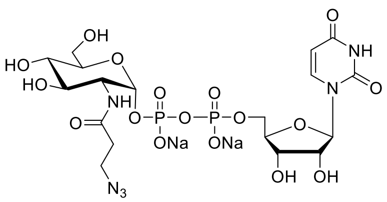

SI-14

^{13}C NMR (100 MHz, $CDCl_3$)

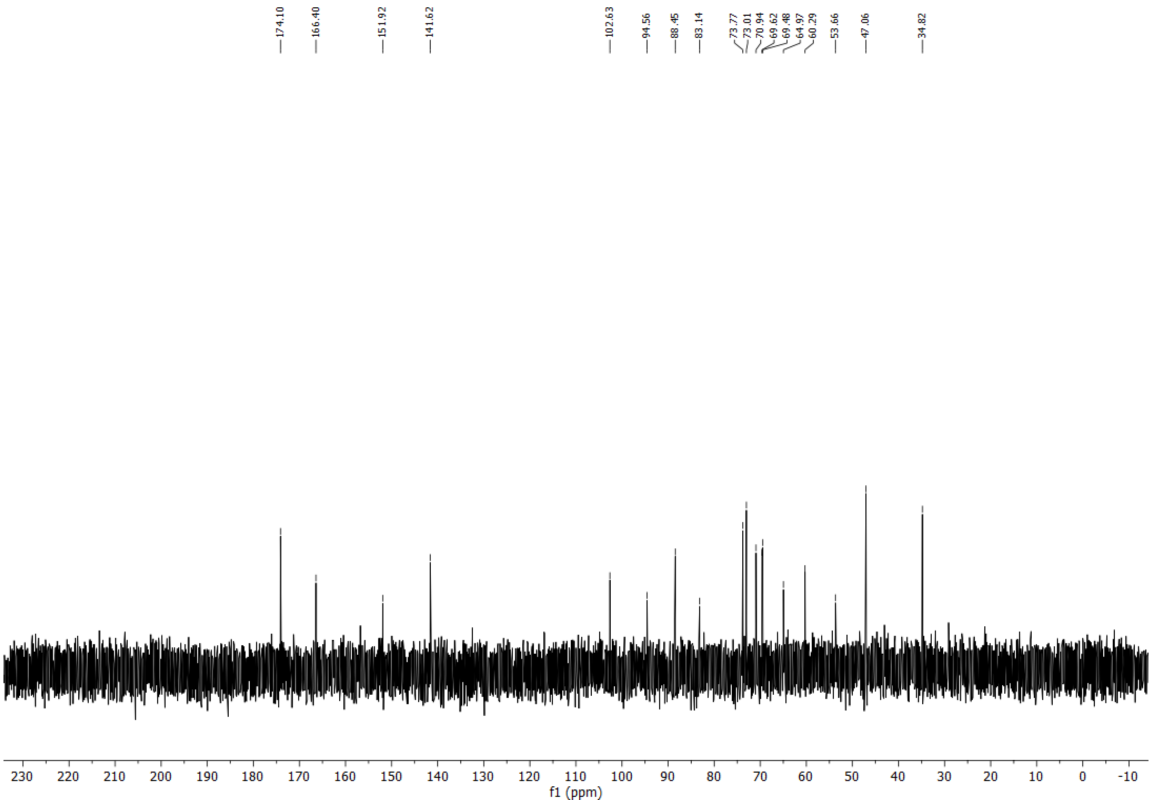

SI References

1. C. Y. Seiler, *et al.*, DNASU plasmid and PSI:Biology-Materials repositories: Resources to accelerate biological research. *Nucleic Acids Res.* **42** (2014).
2. C. Y. Cormier, *et al.*, PSI:Biology-materials repository: A biologist's resource for protein expression plasmids. *J. Struct. Funct. Genomics* **12**, 55–62 (2011).
3. C. Y. Cormier, *et al.*, Protein Structure Initiative Material Repository: An open shared public resource of structural genomics plasmids for the biological community. *Nucleic Acids Res.* **38** (2009).
4. D. M. Kingsley, K. F. Kozarsky, L. Hobble, M. Krieger, Reversible defects in O-linked glycosylation and LDL receptor expression in a UDP-Gal UDP-GalNAc 4-epimerase deficient mutant. *Cell* **44**, 749–759 (1986).
5. B. Schumann, *et al.*, Bump-and-Hole Engineering Identifies Specific Substrates of Glycosyltransferases in Living Cells. *Mol. Cell* **78**, 1–11 (2020).
6. J. Choi, *et al.*, Engineering Orthogonal Polypeptide GalNAc-Transferase and UDP-Sugar Pairs. *J. Am. Chem. Soc.* **141**, 13442–13453 (2019).
7. K. W. Moremen, *et al.*, Expression system for structural and functional studies of human glycosylation enzymes. *Nat. Chem. Biol.* **14**, 156–162 (2018).
8. E. Kowarz, D. Löscher, R. Marschalek, Optimized Sleeping Beauty transposons rapidly generate stable transgenic cell lines. *Biotechnol. J.* **10**, 647–653 (2015).
9. B. D. Berkovits, C. Mayr, Alternative 3′ UTRs act as scaffolds to regulate membrane protein localization. *Nature* **522**, 363–367 (2015).
10. L. Mátés, *et al.*, Molecular evolution of a novel hyperactive Sleeping Beauty transposase enables robust stable gene transfer in vertebrates. *Nat. Genet.* **41**, 753–761 (2009).
11. E. Lira-Navarrete, *et al.*, Substrate-guided front-face reaction revealed by combined structural snapshots and metadynamics for the polypeptide N-acetylgalactosaminyltransferase 2. *Angew. Chem. Int. Ed.* **53**, 8206–8210 (2014).
12. C. Peneff, *et al.*, Crystal structures of two human pyrophosphorylase isoforms in complexes with UDPGlc(Gal)NAc: Role of the alternatively spliced insert in the enzyme oligomeric assembly and active site architecture. *EMBO J.* **20**, 6191–6202 (2001).
13. J. B. Thoden, T. M. Wohlers, J. L. Fridovich-Keil, H. M. Holden, Human UDP-galactose 4-Epimerase. *J. Biol. Chem.* **276**, 15131–15136 (2002).
14. T. Kubota, *et al.*, Structural Basis of Carbohydrate Transfer Activity by Human UDP-GalNAc: Polypeptide α-N-Acetylgalactosaminyltransferase (pp-GalNAc-T10). *J. Mol. Biol.* **359**, 708–727 (2006).
15. C. Yu, L. Liang, Y. Yin, Structural basis of carbohydrate transfer activity of UDP-GalNAc: Polypeptide N-acetylgalactosaminyltransferase 7. *Biochem. Biophys. Res. Commun.* **510**, 266–271 (2019).
16. P. Emsley, B. Lohkamp, W. G. Scott, K. Cowtan, Features and development of Coot. *Acta Crystallogr. Sect. D Biol. Crystallogr.* **66**, 486–501 (2010).
17. N. W. Moriarty, R. W. Grosse-Kunstleve, P. D. Adams, Electronic ligand builder and optimization workbench (eLBOW): A tool for ligand coordinate and restraint generation. *Acta Crystallogr. Sect. D Biol. Crystallogr.* **65**, 1074–1080 (2009).
18. H. Xiao, E. C. Woods, P. Vukojicic, C. R. Bertozzi, Precision glycocalyx editing as a strategy for cancer immunotherapy. *Proc. Natl. Acad. Sci. U. S. A.* **113**, 10304–10309 (2016).
19. S. A. Malaker, *et al.*, The mucin-selective protease StcE enables molecular and functional analysis of human cancer-associated mucins. *Proc. Natl. Acad. Sci. U. S. A.* 116, 7278-7287 (2019).
20. A. R. Halpern, M. D. Howard, J. C. Vaughan, Point by Point: An Introductory Guide to Sample Preparation for Single-Molecule, Super-Resolution Fluorescence Microscopy. *Curr. Protoc. Chem. Biol.* **7**, 103–120 (2015).
21. M. Ovesný, P. Křížek, J. Borkovec, Z. Švindrych, G. M. Hagen, ThunderSTORM: A comprehensive ImageJ plug-in for PALM and STORM data analysis and super-resolution imaging. *Bioinformatics* **30**, 2389–2390 (2014).
22. C. M. Woo, *et al.*, Mapping and Quantification of Over 2000 O-linked Glycopeptides in

Activated Human T Cells with Isotope-Targeted Glycoproteomics (Isotag). *Mol. Cell. Proteomics* **17**, 764–775 (2018).
23. D. W. Morgens, *et al.*, Genome-scale measurement of off-target activity using Cas9 toxicity in high-throughput screens. *Nat. Commun.* **8**, 1–8 (2017).
24. K. Han, *et al.*, CRISPR screens in cancer spheroids identify 3D growth-specific vulnerabilities. *Nature* **580**, 136-141 (2020).
25. A. G. Grocin, R. A. Serwa, J. M. Sanfrutos, M. Ritzefeld, E. W. Tate, Whole proteome profiling of N-myristoyltransferase activity and inhibition using sortase A. *Mol. Cell. Proteomics* **18**, 115–126 (2019).
26. M. Rafiee, Mahmoud-reza Sigismondo, Gianluca Kalxdorf, B. Brügger, J. Béthune, J. Krijgsveld, Protease-resistant streptavidin for interaction proteomics. *Mol. Syst. Biol.* **in press** (2020).
27. A. D. Gracz, B. J. Puthoff, S. T. Magness, Identification, isolation, and culture of intestinal epithelial stem cells from murine intestine. *Methods Mol. Biol.* **879**, 89–107 (2012).
28. M. Fujii, M. Matano, K. Nanki, T. Sato, Efficient genetic engineering of human intestinal organoids using electroporation. *Nat. Protoc.* **10**, 1474–1485 (2015).
29. I. Lefebvre, *et al.*, Mononucleoside Phosphotriester Derivatives with S-Acyl-2-thioethyl Bioreversible Phosphate-Protecting Groups: Intracellular Delivery of 3′-Azido-2′,3′-dideoxythymidine 5 -Monophosphate. *J. Med. Chem.* **38**, 3941–3950 (1995).
30. H. Kim, J. K. Cho, S. Aimoto, Y. S. Lee, Solid-phase Staudinger ligation from a novel core-shell-type resin: A tool for facile condensation of small peptide fragments. *Org. Lett.* **8**, 1149–1151 (2006).
31. N. N. Biswas, *et al.*, Synthesis of antimicrobial glucosamides as bacterial quorum sensing mechanism inhibitors. *Bioorganic Med. Chem.* **25**, 1183–1194 (2017).
